# Peripheral priming induces plastic transcriptomic and proteomic responses in circulating neutrophils required for pathogen containment

**DOI:** 10.1126/sciadv.adl1710

**Published:** 2024-03-22

**Authors:** Rainer Kaiser, Christoph Gold, Markus Joppich, Quentin Loew, Anastassia Akhalkatsi, Tonina T. Mueller, Felix Offensperger, Augustin Droste zu Senden, Oliver Popp, Lea di Fina, Viktoria Knottenberg, Alejandro Martinez-Navarro, Luke Eivers, Afra Anjum, Raphael Escaig, Nils Bruns, Eva Briem, Robin Dewender, Abhinaya Muraly, Sezer Akgöl, Bartolo Ferraro, Jonathan K. L. Hoeflinger, Vivien Polewka, Najib Ben Khaled, Julian Allgeier, Steffen Tiedt, Martin Dichgans, Bernd Engelmann, Wolfgang Enard, Philipp Mertins, Norbert Hubner, Ludwig Weckbach, Ralf Zimmer, Steffen Massberg, Konstantin Stark, Leo Nicolai, Kami Pekayvaz

**Affiliations:** ^1^ Department of Medicine I, LMU University Hospital, LMU Munich, Germany.; ^2^ DZHK (German Centre for Cardiovascular Research), partner site Munich Heart Alliance, Munich, Germany.; ^3^ LFE Bioinformatik, Department of Informatics, Ludwig-Maximilians-Universität München, Munich, Germany.; ^4^ Vascular Biology and Pathology, Institute of Laboratory Medicine, University Hospital Ludwig-Maximilians University, Munich, Germany.; ^5^ Max Delbrück Center for Molecular Medicine (MDC) and Berlin Institute of Health (BIH), Berlin, Germany.; ^6^ Anthropology and Human Genomics, Faculty of Biology, Ludwig-Maximilians-Universität, Munich, Germany.; ^7^ Institute of Cardiovascular Physiology and Pathophysiology, Biomedical Center, Ludwig Maximilian University Munich, Planegg-Martinsried, Germany.; ^8^ Medizinische Klinik und Poliklinik II, University Hospital Ludwig-Maximilian University, Munich, Germany.; ^9^ Institute for Stroke and Dementia Research, University Hospital Ludwig-Maximilian University, Munich, Germany.; ^10^ Charite-Universitätsmedizin Berlin, Berlin, Germany.; ^11^ German Center for Cardiovascular Research (DZHK), Partner Site Berlin, Berlin, Germany.

## Abstract

Neutrophils rapidly respond to inflammation and infection, but to which degree their functional trajectories after mobilization from the bone marrow are shaped within the circulation remains vague. Experimental limitations have so far hampered neutrophil research in human disease. Here, using innovative fixation and single-cell–based toolsets, we profile human and murine neutrophil transcriptomes and proteomes during steady state and bacterial infection. We find that peripheral priming of circulating neutrophils leads to dynamic shifts dominated by conserved up-regulation of antimicrobial genes across neutrophil substates, facilitating pathogen containment. We show the TLR4/NF-κB signaling–dependent up-regulation of canonical neutrophil activation markers like CD177/NB-1 during acute inflammation, resulting in functional shifts in vivo. Blocking de novo RNA synthesis in circulating neutrophils abrogates these plastic shifts and prevents the adaptation of antibacterial neutrophil programs by up-regulation of distinct effector molecules upon infection. These data underline transcriptional plasticity as a relevant mechanism of functional neutrophil reprogramming during acute infection to foster bacterial containment within the circulation.

## Teaser

Transcriptional adaptation within the circulation is crucial for the execution of neutrophil defense functions.

## INTRODUCTION

Neutrophils are the most abundant leukocyte type in the human circulation and first responders to microbial intruders and sterile tissue injury alike (*1*–*4*). Impaired effector functions of neutrophils, including degranulation and neutrophil extracellular trap (NET) formation, as well as acquired or congenital neutropenia are associated with severe immune defects (*5*–*7*). Traditionally thought to be short-lived “foot soldiers” that uniformly react to pathogenic stimuli, recent studies in mice and humans have challenged the concept of a homogenous circulating neutrophil population (*4*). This heterogeneity includes surface expression diversity, dependent on neutrophil ageing, and a linked behavioral diversity (*8*–*14*). In the context of disease, this diversity translates to specific phenotypes, including immunosuppressive functions exerted by tumor-associated neutrophils (*15*, *16*) and myeloid-derived suppressor cells (*16*), as well as local and systemic hyperactivation of immature neutrophils (*17*–*20*).

Single-cell RNA sequencing (scRNA-seq)–based profiling approaches have benchmarked neutrophil heterogeneity allowing for the subsequent reproduction of neutrophil diversity using more scalable, pragmatic approaches like flow cytometry (FC) (*20*–*33*). To date, the phenotypic changes within the neutrophil compartment upon inflammatory stimuli are thought to depend on several factors, including a physiological “left shift,” referring to an expansion of younger neutrophil stages in peripheral blood, mediated by rapid bone marrow mobilization (*24*, *34*). While the egress from the bone marrow itself is associated with transcriptomic and proteomic changes (*35*), circulating neutrophils have been shown to also be influenced by their respective tissue environments after tissue recruitment (*36*). However, whether circulating blood neutrophils follow predefined trajectories (concept of “pre-committed programming”) or can be reprogrammed upon encountering peripheral cues (concept of “environmental programming”) remains under debate (*37*). While multiple environmental factors, like circadian rhythm, (re)entering and leaving the vascular bed as well as invading pathogens have been shown to affect neutrophil phenotypes, it is often unclear which signaling pathways are activated upon peripheral sensing of these factors (*37*). Further, there are no data showing convincingly that altered transcriptional activity of circulating neutrophils in mammals is acutely required for their functional capacity.

We hypothesized that circulating neutrophils respond to peripheral inflammatory cues in a highly adaptive manner, not merely through conventional functional responses and vesicle trafficking/receptor shuttling but primarily through a substantial up-regulation of antimicrobial gene sets.

Using an innovative toolset of transcriptome- and proteome-based profiling methods of fresh and cryopreserved neutrophils, we show that fundamental shifts in the neutrophil landscape result from Toll-like receptor (TLR4)/nuclear factor κB (NF-κB)–mediated priming of the readily circulating compartment and aggregate in non-predefined phenotypic and functional anti-microbial shift, driven by the prominent neutrophil effector protein CD177.

## RESULTS

### Single-cell RNA and epitope sequencing capture pronounced human neutrophil transcriptional plasticity across multiple maturation stages

**Fig. 1.**
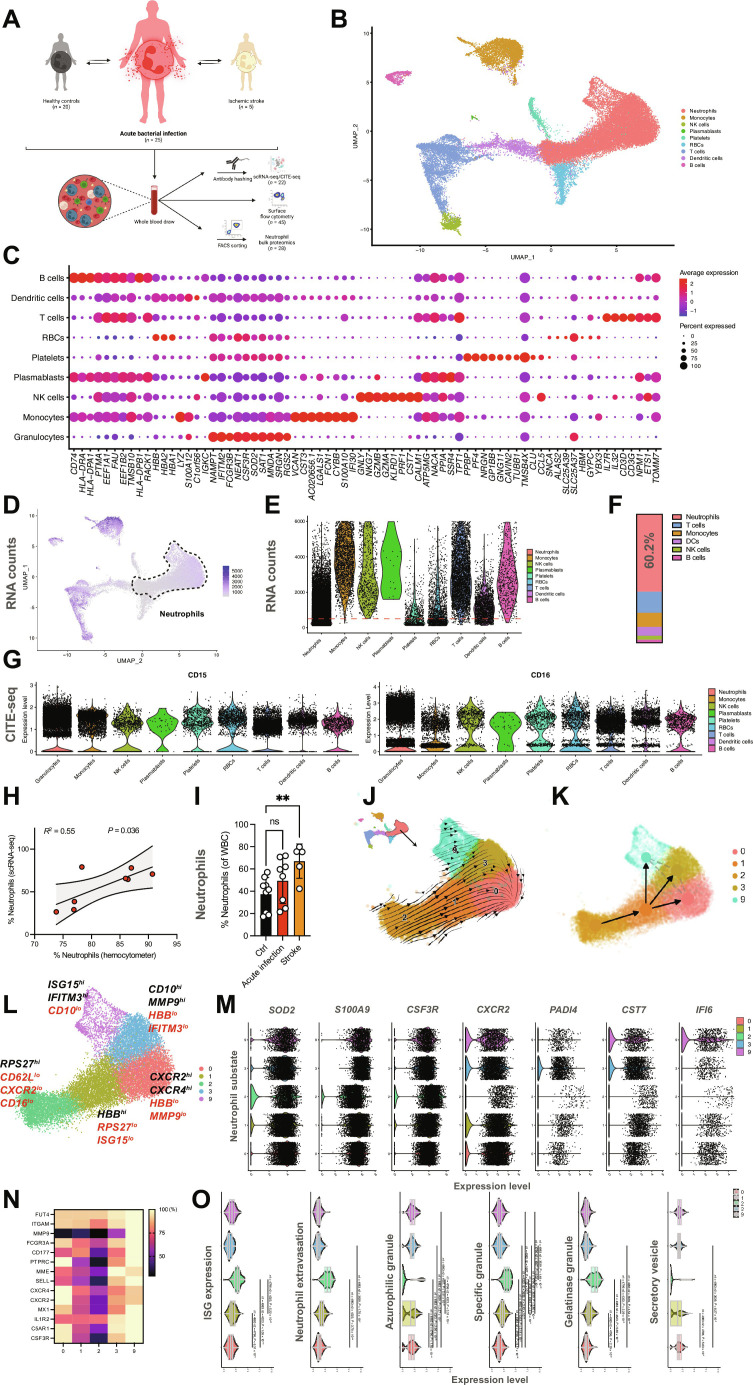
Integrative single-cell RNA and epitope sequencing captures the neutrophil landscape in health and inflammation. (**A**) Study design. (**B**) Integrative Uniform Manifold Approximation and Projection for Dimension Reduction (UMAP) of scRNA-seq data from human leukocytes (*n* = ∼35,000 cells and *n* = ∼20,000 neutrophils), with leukocyte populations clustered as indicated. NK, natural killer; RBCs, red blood cells. (**C**) Dot plot depicting expression of cell-type–defining genes. Compare fig. S1B for substate-defining genes of all 20 substates (0 to 19). (**D**) Feature plot of RNA content. (**E**) Violin plot depicting leukocyte RNA unique molecular identifier (UMI) counts. Red line indicates the UMI cutoff of 100 transcripts per cell. (**F**) Relative abundancy of leukocyte populations using scRNA-seq, as merged by leukocyte subset. DCs, dendritic cells. (**G**) Violin plots depicting the single-cell surface expression of CD15 and CD16 as assessed by CITE-seq, confirming substates 0, 1, 2, 3, and 9 as neutrophils. (**H**) Linear regression analysis of % of neutrophils as assessed by scRNA-seq versus the clinically detected relative amount of neutrophils of patients with acute infection included in the scRNA-seq part of this study. (**I**) Relative quantification of neutrophil subsets as detected by scRNA-seq. WBC, white blood cell; ns, not significant. One-way analysis of variance (ANOVA) with post hoc Kruskal-Wallis testing. (**J** and **K**) RNA velocity and partition-based graph abstraction analysis of neutrophil substates. (**L**) UMAP of neutrophil substates. Indicated genes reflect substate-defining genes differentially regulated in-between neutrophil substates (black, high expression; and red, low expression). (**M**) Expression of selected transcripts by substates 0, 1, 2, 3, and 9 depicted by violin plots. (**N**) Relative expression heatmap [normalized to maximum expression of respective transcript, (%)] of neutrophil surface markers. (**O**) Violin plots indicating module scores for indicated gene sets. Student’s *t* test, two-tailed, paired. Unless indicated with asterisks, post hoc testing revealed nonsignificant results (*P* ≥ 0.05). Unless indicated with asterisks, post hoc testing revealed nonsignificant results (*P* ≥ 0.05). *P* values corresponding to asterisks: **P < 0.01.

To capture plastic transcriptomic changes of readily circulating human neutrophil substates in both health and disease, we recruited patients with acute bacterial infection (*n* = 25) right after admission to the emergency department of our hospital (mean time from first presentation to inclusion 12 hours) and healthy individuals without signs of infection or inflammation (*n* = 20) and reanalyzed an independent dataset of blood from patients suffering from acute ischemic stroke (*38*) as sterile inflammatory controls (*n* = 5). Whole blood from these individuals (total *n* = 50 individuals) was processed for downstream applications, including scRNA-seq with fresh neutrophils, mass spectrometry, and FC with cryoconserved neutrophils (Fig. 1A and fig. S1, A and B).

We used a subset of 17 patients [*n* = 8 patients with acute bacterial infection, *n* = 9 healthy controls] to perform cellular indexing of transcriptomes and epitopes by sequencing at single-cell resolution (CITE-seq) (*39*), and integrated a reanalyzed published dataset of *n* = 5 patients with ischemic stroke (*38*). Because of low RNA abundancy of the five neutrophil substates (0, 1, 2, 3, and 9; Fig. 1, B to E, and fig. S1, C and D), we called cells on the basis of surface binding of anti-CD45 and major histocompatibility complex class I antibodies (*40*, *41*). This, in contrast to regular unique molecular identifier count filtering, enabled us to avoid loss of neutrophils with low RNA abundancy: Approximately 60% of captured leukocytes were neutrophils (Fig. 1F). CITE-seq cross-validated neutrophil identity and neutrophil abundancy correlated between routine clinical laboratory analyses and CITE-seq, which increased during inflammation (Fig. 1, G to I, and fig. S1, E and F). RNA velocity analysis and partition-based graph abstraction (*42*) of the identified neutrophil substates suggested a transition of substate 2 toward the more mature subpopulations 1, 3, and 9, with 0 appearing as the end stage of transcriptional development (Fig. 1, J and K, and fig. S1, G and H), as described before under healthy conditions (*35*).

Next, we analyzed transcriptomic heterogeneity among neutrophil clusters. All neutrophil subpopulations readily expressed classical neutrophil markers such as *CXCL8*, *SOD2*, *S100A8/A9*, *CSF3R*, and *MNDA*, and different substates were defined by differential expression of neutrophil maturity and effector markers (Fig. 1, L to O, and fig. S2, A to C; further described in Supplementary Materials and Methods).

### Circulating human neutrophils up-regulate genes encoding bactericidal proteins and activation markers in response to acute bacterial infection

**Fig. 2.**
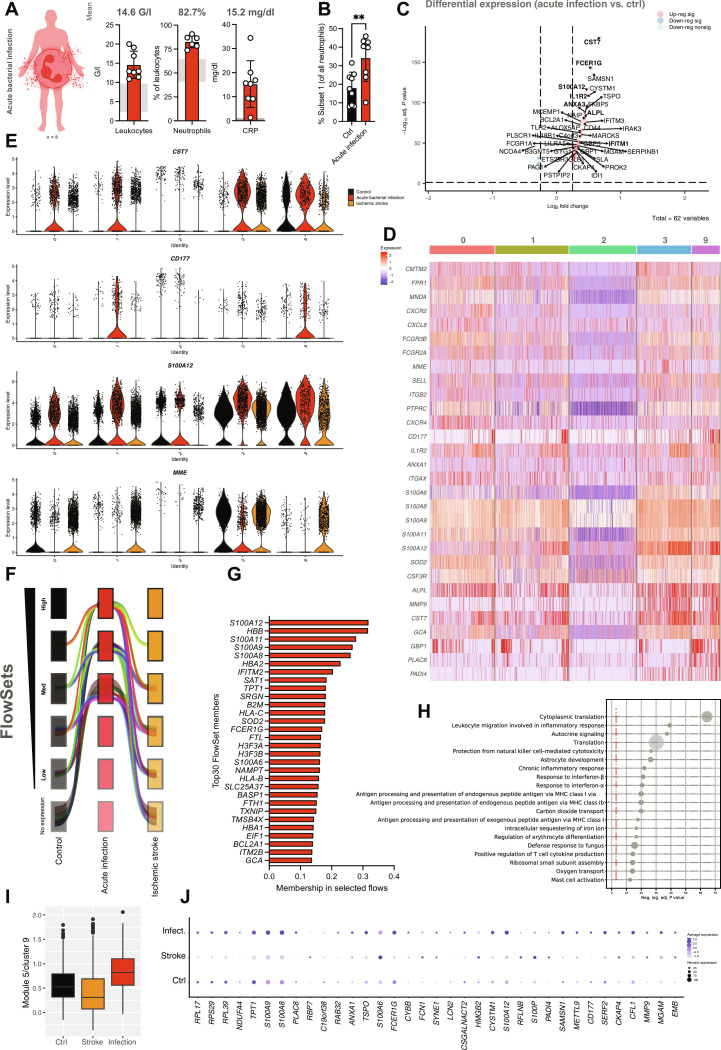
Substate-specific and global neutrophil transcriptomic responses to acute bacterial infection. (**A**) Clinical laboratory markers (leukocyte count, relative number of neutrophils and C-reactive protein (CRP) levels) from *n* = 8 patients with acute bacterial infection. Gray boxes indicate normal range (NR) of the respective parameters, numbers indicate mean value. G/l, giga per liter. (**B**) Relative quantification of substate 1 neutrophils in controls versus patients with acute bacterial infection. Student’s *t* test, two-tailed, unpaired. (**C**) Volcano plot depicting significantly differentially regulated genes across neutrophils clusters (0, 1, 2, 3, and 9). (**D**) Heatmap depicting the differential gene expression in response to acute bacterial infection in patients with acute bacterial infection compared to controls. (**E**) Violin plots depicting gene expression levels for the indicated transcripts across conditions (control versus acute bacterial infection versus ischemic stroke). (**F**) Visualization of FlowSets gene flows showing increased (minimum medium level) expression in all neutrophil subsets between patients with bacterial infection (red) compared to both healthy controls (black) and patients with ischemic stroke (orange). The width of each flow relates to the sum of all gene memberships within the flow. (**G**) Bar graph depicting the 30 genes with highest membership within the selected flows shown in (F). (**H**) Dot plot depicting Gene Ontology (GO) term analysis of the gene memberships of the selected flows shown in (F). The size of the dot correlates with the pathway size. MHC, major histocompatibility complex. (**I**) Visualization of mean module score of module 5 (substate 9 neutrophils), showing up-regulation of a distinct gene set in response to acute bacterial infection. (**J**) Dot plot depicting differential expression of transcripts included in module 5 of substate 9 neutrophils across healthy controls and patients with infection and stroke. Unless indicated with asterisks, post hoc testing revealed nonsignificant results (*P* ≥ 0.05). *P* values corresponding to asterisks: **P < 0.01.

We hypothesized that different neutrophil substates might respond distinctly to inflammatory cues. To assess the response of circulating human neutrophils to invading pathogens, we focused on individuals presenting to our emergency department with acute-onset bacterial infection (Fig. 2A) and compared these patients to both healthy controls and subjects suffering from ischemic stroke as a proxy of sterile inflammation (fig. S3, A to G). In line, clinical C-reactive protein (CRP) and leukocyte levels were elevated (Fig. 2A and table S1). Few patients (yet) had low CRP levels but already high leukocyte counts, mirroring early time points of disease onset. Peripheral neutrophilia was present in all patients with infection, particularly of substate 1 neutrophils (Fig. 2, A and B, fig. S3B, and table S1). In line, CITE-seq–based surface profiling revealed increased CD15 expression during infection, while other surface markers were not differentially expressed at surface level (fig. S3C).

Global changes included up-regulation of activation markers and antimicrobial genes like *CST7*, *S100A12, IL1R2,* and *ANXA1* (Fig. 2, C and D). Intriguingly, solely neutrophil substates 0, 1, 3, and 9, but not the younger subset 2, showed marked differential gene expression patterns in response to bacterial infection. Prominently, transcripts encoding the adhesion receptor CD177 were highly expressed in patients with bacterial infection (Fig. 2, D and E, and fig. S3, D to G) (*43*, *44*). Unsupervised bioinformatical analyses, including weighted gene cluster network analysis (*45*, *46*) and the innovative method FlowSets (see Supplementary Materials and Methods), confirmed infection-specific up-regulation of distinct gene clusters implicated in leukocyte migration, autocrine signaling, and inflammatory responses, as prominently marked by enhanced expression of *S100* genes (*S100A6, S100A8, S100A9, S100A11,* and *S100A12*) and granule contents (*GCA*; Fig. 2, F to J). In line, expression levels of several genes, particularly *CD177* and transcripts encoding S100 proteins, positively correlated with clinical severity as assessed by sequential organ failure assessment (SOFA) score, which was not the case for other bona fide neutrophil markers such as *CXCR1* or *CXCR2* (fig. S4, A and B) (*47*). These severity-associated increases in *CD177* expression were not dependent on either age or sex of the study group or with neutrophil substate abundance (fig. S4, C and D). This emphasizes that bacterial infection induces both substate-specific and shared, severity-dependent transcriptional responses across human neutrophil substates, including mature, circulating neutrophils.

### Marked up-regulation of CD177 protein levels characterizes the prominent peripheral shift during septic inflammation

**Fig. 3.**
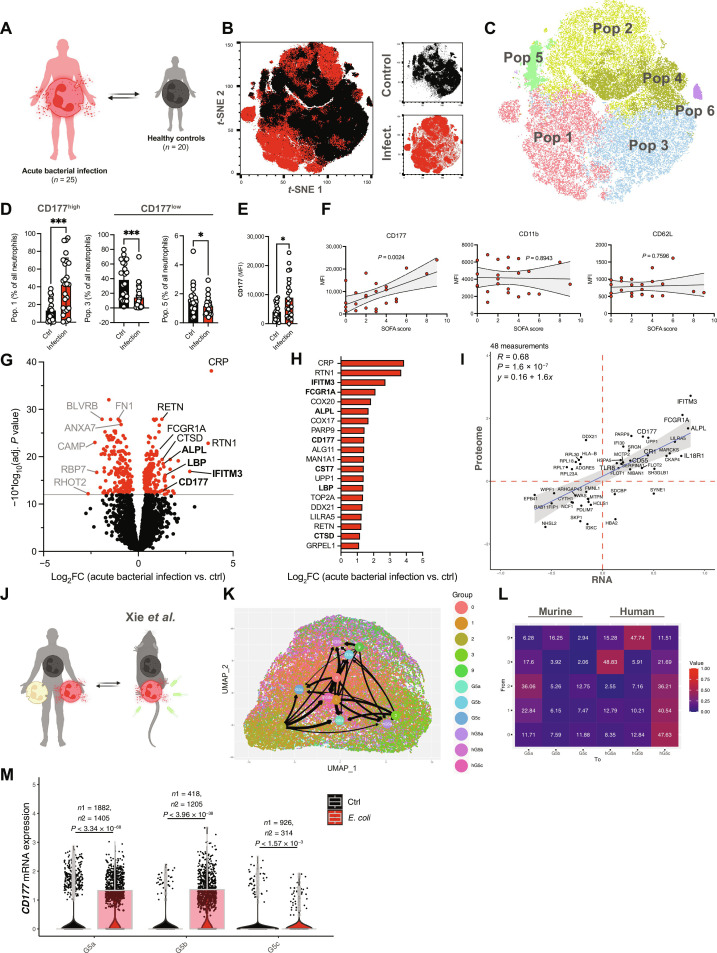
CD177 up-regulation at RNA and protein level following bacterial infection is conserved in mice and humans. (**A**) Overview of individuals in the confirmation cohort. (**B**) *t*-Stochastic neighbor embedding (*t*-SNE) plot comprising *n* = 900,000 neutrophils from 45 individuals. (**C**) *t*-SNE plot depicting neutrophil populations identified by FlowSOM. Populations comprising less than 3500 cells (i.e., <4% of all clusters across individuals) were excluded from further analysis. (**D**) Relative abundancies of CD177^high^ population 0 versus CD177^low^ populations 3 and 5 in controls versus patients. Student’s *t* test, unpaired, two-tailed. (**E**) Quantification of CD177 MFI across neutrophil FC substates. Student’s *t* test, two-tailed, unpaired. (**F**) Correlation plot of SOFA scores and neutrophil surface markers. (**G**) Volcano plot depicting differentially expressed proteins as assessed by mass spectrometry (red, adj. *P* < 0.05). (**H**) Fold change of 20 most up-regulated proteins in infected patients. Bold font indicates that corresponding transcripts are also up-regulated at transcript level in response to acute bacterial infection. Log_2_FC, log_2_ fold change. (**I**) Linear regression analysis of differentially regulated gene products at both RNA and protein level. (**J**) Schematic overview of comparisons of the scRNA-seq data from this study with murine scRNA-seq study by Xie *et al.* (*21*). (**K**) Integrative scRNA-seq UMAP of mature murine (G5a, G5b, and G5c) and human neutrophil substates (hG5a, hG5b, and hG5c, from *n* = 3 healthy individuals) from Xie *et al.* (*21*) integrated with neutrophil substates 0, 1, 2, 3, and 9. Arrow thickness indicates similarity of neutrophil substates. (**L**) Heatmap indicating the overlap of substates 0, 1, 2, 3, and 9 with murine and human substates G5a-c by Xie *et al.* (*21*) (**M**) Violin plots of *CD177* mRNA expression in response to acute bacterial infection in a mouse model of *E. coli* bacteremia. *P* values corresponding to asterisks: *P < 0.05, ***P < 0.005.

Next, we aimed to investigate whether the observed transcriptomic alterations in response to infection are reflected by the human neutrophil proteome. To capture changes in protein expression following altered gene expression in acute infection, we next designed a FC panel covering the key previously identified neutrophil substates (see table S3 for full panel). We analyzed patients with acute-onset bacterial infection (*n* = 25) and healthy controls (*n* = 20; Fig. 3A). The process of cryoconservation of paraformaldehyde (PFA)–fixated neutrophils did not severely impair surface antigen retrieval, did not induce any relevant differences in cell death (before the fixation step, since after fixation, neutrophils are eminently dead), and did not select for specific neutrophil activation markers compared to fresh blood neutrophils, emphasizing the robustness of our approach for neutrophil protein profiling and processing (fig. S5, A and B). We performed *t*-stochastic neighbor embedding (*t*-SNE)–based dimensionality reduction and subsequent substate identification using the FlowSOM algorithm (*48*). This suggested six major neutrophil populations (Fig. 3, B and C, and fig. S5, C to E). Some of these neutrophil populations mirrored transcriptomic alterations in substates identified by scRNA-seq: For instance, FC population 3 expressed high levels of CD15, CD62L, and interleukin-1 receptor type 2 (IL1R2), mirroring the transcriptomic signature of scRNA-seq substate 3, while FC population 6 revealed a protein profile similar to scRNA-seq substate 9, including high expression of the interferon-stimulated gene MX1 as well as surface receptors CD88 and colony-stimulating factor 3 receptor (CSF3R; fig. S5C). However, protein expression of several other receptors in the FC populations were not accompanied with increased transcriptomic expression of the same receptors (e.g., CXCR2 or CXCR4), likely reflecting other factors like receptor shedding and mobilization to the membrane that also affect the neutrophil proteome (fig. S5C).

In comparison to healthy control patients, patients responding to an acute bacterial infection showed a strong shift toward an enrichment of the CD177^high^ FC population 1. CD177^low^ FC populations, including FC populations 3 and 5, showed quantitative drops (Fig. 3D and fig. S5E). In general, neutrophils from infected patients showed significant increases in CD177 mean fluorescent intensities (MFIs) as well as elevated numbers of CD177^hi^ neutrophils (Fig. 3E and fig. S5F). Further, in accordance with *CD177* transcript levels, CD177 surface expression correlated positively with disease severity yet was independent of expression levels of maturity-associated proteins like CD10 or CXCR4 and classical activation markers like CD11b or l-selectin/CD62L (Fig. 3F and figs. S5G and S6).

We next aimed to assess the proteomic landscape of neutrophil-driven immune cell signatures across subsets in an unsupervised manner, using mass spectrometry (Fig. 3, G to I). Here, we detected differential expression of >300 gene products, including several proteins like interferon induced transmembrane protein 3 (IFITM3), alkaline phosphatase (ALPL), and CD177 that showed corresponding up-regulation at both transcript and protein level following acute bacterial infection (Fig. 3, H and I). Correlating genes with gene products in an integrative approach revealed a significant overlap in both transcript and protein regulation, suggesting direct proteomic consequences following transcriptomic changes (Fig. 3I).

Therefore, unexpectedly, neutrophil transcriptomic responses translated well into a respective shift in protein repertoire during bacterial inflammation, prominently in the well-known neutrophil effector protein CD177 (*44*, *49*–*56*).

### Mouse models of acute infection reveal a central role of peripheral priming for de novo neutrophil plasticity

We next assessed whether the observed transcriptional and phenotypic changes may be conserved across other mammalian species, rendering them amenable to further mechanistic investigation and therapeutic intervention. Recently, a landmark study by Xie *et al.* (*21*) described transcriptional heterogeneity of murine blood neutrophils in homeostasis and infection. We integrated our scRNA-seq neutrophil data with these murine datasets as well as three healthy human donors within the study (*21*). We found substantial similarities between neutrophil substates (Fig. 3, J to L; described in details in the Supplementary Materials and Methods). We further confirmed enhanced *CD177* expression in murine neutrophils following infection (Fig. 3M).

**Fig. 4.**
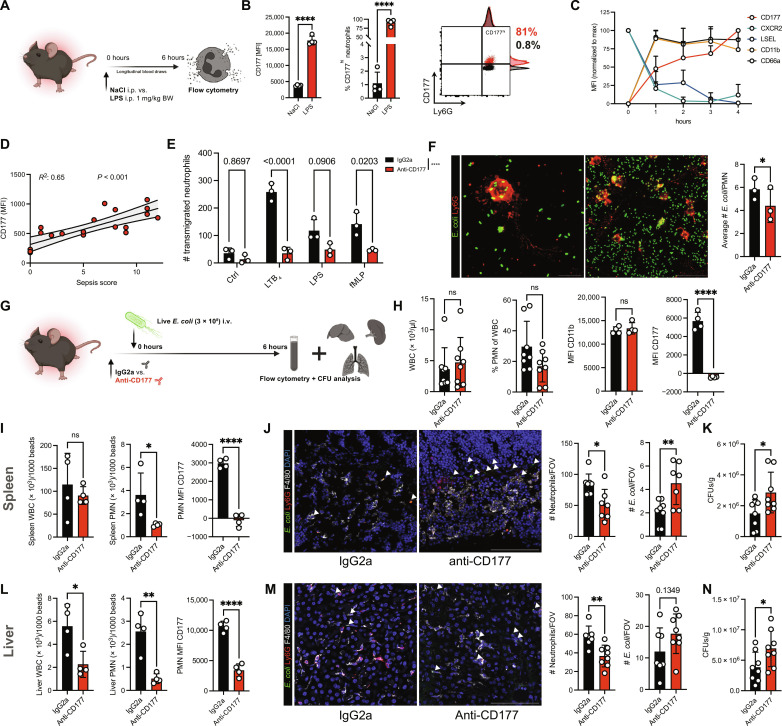
CD177 blockade increases bacterial dissemination through mitigating (trans)migratory capacity and phagocytic efficiency. (**A**) Experimental scheme. (**B**) Quantification of CD177 MFI and % CD177^hi^ neutrophils at 6 hours after LPS intraperitoneal (i.p.) injection. Right: Histograms of Ly6G and CD177 expression in neutrophils from NaCl- versus LPS-treated mice. (**C**) Longitudinal assessment of neutrophil surface markers relative to marker-specific maximal MFI. (**D**) Linear regression analysis of longitudinal sepsis scores and CD177 MFIs 0 to 4 hours after LPS intraperitoneal injection. (**E**) Quantification of transmigrated murine neutrophils in response to indicated stimuli. Two-way ANOVA with post hoc Dunnett’s testing. (**F**) Representative micrographs of neutrophils phagocytosing fluorescent *E. coli*. Scale bars, 10 μm (left) and 25 μm (right). Quantification of number of phagocytosed *E. coli* per neutrophils. Student’s *t* test, two-tailed, unpaired. See fig. S7F for FC-based quantification of phagocytosis. (**G**) Experimental scheme of murine bacteremia model through intravenous (i.v.) injection of live, green fluorescent protein–expressing *E. coli*. (**H**) Blood counts and neutrophil expression of select surface markers after 6 hours of incubation. Student’s *t* test, two-tailed, unpaired. PMN, Polymorphonuclear neutrophils. (**I**) Quantification of WBC, neutrophils/field of view (FOV) in the spleen, and CD177 MFI of splenic neutrophils as assessed by FC. (**J**) Representative confocal images of spleen sections. Scale bar, 100 μm. See fig. S8H for corresponding split channels. Quantification of neutrophils and *E. coli* per FOV. Student’s *t* test, two-tailed, unpaired. DAPI, 4′,6-diamidino-2-phenylindole. (**K**) Quantification of colony-forming units (CFUs) per gram of spleen. Student’s *t* test, two-tailed, unpaired. (**L**) Quantification of WBC, neutrophils/FOV in the liver, and CD177 MFI of hepatic neutrophils as assessed by FC. (**M**) Representative confocal images of liver sections. Scale bar, 100 μm. See fig. S8I for corresponding split channels. Quantification of neutrophils and *E. coli* per FOV. Student’s *t* test, two-tailed, unpaired. (**N**) Quantification of CFUs per gram of liver. Student’s *t* test, two-tailed, unpaired. *P* values corresponding to asterisks: *P < 0.05, **P < 0.01, ****P < 0.001.

Seeing these conserved transcriptomic features, we next set out to investigate the functional consequences of altered gene and protein expression in response to peripheral priming by monitoring the expression and functional relevance of the canonical activation marker CD177/NB-1 during acute inflammation. Despite previous mechanistic insights into CD177/NB1 function and its implications in the transmigration cascade (*19*, *44*, *53*–*58*), it remains unclear whether the identified phenotypic plasticity in effector protein expression, such as CD177, on circulating neutrophils could be attributed to peripheral priming. In addition, whether this plasticity carries functional consequences in clinically relevant disease models has not been investigated. Some studies have shown increased CD177 expression (among other markers) and related these to mobilization of early neutrophil stages upon inflammatory challenge (*51*, *59*). However, similar to shifts in the expression of other neutrophil effector proteins, it generally remains uncertain whether this up-regulation is (i) a result of a shift in abundancies of circulating neutrophil maturity stages, (ii) a result of predefined genetic programs imprinted in granulocytic precursors upon inflammatory challenge, or (iii) induced environmentally in already mobilized and mature subsets by context-dependent transcriptomic changes during acute inflammation. Given its previous role in governing neutrophil functions such as (trans)migration as well as our observations of consistent up-regulation at gene and protein level, we used interfering with CD177-mediated functions as a proof of concept to investigate whether transcriptional plasticity and subsequent up-regulation of the receptor are crucial for neutrophil-dependent host defense. Therefore, we used two mouse models of acute bacterial infection. In accordance with patients suffering from acute bacterial infection, mice injected with lipopolysaccharide (LPS) exhibited a notable up-regulation of neutrophil surface CD177 (Fig. 4, A and B, and fig. S7, A and B). Longitudinal assessment of CD177 expression by peripheral blood neutrophils upon septic inflammation in vivo revealed swift up-regulation and continuously rising expression of the surface receptor, significantly correlating with disease severity and showing LPS dose-dependent effects (Fig. 4, C and D, and fig. S7, C and D). This dose-dependent increase of CD177 expression in response to rising concentrations of LPS was associated with enhanced neutrophil influx into inflamed organs (fig. S7E). While LPS treatment also led to up-regulation of activation markers including CD66a and CD177 in Ly6G^+^ bone marrow neutrophils, CD177 expression even further increased upon adoptive intravenous transfer into LPS-treated animals and consecutive peripheral priming (fig. S7, F to H). We observed mitigated mouse neutrophil transmigration toward strong chemotaxins following antibody-mediated CD177 blockade, confirming previous reports from human neutrophils (*56*), as well as reduced phagocytic efficiency of neutrophils in vitro, and reduced calcium bursts upon exposure to chemotactic stimuli (Fig. 4, E and F, and fig. S7, I and J). Several other neutrophil effector functions were unaffected upon CD177 blockade in vitro (fig. S7, K to N, and movie S1).

**Fig. 5.**
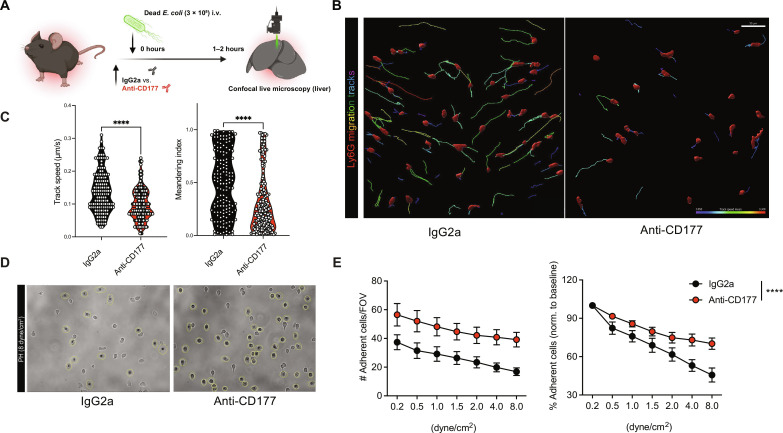
CD177 blockade impairs neutrophil (trans)migration in vivo. (**A**) Experimental scheme of confocal live microscopy following intravenous injection of *E. coli* bioparticles. (**B**) Representative rendered confocal images from in vivo microscopy as well as migration tracks (rainbow colors) for treatment groups. Scale bar, 50 μm. (**C**) Quantification of migration speed (μm/min) and meandering indices of neutrophils recruited to liver sinusoids. Student’s *t* test, two-tailed, unpaired. Cell-based analysis of 144 (IgG2a) and 202 (anti-CD177) individual neutrophils from *n* = 3 to 4 mice per group. (**D**) Representative bright-field (PH) images from detachment assays of IgG2a- or CD177-treated HoxB8-derived neutrophils treated with IgG2a (left) or anti-CD177 antibody (right) for 10 min before being perfused with increasing shear rates. (**E**) Quantification of attaching cells according to treatment and shear rate. Two-way ANOVA with post hoc Dunnett’s test. *P* values corresponding to asterisks: ****P < 0.001.

We next investigated the functional impact of CD177 in vivo using a translationally relevant model of acute bacteremia. Before intravenous injection of green fluorescent protein–expressing live *Escherichia coli*, mice were injected with either isotype immunoglobulins or antibodies blocking CD177 (Fig. 4G and fig. S8A). No differences in peripheral leukocyte counts were observed (Fig. 4H). FC analyses revealed specific and effective blockade of CD177 in peripheral blood (Fig. 4H and fig. S8B). We found increased pro-inflammatory cytokines in anti-CD177 antibody–treated animals, and blockade of CD177 was associated with a slower migration velocity and directionality in intravital microscopy, a significant reduction of neutrophil recruitment into the spleen and liver, leading to increased amounts of organ-dwelling *E. coli*, more colony-forming units in both spleen and liver, as well as elevated plasma levels of liver enzymes (Figs. 4, I to N, and 5, A to C, and fig. S8, C to J). This was accompanied by reduced detachment of anti-CD177–treated neutrophils under shear stress in vitro (pivotal for transendothelial migration; Fig. 5, D and E, and movie S2). Mechanistically, we found that antibody-mediated CD177 ligation elicited Src activation through phosphorylation, suggesting activation of β2 integrin as possible sequela of CD177 blockade with subsequently enhanced substrate adherence, as observed in human neutrophils (fig. S8, K and L). In summary, CD177, up-regulated in neutrophils in a septic environment, facilitates directed neutrophil (trans)migration and phagocytosis, pivotal for bacterial containment. Together, these findings underline the importance of the described phenotypic shifts in circulating neutrophils as critical contributors for anti-microbial effector functions in vivo.

### Peripheral plasticity of circulating neutrophil subsets coordinates phenotypic shifts

**Fig. 6.**
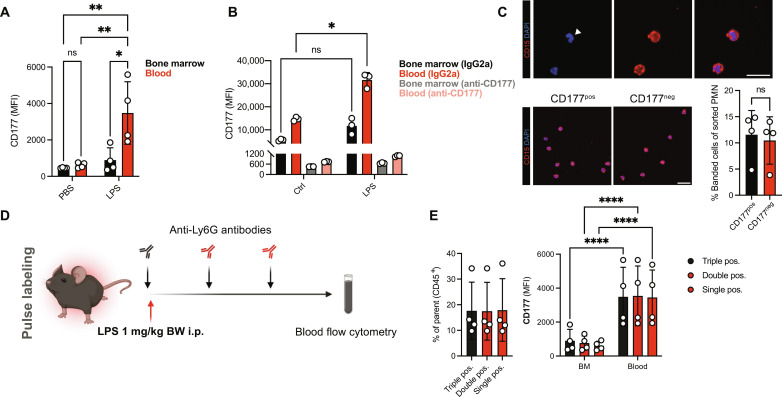
Peripheral priming in circulating neutrophils in vivo. (**A**) Quantification of CD177 MFIs of peripheral blood or bone marrow neutrophils. (**B**) Analysis of CD177 MFIs on isolated BM (black, gray) or blood neutrophils (red, light red) following incubation with LPS (10 μg/ml) after treatment with IgG2a or anti-CD177 antibody (10 μg/ml). (**C**) Representative immunofluorescence images and relative quantification of banded cells after sorting human neutrophils according to their CD177 expression (CD177^pos^ versus CD177^neg^). Top: Magnified example of banded cell. See fig. S11C for gating scheme. Scale bars, 20 μm. (**D**) Experimental scheme of neutrophil pulse labeling approach following LPS intraperitoneal injection. (**E**) Quantification of triple (FITC^+^, BV711^+^, and AF647^+^), double (BV711^+^ and AF647^+^), and single (AF647^+^) neutrophils of peripheral blood CD45^+^ cells. Right: Quantification of CD177 MFI of triple-, double-, and single-positive neutrophil isolated from peripheral blood or bone marrow (BM). Two-way ANOVA with Dunnett’s multiple comparisons test in (A), (B), and (E, right); one-way ANOVA Dunnett’s post hoc testing in (E, left); and Student’s *t* test, unpaired, two-tailed in (C). *P* values corresponding to asterisks: *P < 0.05, **P < 0.01, ****P < 0.001.

We next set out to substantiate that peripheral priming and subsequent transcriptomic changes induced in circulating neutrophils of any maturation stage could be responsible for the observed phenotypic shifts. Upon LPS challenge in mice, up-regulation of surface CD177 was pronounced in peripheral blood compared to the bone marrow compartment, as evidenced by an up to 10-fold increase of CD177 surface expression in peripheral compared to marrow neutrophils. This suggests peripheral priming and subsequent receptor up-regulation rather than mobilization of CD177^high^ marrow neutrophils as important contributors to the observed changes (Fig. 6A). This was corroborated by the observation that peripheral blood neutrophils exposed to LPS in vitro showed stronger up-regulation of CD177 in comparison to bone marrow neutrophils (Fig. 6B). In addition, bone marrow neutrophils exhibited lower baseline CD177 expression compared to circulating neutrophils (Fig. 6B). When we sorted human neutrophils according to CD177 expression, we did not find significantly different abundancies in banded neutrophils among CD177^pos^ or CD177^neg^ neutrophils (Fig. 6C and fig. S11C). Further, Ly6G antibody–mediated pulse labeling after sepsis induction with LPS did not reveal significant differences in CD177 expression in-between peripheral blood neutrophils labeled at different time points. However, it confirmed increased CD177 expression by blood compared to bone marrow–derived neutrophils (Fig. 6, D and E). This suggests that the observed increases in CD177 expression could be induced by peripheral priming, rather than being mainly attributable to infection-associated mobilization of bone marrow neutrophil pools.

### Disruption of de novo transcriptional plasticity in circulating neutrophils ameliorates phenotypic shifts and functional capacities of neutrophils upon septic inflammation

**Fig. 7.**
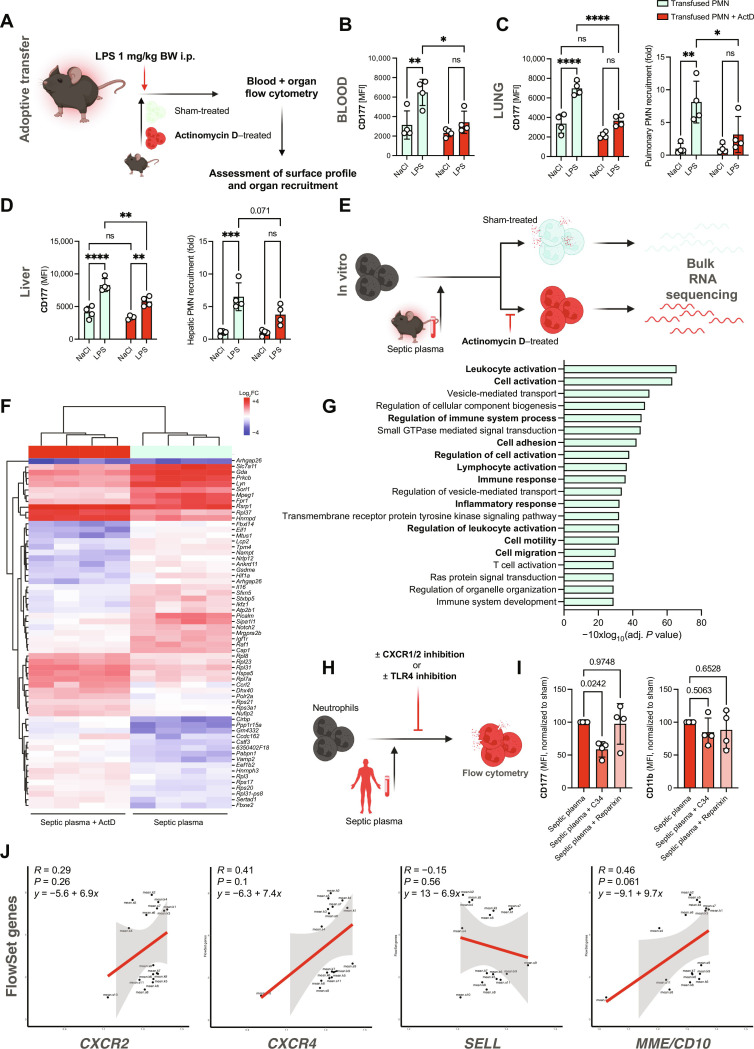
Peripheral priming of circulating neutrophils governs transcriptional changes and subsequent functional adaptations. (**A**) Experimental scheme of adoptive transfer experiment: Isolated neutrophils (5 × 10^6^) from donor mice were fluorescently labeled, treated with either vehicle or actinomycin D (ActD; 1 μg/ml), and subsequently infused into acceptor mice treated with NaCl or LPS [1 mg/kg body weight (BW)]. (**B**) Quantification of CD177 MFI of circulating, sham- or ActD-treated neutrophils in NaCl- or LPS-treated animals. (**C**) Quantification of CD177 MFI of sham- or ActD-treated neutrophils in the lungs of NaCl- or LPS-treated animals. Right: Relative quantification of pulmonary neutrophil recruitment according to pretreatment. (**D**) Quantification of CD177 MFI of sham- or ActD-treated neutrophils in the livers of NaCl- or LPS-treated animals. Right: Relative quantification of hepatic neutrophil recruitment according to pretreatment. (**E**) Experimental scheme of in vitro incubation of murine neutrophils with septic plasma in the presence or absence of ActD and subsequent bulk RNA-seq. (**F**) Heatmap of top differentially expressed genes sorted by adjusted *P* value (<0.05). *n* = 4 independent biological replicates were incubated with plasma samples from *n* = 4 independent LPS-treated mice. (**G**) Gene set enrichment analysis showing Top 20 up-regulated biological processes in sham- versus ActD-exposed neutrophils in response to septic plasma. (**H**) Experimental scheme of in vitro stimulation of healthy neutrophils with patient plasma after indicated pretreatment. (**I**) Quantification of CD177 and CD11b MFI. MFIs were normalized to neutrophils treated with phosphate-buffered saline due to interindividual baseline variance of CD177 expression. (**J**) Linear regression analyses of inflammation-associated FlowSet genes (see Fig. 3, F to H) and neutrophil *CXCR2, CXCR4, SELL*, and *MME* gene expression. Two-way ANOVA with Dunnett’s multiple comparisons test in (B) to (E) and one-way ANOVA Dunnett’s post hoc testing in (I). *P* values corresponding to asterisks: *P < 0.05, **P < 0.01, ***P < 0.005, and ****P < 0.001.

Last, to further elaborate on the concept of education mediated by peripheral inflammatory cues, we adoptively transferred pre-labeled murine neutrophils to investigate their in vivo responses. Before adoptive transfer, transfused neutrophils were pretreated with vehicle or actinomycin D (ActD), a potent RNA polymerase II inhibitor, to block inflammation-induced transcription of mRNA (Fig. 7A). Actinomycin did not interfere with key neutrophil functions in an acute setting, and survival of ActD-treated neutrophils in vitro was comparable to vehicle-treated neutrophils at the low concentrations used (fig. S9, A to D). Following sepsis induction in donor mice with LPS, both host neutrophils and sham-treated transfused neutrophils exhibited swift up-regulation of activation markers, including CD66a, CD11b, and CD177 (Fig. 7B and fig. S9, E and F). Notably, pretreatment with ActD at nontoxic concentrations mitigated CD177 up-regulation at protein level in comparison to transfused sham-treated neutrophils (Fig. 7B) (*60*). This disruption of the sepsis-induced transcriptome-proteome axis also resulted in reduced recruitment of ActD-treated neutrophils to target organs, including the lung and liver, emphasizing the importance of an intact transcriptional machinery for functional neutrophil behavior in vivo (Fig. 7, C and D, and fig. S9G). In support of these findings, murine neutrophils exposed to septic plasma in vitro showed reduced expression of inflammation-associated transcripts upon inhibition of RNA polymerase II (Fig. 7, E to G, and fig. S10, A and B).

**Fig. 8.**
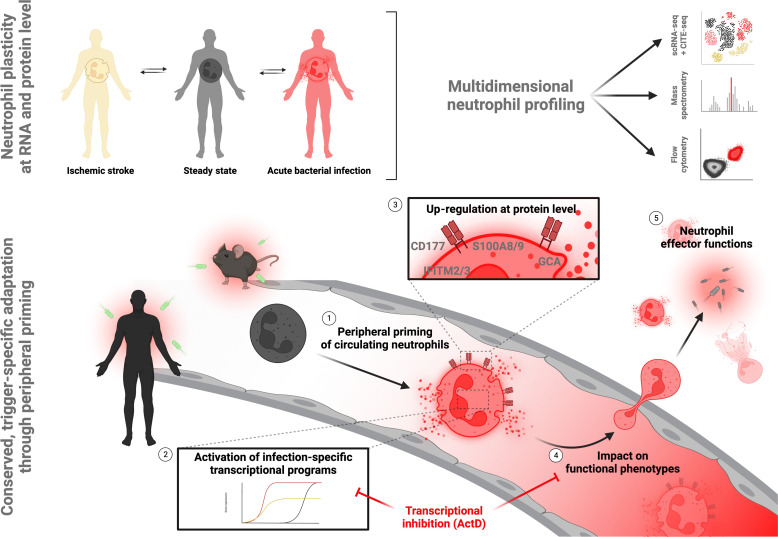
Graphical summary. Multidimensional profiling of human neutrophils in health and disease reveals neutrophil plasticity at transcript and protein level upon bacterial infection. This plasticity is induced through (1) TLR4/NF-κB–mediated peripheral priming of circulating neutrophils, leading to (2) differential transcript expression and subsequent (3) up-regulation of antimicrobial effector molecules at protein level. This altered gene and protein expression (4) affects functional phenotypes of circulating neutrophils and (5) contributes to neutrophil effector functions to limit bacterial dissemination. Figure created with Biorender.com.

Next, we investigated which circulating factors may contribute to the observed transcriptional shifts in sepsis. The chemokine receptors CXCR1 and CXCR2 as well TLRs, including TLR4 and its downstream effector proteins myeloid differentiation primary response 88 (MyD88) and signal transduction via NF-κB, are crucial contributors to neutrophil effector functions, including pathogen-associated molecular pattern (PAMP) recognition and NET formation (*17*, *61*–*66*). CD177 up-regulation was more pronounced in LPS-treated than* N*-formylmethionine-leucyl-phenylalanine (fMLP)–, zymosan-, or phorbol 12-myristate 13-acetate–treated neutrophils, indicating TLR4-mediated signaling as a putative culprit event in infection-associated peripheral priming (fig. S10C). In line, up-regulation of surface CD177 through septic plasma was blocked by preincubating human neutrophils with the TLR4 inhibitor C34, but not the CXCR1/2 antagonist Reparixin (Fig. 7, H and I). Further, blocking TLR4 and its downstream signaling through pharmacological blockade of MyD88 and NF-κB dampened CD177 up-regulation in murine neutrophils (fig. S10, D and E). Lastly, we found no correlation between expression of inflammation-induced transcripts and immaturity markers, but rather a trend toward a correlation of mature or aged neutrophil markers like CD10/*MME* or *CXCR4*, further strengthening the hypothesis that transcriptional plasticity of circulating, mature neutrophils upon peripheral priming contributes to acute immune responses (Fig. 7J). In summary, cross-species multimodal profiling confirms phenotypic and functional shifts in neutrophils during bacterial infection reflected by changes on the transcript and protein level, induced to a relevant extent by priming of peripheral neutrophils. This deems the plasticity of readily circulating neutrophils relevant for antimicrobial function and the observed phenotypic shifts. These shifts are at least partially induced by TLR/NF-κB signaling and contribute to neutrophil recruitment and subsequent directed (trans)migration through CD177 to secure bacterial containment in antimicrobial effector organs (Fig. 8).

## DISCUSSION

Mammalian neutrophils are essential contributors to the immune response in infection and sterile inflammation, but insights into their adaptability to external stimuli in peripheral neutrophils remain limited (*16*, *67*–*69*). Recent studies suggested that circulating neutrophils follow predetermined trajectories from hematopoietic progenitor cells and that shifts in circulating landscapes are primarily based on early imprinted programs at bone marrow precursor stages or due to mobilization of young substates (*22*, *35*). In contrast, it has been demonstrated that in vitro stimulation of mature neutrophils with inflammation-associated molecules affects also their transcriptomic output (*70*, *71*). Therefore, mechanisms underlying neutrophil shifts during inflammation in vivo remain incompletely understood, most prominently the relevance of peripheral education for altering the neutrophil landscape during inflammatory challenge (*37*).

We here used a CITE-seq–based, state-of-the-art single-cell sequencing approach in patients with bacterial infection, sterile inflammation, and respective controls to analyze de novo transcriptomic plasticity across blood neutrophil maturation stages. We subsequently transferred our findings to mass spectrometry–based shotgun proteomics, an FC panel in cryoconserved neutrophils and to functional assays to show that transcriptomic alterations directly translate to changes in the proteomic assembly and ultimately tune effector functions of circulating neutrophils. FC in combination with bulk whole-cell proteomics point toward de novo protein synthesis as a cause of the altered proteome, rather than surface receptor shuttling. To dissect neutrophil proteome, we established a toolkit showing high comparability between fresh and PFA-fixated, cryoconserved samples, enabling tracking of transcriptional neutrophil reprogramming to the protein level and allowing comprehensive phenotyping of conserved neutrophils.

Building on this resource, we monitored the canonical activation marker CD177 during acute inflammation to demonstrate how peripheral priming of readily circulating neutrophils drives transcriptomic, proteomic, and, ultimately, functional changes. Among the various up-regulated features at both transcript and protein level, we confirmed that CD177 stands out as one of the most prominently enriched effector proteins upon bacterial challenge upon older as well as young (recently mobilized) neutrophil subsets. We note that increased CD177 levels have been previously described in association with both sterile inflammation and bacteria-induced infection models (*19*, *44*, *49*, *52*, *58*, *72*, *73*). However, in line with the general debate on predetermined versus de novo neutrophil trajectories, it remained unclear which mechanisms are responsible for inducing these shifts.

Our transcriptomic data as well as in vivo adoptive transfer experiments suggest peripheral education of readily circulating neutrophils at the transcript level to profoundly contribute to these phenotypic shifts linked to the functional systemic needs. Not only inhibition of this transcriptional plasticity through interference with RNA polymerase activity but also blockade of TLR4 sensing and its downstream signaling hubs, including NF-κB and MyD88, swiftly dampened infection-driven neutrophil shifts and subsequent phenotypic changes. The intricate details of downstream CD177 signaling, including the contribution of complex formation with canonical interaction partners like proteinase 3 on the observed phenotypes (*74*–*77*), will, however, require further studies, including the use of genetically modified animal models. CD177 up-regulation, both at transcript and at protein/surface levels, was independent of neutrophil age within the circulation, as determined by an in vivo age-dependent tracking approach and by morphological analyses of banded/segmented nuclei. We found no correlation between expression of the mentioned markers (e.g., CXCR4 or CD10) with the transcriptomic features that defined the infection-induced neutrophil shift, nor did we find evidence of patient sex- or age-dependent influences on these shifts. Neutrophil-intrinsic transcriptional plasticity, hence, contributes to a relevant proportion of functional shifting during infection. This is supported by the notion that adoptive transfer of isolated neutrophils, but not neutrophils preincubated with the RNA polymerase inhibitor ActD, led to significant increases in CD177 protein levels under inflammatory conditions. Consequently, we observed relevant functional differences, i.e., reduced recruitment into target organs following transcriptional blockade, in line with specific CD177 inhibition. These data stress an unexpected level of adaptability of circulating neutrophils, which can up-regulate specific effector programs depending on the inflammatory challenge. The half-life of neutrophils in the human circulation is estimated to be ~19 hours, which is ample time to transfer transcriptional responses into functionally meaningful alterations of protein content (*78*). This ability to adapt the transcriptional program of the circulating neutrophil compartment adds another level of flexibility and complexity to the innate immune response. In addition to peripheral priming of already egressed, circulating neutrophils, mature neutrophils in the bone marrow, which have not yet physically egressed, might also be primed before mobilization. This could further affect to the observed plasticity of subsequently circulating blood neutrophil subsets. While our data including the observed dynamics of up-regulation of CD177 in acute infection models imply transcriptional plasticity as a crucial mechanism of CD177 up-regulation, previous work has highlighted complex epigenetic regulatory mechanisms that govern CD177 expression, including activator protein 1–induced promoter activation as well as (de)methylation-induced activation or silencing of the *CD177* locus (*79*, *80*). The contributions of these mechanisms to adaptation of *CD177* gene expression in the context of acute and chronic inflammation in humans will require further investigation.

This study holds limitations: Elucidating the interplay of these described influences on neutrophil phenotypes [i.e., unleashed egress from the bone marrow niche (*81*) and de novo peripheral priming] will require further analyses to allow for a detailed, neutrophil-substate resolved, comparative analysis of both mechanisms. Further, state-of-the-art methods that assess chromatin accessibility and the impact of transcription factors on the observed alterations in RNA content will shed light on the intracellular signaling cascades upon TLR4/NF-κB activation (*22*). We did not observe substantial effects on donor sex or age on the observed shifts at RNA and protein level. However, our sample size may be too small to allow for definitive conclusions as to whether either may affect the “primability” of circulating neutrophils. Further, the study cohorts show some differences in baseline characteristics, which are attributable to on-site sampling upon acute presentation. However, we note that this all-comer patient cohort is naturally diverse and representative of the spectrum of bacterial infection, including a broad bacterial spectrum as well as clinical states. Given the pronounced phenotype observed in murine infection models, our study raises the question how CD177-deficient individuals [up to 10% of Caucasians (*54*, *79*, *82*)] manage to contain microbial intruders in the absence of neutrophilic CD177 expression, especially in settings where neutrophils may need to (trans)migrate across endothelial or epithelial barriers to reach the culprit pathogen. There are no studies reporting immune deficiencies in CD177^null^ individuals. Wang and colleagues did not find differential recruitment of CD177^pos^ or CD177^neg^ neutrophils in human patients suffering from dialysis-associated bacterial peritonitis, although we note that their results may not be applicable to other infection modalities in patients without chronic kidney disease (*50*). Last, given the translational study design and the current lack of inducible fate mapping models for CD177^pos/neg^ neutrophils, further longitudinal assessment of patient biosamples will need to address whether acute inflammatory stimuli like invading pathogens can also lead to activation of previously silenced *CD177* loci, thus possibly affecting the proportion of circulating CD177^pos^ neutrophils that is thought to be a heritable trait and stable in individuals (*79*, *82*). We note that a previous study found notable increases in *CD177* mRNA expression in patients stimulated with granulocyte colony-stimulating factor, pointing toward either the up-regulation of the existing transcriptional patterns or the activation of previously silenced genes (*83*).

In summary, our study introduces innovative methodological high-throughput approaches allowing cryopreservation of neutrophils (as regularly performed with peripheral blood mononuclear cells) after PFA fixation with subsequent flow-cytometry and shotgun proteomics–based profiling.

Using these toolsets together with single-neutrophil sequencing, we propose that peripheral education by inflammatory cues and concomitant transcriptional responses observed in mammalian neutrophils are important contributors in shaping functional phenotypes in the setting of acute bacterial infection. The neutrophil is hallmarked by steady-state expression of antimicrobial proteins and pro-inflammatory granule contents. These data, however, show that peripheral priming of mature neutrophils and the subsequent implementation of transcriptional changes further affect the neutrophil proteome landscape in response to inflammatory cues with important implications for neutrophil effector functions and bacterial containment.

## METHODS

Detailed methodology is provided in the Supplementary Materials.

### Study cohort

A total of 50 participants were included in our study [*n* = 25 patients with proven or clinically suspected acute bacterial infection, *n* = 5 patients with sterile inflammation (ischemic stroke), and *n* = 20 healthy controls; see table S1]. All study participants provided a written informed consent in accordance with the Declaration of Helsinki and under the approval of the Ethics Committee of Ludwig-Maximilian-University Munich (nos. 20-1067, 121-09, and 20-0809). Participants were not compensated.

Patients with acute bacterial infection or sepsis [according to clinical judgement, routine laboratory studies, and SOFA score; (*47*, *84*)] were recruited within 24 hours of admission to the Emergency Department of LMU University Hospital and receipt of intravenous antibiotics (mean time between admission and inclusion 12 hours, *n* = 25). To investigate neutrophil heterogeneity in sterile inflammation, we additionally reanalyzed a scRNA-seq dataset of blood from patients with acute ischemic stroke undergoing interventional thrombectomy due to large vessel occlusion (*n* = 5) (*38*); this dataset is accessible under the following DOI: https://doi.org/10.5281/zenodo.10466854. In addition, we recruited healthy volunteers (*n* = 20) to serve as respective controls.

### Collection of whole blood, cryoconservation, and sample preparation for scRNA-seq

Fresh whole blood was collected from the study participants by peripheral venipuncture or, if available, through arterial or central venous lines, into lithium heparin tubes (Sarstedt). Samples were freshly processed for scRNA-seq (see below) or lysed and fixated using BD FACS lysing buffer as described previously (*19*). For cryoconservation, whole blood samples were incubated for 20 min in BD FACS lysing buffer with PFA at room temperature, spun down at 4°C (400*g*, 7 min) and resuspended in RPMI 1640 containing l-glutamine and 20% fetal calf serum (FCS) and 10% dimethylsulfoxide (DMSO). For scRNA-seq, 200 μl of whole blood were incubated with 2800 μl of BD lysing buffer (BD Biosciences, no. 555899) for 20 min. After centrifugation (350*g*, 7 min, 4°C), the pellet was blocked with anti-CD16/anti-CD32 antibodies (human BD Fc block, no. 564200) and incubated at 4°C for 7 min. The respective hashtag master mix (90 μl; final antibody concentration, 1:190; see table S2) was added and incubated for 30 min at 4°C. Subsequently, 5 ml of fluorescence-activated cell sorting (FACS) buffer [0.5% bovine serum albumin (Albumin Fraktion V, Carl Roth GmbH & Co. KG, no. 8076.4) + Dulbecco’s Phosphate Buffered Saline (Thermo Fisher Scientific, no. 14190-094)] was added and centrifuged at 250*g* for 10 min at 4°C. This washing step was repeated twice. After the last centrifugation step, the pellet was resuspended in 50 μl of FACS buffer. Cell counts were adjusted to 2200 cells/μl using a Neubauer counting chamber and then pooled. A total of 40 μl of the single-cell suspension was used for library preparation (input, 88,000 cells).

### Preparation of single-cell transcriptomic and surface libraries

We used the 10X Chromium Next GEM Single-Cell 3′ Reagent Kit with Feature Barcoding technology (CG000206 Rev. D). TotalSeq anti-human hashtag antibodies (B0251, A0252, and A0253) were used for feature barcoding of all patient samples.

The Chromium Next GEM Single-Cell 3′ Reagent Kit v3.1 (CG000206 Rev. D) from the 10X Genomics protocol was used. The Gel Beads-in-emulsion (GEMs) were prepared obtaining cDNA with reverse transcription. cDNA was purified, and an amplification and size selection were performed. After final quantification and quality control, the gene expression and cell surface libraries were constructed for sequencing, which was performed by using an Illumina NextSeq 2000.

### FC of fixated human whole blood

Fresh blood collected from study participants was erylysed and fixated using the FACS Lysing Solution (BD Biosciences) and frozen at −80°C in RPMI 1640 containing 10% DMSO and 20% FCS. For surface analysis by FC, samples were thawed, spun down, blocked with Fc block (anti-CD16/anti-CD32, BD), and stained for surface markers; for some panels, cells were permeabilized using the Permeabilization solution (BD, no. 554714) and subsequently stained with antibodies targeting intracellular proteins; a full list of all neutrophil markers included in this panel are shown in table S3. Samples were run on a LSRFortessa (BD Biosciences) flow cytometer, and measurement of all samples was performed on the same day in a randomized pattern to allow for MFI-based comparisons and unsupervised clustering of neutrophil populations. Gating strategies are depicted in fig. S11. Samples were analyzed using FlowJo v10.

For *t*-SNE and unsupervised clustering, neutrophils were downsampled using the downsample v3 plugin for FlowJo to 20,000 cells per sample (total of ~900,000 neutrophils from *n* = 45 individuals) and subsequently concatenated. All neutrophil markers were used as parameters for the *t*-SNE calculation. Unsupervised clustering was then performed on the expression values of all neutrophil markers using the FlowSOM algorithm (R version 4.2.1) (*48*) with a predetermined number of 12 meta-substates. Any meta-state containing <4% (i.e., total cell number < 3500) were not included in the analysis.

### scRNA-seq–based neutrophil subset description and comparison to murine subsets

Neutrophil substate 0, the most abundant substate, is characterized by the highest expression of *CXCR2* and *CXCL8*, along with Fc gamma receptors like *FCGR2A*, *FCGR3A*, and *FCGR3B*. In line with a more advanced maturity, substate 0 neutrophils express high levels of *CXCR4* (*85*). Substate 1 expressed proinflammatory markers (*GCA, IL1B*, *SOD2, C5AR1,* and *TNFRSF1B*) and interferon-stimulated genes (*IFITM2* and *MXD*) (Fig. 1M and fig. S2, A to C). Substate 2 neutrophils are defined by up-regulation of the translational machinery, including eukaryotic translation *EEF1A1* and ribosomal proteins, suggesting high translational activity in line with a younger substate (fig. S2B). Neutrophil maturation markers such as *CXCR2*, *MNDA*, or *FCGR3B*/CD16, CD10/*MME*, as well as l-selectin/*SELL* are less abundantly expressed in substate 2 (Fig. 1N). Neutrophil substate 3 exhibits a pro-inflammatory transcriptomic phenotype with up-regulation of *MMP9*, several S100 proteins, and genes promoting NET formation (*PADI4*, *HIST1H2AC,* and *H2AC6*; Fig. 1, L and M, and fig. S2A). Last, substate 9 neutrophils transcribe high levels of genes implicated in interferon signaling and was characterized by expression of activation markers like l-selectin (*SELL*) and *CD177*, respectively, and S100 proteins (Fig. 1, M and O, and fig. S2A).

Comparing both patient data and the reanalyzed data from Xie *et al.* (*21*), we find highly conserved populations, such as the ISG-expressing populations G5b (mouse), hG5b (human), and substate 9 (human) (Fig. 3, J to L). Similarly, substate 0 neutrophils show signatures comparable with populations G5c and hG5c, while cell state 3 neutrophils resembled murine and human populations G5a and hG5a (Fig. 3, K and L) (*21*). Shown violin plots are downsampled and include a similar number of cells per condition.

## Supplementary Materials

### This PDF file includes:

Supplementary Materials and Methods Tables S1 to S5 Figs. S1 to S11 Legends for movies S1 to S3 References
