# Supplementary material for: Peripheral priming induces plastic transcriptomic and proteomic responses in circulating neutrophils required for pathogen containment
Source: Sci Adv. 2024 Mar 22;10(12):eadl1710. doi: 10.1126/sciadv.adl1710 (PMC11093074; doi:10.1126/sciadv.adl1710)
Supplement: Supplementary file 1 — Supplementary Materials and Methods Tables S1 to S5 Figs. S1 to S11 Legends for movies S1 to S3 References [file sciadv.adl1710_sm.pdf]

Science Advances
AAAS

Supplementary Materials for

Peripheral priming induces plastic transcriptomic and proteomic responses in circulating neutrophils required for pathogen containment

Rainer Kaiser *et al.*

Corresponding author: Rainer Kaiser, rainer.kaiser@med.uni-muenchen.de;
Kami Pekayvaz, kami.pekayvaz@med.uni-muenchen.de

Sci. Adv. **10**, eadl1710 (2024)
DOI: 10.1126/sciadv.adl1710

The PDF file includes:

Supplementary Materials and Methods
Tables S1 to S5
Figs. S1 to S11
Legends for movies S1 to S3
References

Other Supplementary Material for this manuscript includes the following:

Movies S1 to S3

Suppl. Material and Methods

scRNA-seq processing

The raw reads were obtained from the sequencing facility. A total of 12 samples were processed using cellranger 6.1.1 with the 10X human reference data GRCh38 2020A and the provided hashtag antibodies as antibody capture. Processing of data was performed using R 4.0.1 and with Seurat 4.2.1(*86*). The resulting raw count matrices were read in and filtered (nFeature_RNA > 100, nFeature_RNA < 6000, nCount_RNA > 100, and percent.mt < 15) to exclude doublets and dead cells.

The Hashtag Antibodies were subset to only include relevant cells and normalized using CLR method. To call singlets and doublets, HTODemux was used according to the Seurat-provided Vignette with a positive quantile of 0.99.

The remaining CITE-seq antibodies were selected and added to a separate assay. These were normalized with CLR and margin = 2 (within a cell). This option was chosen because hashtag antibodies and CITE antibodies are competing and it cannot be assured that CITE antibodies were sequenced to saturation (as is the assumption with margin=1).

In preparation for integration, the library-wise Seurat objects were split into a list of one Seurat object per library and hashtag (patient-wise). Additional (pre-processed) Seurat object from the Ischemic stroke project (blood of samples 1,2,3,5 and 6, Pekayvaz *et al.*, *(38)*) were added to the list of Seurat objects of this project, which now contains one object per patient.

For each Seurat object, the RNA assay was normalized and variable features were identified. On these objects, 4000 integration features were calculated. As final preparation for integration, the cell-cycle score was identified and the data were scaled on the selected integration features while regressing out ribosomal and mitochondrial content, nCount_RNA and cell cycle). Finally, PCA was performed on the integration features. The integration itself took place on the 4000 integration features, using 50 principle components from the gene expression assay and the cca reduction method.

On the combined Seurat object (integrated by patient), first samples were annotated and PCA was performed with default parameters and subsequent calculation of the UMAP embedding (using the first 30 PCs). FindNeighbors was called with default parameters. Seurat's FindClusters was called with a resolution of 0.5. Gene expression was quantified for each cluster's marker genes, which were determined by Seurat's FindMarkers function using the t-test.

Differential gene expression analysis (also identifying marker genes) was performed on the RNA assay using the t test.

Volcano Plots were drawn using the EnhancedVolcano library (https://github.com/kevinblighe/EnhancedVolcano). Helping and plotting functions are now mostly available as part of the PLO(SC)^2 library (https://github.com/mjoppich/PLOSC).

Set enrichment was performed using clusterProfiler(*87*) and ReactomePA(*88*) on KEGG, Reactome or GeneOntology gene sets using over-representation analysis and p-value cut-offs of 0.05 and log2FC cut-offs of 0.25.

Specific gene set scores (see Suppl. Table 4) were calculated using Seurat's AddModuleScore function. Comparisons of score values between multiple clusters were performed using rstatix's pairwise_t_test function performing an unpaired two-sided t-test on the underlying scores per cell per cluster. P-values are Benjamini-Hochberg corrected.

Scores were correlated with log mean gene expression (data slot) of selected genes for clusters 0,1,2,3 (and 9) in each patient using ggplot2's stat_smooth function fitting a linear model.

The code of the scRNA-seq analyses is available online (https://github.com/mjoppich/neutrophil_sc).

scRNA-seq processing of Xie et al.(*21*) dataset (GSE137539)

The data were downloaded from GEO repository and read in together with the annotated meta data. Only cells falling into the (predicted.) clusters G5a, G5b and G5c (or their human counterparts) are selected.

After pre-processing all cells (our scRNA-seq data, human and mouse from Xie et al.(*21*) according to the filtering step described for our scRNA-seq data, all cells were integrated on the top 2000 integration features using the rpca method on 5 principle compoeenents, with a k.filter of 200 (FindIntegrationAchors) and k.weight parameter of 100 (IntegrateData). After the integration, PCA was performed, neighbours and clusters identified and a UMAP embedding calculated.

In the following we wanted to see how the neutrophil clusters from our scRNA-seq behave in comparison to the cells from Xie et al. *(21)*. First, identifying the centers of the clusters on the calculated UMAP representation is a good first start. However, distances in the UMAP embedding may not be fully preserved and interpretable.

Therefore, the relative frequency of neighbours in the Seurat neighbour tree falling into the 6 Xie et al. clusters (G5a-hG5c) was determined and annotated on the UMAP as arrows between cluster centers *(21)*. The larger an arrow is, the higher the fraction of neighbours between the two clusters. The fractions are also visualized in a heatmap.

Velocity analysis

For the velocity analysis, the barcodes of all cells in the integrated Seurat object were written to disk per library. This list of barcodes is important for running velocyto(*89*) if also barcodes are used that are not part of the filtered barcodes from cellRanger. Apart from specifying the barcodes of the required cells, velocyto was run with default parameters. After performing the counting of unspliced RNAs using velocyto, the acquired loom files were matched with the integrated Seurat object and the unspliced count matrix is written to disk together with a loomified version of the integrated Seurat object and additional meta information (e.g. UMAP coordinates). In the second step, this object is read using scvelo(*90*). The UMAP embedding is taken over and spliced (cellRanger counts) and unspliced (velocyto) counts are stored in their respective layers. Genes that were not present in at least 50 cells were filtered out and the remaining matrix was normalized. 3000 highly variable genes were called using scanpy's(*91*) default parameters, upon which moments (n_pcs=30 and n_neighbors=30) and velocities are calculated.

Before applying partition-based graph abstraction to the data, first velocity pseudotime was calculated. Following this, dynamics were recovered, latent time calculated and velocity re-calculated using differential kinetics, for which paga is called again. For the top likelidhood genes a heatmap was plotted.

The source code of the scRNA-seq data analysis will be made available together with the preprocessed Seurat object upon publication.

FlowSets

(Differential) Expression data are read in for each gene and each cluster (or: state). The values are fuzzified either by user-defined membership classes, or equally distributed over the measurement range (min-max), or according to predefined quantiles. Fuzzification is performed either by triangular or gaussian membership function. The gene expression is fuzzified by first deriving the expression distribution of the cell (mean and standard deviation of all expressing cells, and fraction of non-

expressing cells), and then translating this distribution into memberships by sampling from the created distribution.

Relevant flows can be defined and selected using a simple grammar with the flow_finder function, where the desired difference between two levels can be specified. For each flow, or a group of flows, gene set enrichment analysis can be performed. Here, the gene sets are binned according to their size, e.g. all gene sets with at least 2 and at most 5 genes are put together into one bin. For each bin and gene set, all flow memberships are accumulated and divided by the number of genes in the gene set (pw_cov). For each pw_cov score the z-score is calculated (how different is a gene set from all other gene sets of that bin), which is transformed into a p-value for all positive-z-score (=higher accumulated membership than expected) gene sets.

For this study, gene expression data were used for representing fuzzy flows between control, ischemic stroke and bacterial infection. The expression levels NO, LOW, low, med(ium), high and HIGH expression represent the expression data of the cluster 0,1,2,3,9 neutrophils. The expression level centers are set to log-expression values of 1,2,3,4,5,6 using triangular membership functions. For gene set enrichment both Reactome and Gene Ontology Biological Process gene sets were used(88*, 92*).

For more detailed information on the FlowSet algorithm, see https://github.com/mjoppich/Flowsets.

Gene module detection

We performed temporal gene module detection using the method and script provided by Kazer et al.(*47*). In brief, this method takes the gene expression values of the genes represented by the first few principal components of the scRNA-seq object as input for WGCNA functions. Here, we chose the maximal 250 genes reported by the PCASigGenes function of Seurat for each of the first eight PCs. The resulting adjacency matrix (created with soft power 7 and minimal module size 3 is transformed into a TOM and hierarchically clustered. These clusters are merged if not too dissimilar. After testing the modules for their significance (p-Value threshold of 0.05, 10 bins and 100 permutations), the remaining modules are tested regarding the variation between control, sepsis and stroke samples. The remaining modules are added to the Seurat object using the AddModuleScore function (ctrl set to 5) and are reported for further visualization and discussion.

LR-based Interaction Analysis

Chemokine interactome analysis. Relevant ligand-chemokine receptor interactions were collected from Shilts et al.(*93*). In general, the steps described by Armingol et al.(*98*) are followed for determining cell-cell communications. The experimental expression data (for each cluster) is read in and filtered to only contain genes from the above collection of LR interactions. For each ligand-receptor pair, and for each cluster-pair, the communication score is calculated. This communication score is the product of the logged average ligand expression and the receptor expression (expression product).

This results in a data frame in which for each ligand-receptor pair in each cluster pair a score is associated. To determine the total communication between two clusters, all communication scores between these clusters are aggregated (sum) if the communication score is above a threshold (here: 3).

In a second step, the data frame is arranged into matrix form, keeping only those clusters of interest (or all, if no filtering was requested). The chord diagram (taken from https://github.com/tfardet/mpl_chord_diagram) shows displays then the accumulated LR-interactions between clusters (all, or subset to only for monocytes and neutrophils). In addition, the matrix plot shows the scaled (z-score) expression scores for all interactions in the selected clusters. In a filtered version, only interaction which have at least in one cluster pair a z-score > 1 are shown. Finally, the chemokines overview displays the ligand-receptor map in the lower left corner, and shows the expression values for the receptors in the selected clusters on top, and those for the ligands to the right. This visualization allows for a brief concise overview of ligand and receptor expressions, while also showing possible interactions together with an overview of possible LR-interactions. Source code for this analysis is available online https://github.com/mjoppich/chemokine_interactionmap.

Bulk RNA sequencing of murine neutrophils

The RNA-sequencing was carried out utilizing the prime-seq method developed by Janjic et al (*94*). The complete protocol for prime-seq, along with the primer sequences, can be accessed at protocols.io (https://www.protocols.io/view/prime-seqs9veh66). In summary, the process involved transcribing cDNA using MaximaH Minus reverse transcriptase, oligo dT primer E3V7NEXT, and template switching oligo. The cDNA obtained from each species samples was pooled and subjected to

preamplification using KAPA HiFi HotStart polymerase and SingV6 primer. Libraries were constructed from 21 ng of cDNA, employing the NEBNext Ultra II DNA Library Prep Kit for Illumina (New England Biolabs), and dual indexing with TruSeq i5 index primer and Nextera i7 index primer. Sequencing was conducted on an Illumina NextSeq 1000/2000 instrument, configured with the following parameters: read 1 (28 bases), read 2 (8 bases), read 3 (8 bases), and read 4 (93 bases).

Data quality was assessed using fastqc (version 0.11.8113) (*95*). Cutadapt (version 1.12114) (*96*) was used to remove any regions on the 3′ end of the read if the sequenced read reached into the polyA tail. Hereafter, filtering of the data was performed using the zUMIs pipeline (version 2.9.4d, Parekh et al., 2018), with a phred threshold of 20 for 4 bases for both the UMI and BC. Reads were mapped to the human genome (GRCh38) with the Gencode annotation (v35) using STAR (version 2.7.3a) and quantified using RSubread (version 1.32.4) (*97*).

Processing of bulk RNA sequencing data

The zUMIs counted UMI-counts considering intronic and exonic alignments were used for differential gene expression analysis between samples incubated with septic plasma compared to samples pre-incubated with actinomycin D prior to septic plasma incubation, which consist of each 4 replicates. For this, DESeq2 (v1.38.1)(*98*) was used in R v4.2.2, being called from the poreSTAT differential expression pipeline (https://github.com/mjoppich/poreSTAT). A total of 759 genes is significant differentially regulated (adj. P-value < 0.05 and abs(logFC) > 0.25), of which 207 genes are down-regulated and 552 up-regulated. On the identified significant differentially expressed genes (adj. P-value < 0.05 and abs(logFC) > 0.25), gene set enrichment analysis was performed using clusterProfiler v4.6.0 on all significant differentially expressed genes and both the up- and down-regulated genes as part of the poreSTAT differential expression pipeline (*99*).

Fluorescence-activated cell sorting (FACS)

Fresh blood collected from study participants was lysed and fixated using FACS Lysing Solution (BD) and frozen at -80°C in RPMI containing 10% DMSO and 20% fetal calf serum (FCS). Prior to sorting, samples were thawed, spun down, blocked and stained for CD45, CD15 and CD11b. Neutrophils were subsequently sorted on a BD

FACSMelody benchtop sorter (gating: size > singlets > $CD45^+$ > $CD15^+$ $CD11b^+$). For each sample, 250,000 neutrophils were sorted into PBS, spun down, resuspended in 2% SDS and 50 mM TRIS and boiled at 95°C for 10 min prior to storage at -80°C. See Fig. S11 for gating scheme.

Sample preparation for mass spectrometry and analysis.

To analyze the proteome of human neutrophils we applied SDS based lysis and SP3 cleanup as described (*100, 101*). Samples were measured on an orbitrap Exploris 480 instrument (Thermo Fisher Scientific) in label-free data-independent acquisition (DIA) mode whilst separating peptides on a 44 min gradient on a nanoEASY 1200 system (Thermo Fisher Scientific) coupled to the mass spectrometer. Raw files were analysed in DIA-NN version 1.8.1 using a library free search strategy (*102*). An FDR cutoff of 0.01 was applied and spectra were searched against a human Uniprot database from 2022 including isoforms whilst applying the MaxLFQ algorithm for protein quantitation(*103*). Further downstream analysis was performed in R. Data filtering by rows was carried out requiring at least 75% valid values across all samples before applying median-normalisation and imputation based on a randomised normal distribution with a width of 0.3 and a downshift of 1.8. Two-sample moderated t-statistics were calculated using the limma package(*104*). Nominal P-values were corrected using the Benjamini-Hochberg method.

Human blood donors

Female and male volunteers aged 22 to 50 years served as donors for the isolation of neutrophils and healthy plasma. All experiments involving human participants are approved by the local ethical review board of LMU Munich and comply with any relevant regulation for experiments involving human biosamples.

Isolation of human and murine live neutrophils

Whole blood was drawn through venipuncture and human neutrophils were isolated using a bead-based kit (StemCell EasySep Human Neutrophil isolation kit), resuspended in RPMI and counted using an automated cell counter (Sysmex XN V Series XN-1000V). For mouse neutrophil isolation, wild-type mice were sacrificed, and femoral and tibial bone marrow were collected. Neutrophils were subsequently isolated using a bead-based kit (Miltenyi Biotec Neutrophil isolation kit), resuspended in RPMI

and counted using an automated cell counter. For some assays, whole blood was collected in anaesthetized mice by retroorbital puncture in order to isolate peripheral blood neutrophils using the same kit.

Neutrophil transmigration assay

Human or mouse neutrophils were isolated as described above and stained using the CellTrace™ CFSE Cell Proliferation Kit (Invitrogen, 1:2,000) for 10 min, followed by a washing step. $1x10^5$ neutrophils were subsequently allowed to attach to the upper part of a 3 µm transwell insert (Sarstedt), and the transwell insert was transferred to a 24 well plate containing RPMI + 0.1% FCS mixed with either PBS or neutrophil chemotactic agents (LPS, 10 µg/ml; LTB_4, 100 nM; TNFalpha, 1 µg/ml; fMLP, 10 µM). Transwells were incubated for a total of 2 hours, and confocal images of each well were acquired hourly during incubation at 37°C, 5% CO_2.

Neutrophil phagocytosis assay

Ibidi µ-slides (0.4, IbiTreat) were coated with poly L-lysine (ThermoFisher) for 10 min and washed with PBS. Fluorescent heat-killed E. coli (Escherichia coli BioParticles, Invitrogen/ThermoFisher, $2x10^6$) were added to the chamber and spun down at 350 G, 4°C for 7 min, resulting in a homogenous layer of bacteria. Mouse neutrophils were isolated as described above, stained with anti-Ly6G antibody (1:100), added (10^5 per well) to each slide, allowed to settle and then incubated for 2 hours at 37°C, 5% CO_2. Samples were then fixated with 2% PFA and 0.05% GDA. Random images (6x) were taken of each chamber. Neutrophils and phagocytosed E. coli particles were automatically counted using a custom macro in Fiji as described previously. Large bacterial clumps were assessed by Z-stack imaging and the intracellular counts were subsequently curated manually.

NET formation assay

NETs were induced as previously described(*17, 105*). In brief, murine neutrophils were isolated from peripheral blood using the Miltenyi Biotec Mouse Neutrophil isolation kit as described above; neutrophils were pooled from two mice per biological replicate to achieve sufficient cell numbers. Human neutrophils were isolated from peripheral blood as described above. Following counting, $1\text{-}2x10^5$ neutrophils were seeded onto circular glass slides coated with poly L-lysine. After attachment, neutrophils were treated with

either isotype or anti-CD177 (MAB8186, 10 µg/ml) and subsequently exposed to 500 nM phorbol myristate acetate (PMA, Sigma) for 2-4 hours. Neutrophils were then fixated using 1% PFA final conc., permeablized with Triton X-100 (0.1%) and stained Hoechst dye, SytoxGreen (1:5000, ThermoFisher) and anti-Ly6G antibody (Biolegend, see above). Glass slides were then mounted on a microscopy slide covered with Dako mounting medium and imaged on a Zeiss LSM 880 confocal microscope using 6-8 random areas per slide. % of NETing neutrophils were assessed as described previously(*17, 105*).

Flow cytometry-based phagocytosis assay

Mouse neutrophils were isolated as described above and stained with anti-Ly6G antibody (1:100). Following counting, $2x10^5$ neutrophils were seeded into a 96 well plate. After attachment, neutrophils were treated with either isotype or anti-CD177 (MAB8186, 10 µg/ml) and subsequently incubated with pHrodo Green BioParticle ($2x10^6$, ThermoFisher) for 90 minutes at 37°C, 5% CO2. Phagocytosis was assessed by flow cytometry. The phagocytic index was calculated using the following formula: $\frac{Neutrophils\ with\ pHrodo\ E.coli}{All\ neutrophils} * MFI\ (E.coli)$.

Reactive oxygen species (ROS) production assay

Mouse neutrophils were isolated as described above and stained with anti-Ly6G antibody (1:100). Following counting, $2x10^5$ neutrophils were seeded into a 96 well plate. After attachment, neutrophils were treated with either isotype or anti-CD177 (MAB8186, 10 µg/ml) and subsequently loaded with 2',7' DCFDA (1mM, Sigma). Finally, cells were exposed to PMA (100nM, Sigma) for 15 minutes at 37°C, 5% CO2, followed by immediate flow cytometric analysis.

Intracellular calcium assessment

The protocol for analyzing intracellular $Ca2^+$ levels in Neutrophils followed the method recently described by Bai et al (*56*). Initially, mouse neutrophils were isolated as described above, stained with anti-Ly6G antibody (1:100) and then loaded with Fluo4 $Ca2^+$ indicator (1:1000, ThermoFisher). Subsequently, neutrophils were treated with either isotype or anti-CD177 (MAB8186, 10 µg/ml) in RPMI1640 medium containing 0.1% FBS and 5 mM EGTA for 30 minutes at room temperature. Flow cytometry analysis was performed at baseline and immediately after stimulation of cells with fMLP

(10µM). The mean fluorescence intensity for every 10-second interval after stimulation was calculated as fold change relative to the baseline value.

Plasma incubation of human and murine neutrophils

Co-incubation of neutrophils from healthy human donors or WT mice with plasma from healthy individuals and septic patients or sham-treated and septic mice, respectively, was performed as described previously(*17*). In brief, following isolation, neutrophils resuspended in RPMI were incubated with 20% plasma for 6 h at 37°C in the presence or absence of the indicated inhibitors. Live neutrophils were identified as SYTOX-negative cells positive for classical neutrophil markers such as CD15+ CD11b+ (human) or Ly6G+ CD11b+ (mice). Unless indicated otherwise, absolute MFIs were used for statistical analysis.

Neutrophil flow chamber using HoxB8-derived cells

Expression of CD177 by differentiated HoxB8-derived neutrophils was validated by immunofluorescence staining (see Fig. S7M). Neutrophil adhesion and rolling as well as detachment were analyzed *in vitro* using microflow chambers (µ-slides $IV^{0.1}$, IBIDI) as described previously (*105*).

In brief, microflow chambers were coated with P-selectin, ICAM-1 and CXCL1 for 12h at 4°C and blocked for 2h at RT. Prior to perfusion, HoxB8-derived neutrophils were suspended in adhesion medium (HBSS supplemented with 20 mM HEPES, 0.25% BSA, 0.1% glucose, 1.2 mM $CaCl_2$, and 1.0 mM $MgCl_2$) and incubated with either isotype (IgG2a) or anti-CD177 antibody (10 µg/ml) for 10 min. Subsequently, 7.5×10^5 cells/ml were perfused through the flow chamber at constant shear stress (1 dyne/cm^2) using a high-precision syringe pump (model KDS-232, KD Scientific). Time-lapse videos were recorded with a Zeiss Axiovert 200M microscope equipped with a Plan-Apochromat 20×/0.75 NA objective, AxioCam HR digital camera, and temperature-controlled environmental chamber at 37°C. The number of adherent HoxB8-derived neutrophils was analyzed in 18 different field of views within the flow chamber at five different time points.

To study the detachment of Hoxb8 derived neutrophils under flow condition with either treatment, cells were seeded into flow chambers. After 10 min of incubation at 37°C, shear stress was applied and gradually increased from 0.2 to 8.0 dyne/cm^2 every 90 s. Time-lapse videos were recorded for 9 min, and relative adhesion was calculated for

seven time points as the percentage of adherent cells relative to initially adherent cells at 0.2 dyne/cm^2 (100%). Analysis and quantification of adherent cells was conducted with Fiji ImageJ.

Murine LPS model

Female Bl6 mice aged 10-12 weeks were i.p. injected with PBS or varying concentrations of *Escherichia coli*-derived lipopolysaccharide (0.1 – 10 mg/kg BW LPS, O111:B4; Sigma). Mice were clinically scored at least once hourly, and longitudinal blood draws were sampled hourly per fraggling of facial veins to assess differential expression of surface markers over time. After 4-6 hours, or when surpassing a predefined scoring threshold, mice were sacrificed. Blood samples were analysed through automated cell counting and flow cytometry on a BD LSRFortessa. For assessing dose-dependent effects on CD177 expression and neutrophil recruitment to inflamed organs, mice were sacrificed and blood, liver, spleen and bone marrow were sampled after two hours. Organs were digested, and all tissue and blood samples were stained with antibodies for flow cytometry-based assessment of neutrophil infiltration and neutrophil surface expression.

Murine *E. coli* bacteremia model

E. coli (DH5alpa-GFP with ampicillin resistance) were cultured to the late log phase in LB-medium (containing 100 µg/ml ampicillin) and optical density was measured to estimate the bacterial number. Female Bl6 mice aged 12-14 weeks were i.v. injected with 2-4 mg/kg BW of isotype (IgG2a) or 2-4 µg of a monoclonal CD177-blocking antibody (MAB8186, BioTechne) and subsequently exposed to 3x 10^8 eGFP-expressing live *E. coli*. Mice were clinically scored. After 6 hours, mice were anesthetized, and blood was collected through cardiac puncture, followed by cardiac perfusion with 10 ml ice-cold PBS. Subsequently, organs were collected for analysis of colony-forming units (CFUs), flow cytometry and histopathological analysis. Colony forming units (CFUs) were determined by mechanically homogenizing small pieces of the liver, spleen and lung using glass beads and plating 10-fold serial dilutions of bacteria on LB plates followed by incubation at 37°C overnight. CFUs were calculated per gram of organ weight. Plasma cytokines were measured using the LEGENDplex™ Mouse Inflammation Panel (13-plex) with V-bottom Plate (Biolegend, #740446) according to the manufacturers' instructions on a BD LSRFortessa flow cytometer.

Adoptive transfer of murine neutrophils.

Peripheral blood and bone marrow neutrophils were isolated as described above. $5x10^6$ neutrophils were then stained using CellTracker Red or FarRed dyes (ThermoFisher, final conc. 10 µM) according to the manufacturer's instructions. Prior to transfusion, some neutrophils were treated with either vehicle (DMSO 10% in PBS) or 1 µg/ml Actinomycin D for 15 min at RT to block mRNA transcription. Cells were then spun down, pooled in NaCl 0.9%. Per recipient mouse, a total of $10x10^6$ neutrophils in 200 µl sterile NaCl 0.9% were slowly injected via tail vein injection. Hereafter, some recipient mice were injected with NaCl 0.9% i.p. or LPS i.p. (1 mg/kg BW). After 6 hours, blood was collected by fraggling and organs were collected for flow cytometric analysis. Stained neutrophils in peripheral blood and in organs were detected using flow cytometry (see Fig. S11A, D for gating scheme).

Sepsis scoring

In both sepsis and bacteremia models, mice were clinically scored. Scoring items included the following: overall appearance, activity, response to external stimuli (both acoustic and tactile), mouse grimace scale(*106*), respiratory rate, and respiration quality. The scoring system was approved by the federal regulatory agency (Regierung Oberbayern).

Immunofluorescence (IF) staining

Immunofluorescence was performed as described previously(*107, 108*). In brief, organs fixed with 4% PFA (1 hour at RT) and dehydrated with 30% sucrose (4°C overnight) were cryoembedded in TissueTek. Organ slices (10 µm) were fixated again using 4% PFA (methylene-free) and subsequently permeabilized and blocked (10% goat serum, 0,5% saponin and 1% BSA in PBS). Samples were stained using fluorescently labelled primary antibodies against neutrophils (Ly6G, clone 1A8), platelets and macrophages (see Suppl. Table 3). Nuclei were counterstained using Hoechst dye (1:1000). Samples were then imaged on a Zeiss LSM 880 confocal microscope at 1-2x magnification in Airyscan Super Resolution (SR) mode (20x/0.8 objective).

Confocal live microscopy of liver

Live imaging of liver was performed as described previously(*109*). In brief, awake mice were i.v. injected with either IgG2a or an anti-CD177 antibody (1 mg/kg BW), followed by injection of AF488-conjugated *E. coli* bioparticles (3x 10^7/ml). After 30 minutes, mice were anesthetized, fluorescent antibodies against Ly6G and F4/80 were i.v. injected and a laparotomy was performed. Per experimental animal, the liver was exteriorized, immobilized to avoid respiration-associated artifacts, and imaged on an inverted Zeiss LSM 880 confocal microscope (20x objective). Longitudinal Z-stacks and single-plane videos were acquired to assess neutrophil infiltration, motility, and intra-organ behavior. Imaris (Bitplane) was used for 3D-reconstruction and track visualization as well as analysis of colocalization (shortest distance calculation), velocities and meandering indices.

Western blot

Per biological replicate (n=4), 300,000 murine bone marrow neutrophils were incubated at 37°C in the presence of either isotype (rat IgG2a) or the CD177-blocking antibody MAB8186. After 2 h, neutrophils were washed once and snap-frozen using liquid nitrogen. The resulting pellets were lysed using RIPA Lysis and 220 Extraction Buffer (ThermoFisher) in the presence of proteinase and phosphatase inhibitors. Protein content was then quantified as previously described, and equal amounts of protein were loaded per lane. Total ACTB, c-Src levels and phospho-Src were identified using primary antibodies and visualized using horse radish peroxidase (HRP)-coupled secondary antibodies and chemiluminescent agents. Densitometry was performed using the Fiji ImageJ gel analysis tool as previously described(*110*).

Visualization

Representative micrographs of *in vitro* experiments and *ex vivo* immunofluorescence studies were imported using Fiji ImageJ. Individual graphs were generated using Seurat and GraphPad Prism v9 and v10. Figures were designed using Adobe Illustrator v9. Experimental schemes and the graphical abstract were generated using BioRender.

Statistical analyses

In experiments involving human donors, peripheral blood neutrophils from at least four individuals were used and considered as biological replicates. For *in vivo* experiments,

at least n=3-4 animals per experimental group were used. Unless otherwise stated, we used unpaired, two-tailed Student's t-tests whenever two groups were compared, and one-way analysis of variance (ANOVA) if three or more groups were part of the experiment; dependent on normality distribution, we used post-hoc Dunn's tests or performed Kruskal-Wallis testing. In grouped analyses, two-way ANOVA testing was applied. Generally, a p-value of <0.05 was considered statistically significant. We marked p-values by asterisks as follows: * <0.05, ** <0.01, *** <0.005, **** <0.001, ns = non-significant. If no asterisks are indicated, no statistical difference between treatment groups was detected.

Suppl. Tables

Table S1: Clinical information on study cohorts

Cohort	Mean age ± SD	Female [%]	Mean SOFA score	WBC [G/l]	CRP [mg/dl]	PCT [ng/ml]	IL-6 [ng/ml]
Control	39.3 ± 9	50	N/A	N/A	N/A	N/A	N/A
Acute bacterial infection	68.8 ± 18	25.9	2.9 ± 2.2	14.4 ± 5	18.2 ± 12	16.1 ± 22	1102 ± 3386
Ischemic stroke	82.4 ± 9	80	1.3 ± 1.5	11.9 ± 2.4	1.54 ± 2.4	N/A	N/A

Table S2: Hashing and CITEseq antibodies

Manufacturer	Order #	Antibody	Clone
Biolegend	394631	TotalSeq™-B0251 anti-human Hashtag 1	LNH-94; 2M2
Biolegend	394633	TotalSeq™-B0252 anti-human Hashtag 2	LNH-94; 2M2
Biolegend	394635	TotalSeq-B0253 anti-human Hashtag 3	LNH-94; 2M2
Biolegend	394637	TotalSeq-B0254 anti-human Hashtag 4	LNH-94; 2M2
Biolegend	394639	TotalSeq-B0255 anti-human Hashtag 5	LNH-94; 2M2
Biolegend	394641	TotalSeq-B0256 anti-human Hashtag 6	LNH-94; 2M2
Biolegend	394643	TotalSeq-B0257 anti-human Hashtag 7	LNH-94; 2M2
Biolegend	394645	TotalSeq-B0258 anti-human Hashtag 8	LNH-94; 2M2
Biolegend	394647	TotalSeq™-B0259 anti-human Hashtag 9	LNH-94; 2M2
Biolegend	344853	TotalSeq-B 0049 anti-human CD3	SK7
Biolegend	300565	TotalSeq-B 0072 anti-human CD4	RPA-T4
Biolegend	347611	TotalSeq-B 1050 anti-human Siglec-10	5G6
Biolegend	303747	TotalSeq-B 0353 anti-human CD41	HIP8
Biolegend	312235	TotalSeq-B 0062 anti-human CD10	HI10a
Biolegend	362561	TotalSeq-B 0047 anti-human CD56 (NCAM)	5.1H11

Biolegend	323051	TotalSeq-B 0392 anti-human CD15 (SSEA-1)	W6D3
Biolegend	302063	TotalSeq-B 0083 anti-human CD16	3G8
Biolegend	367145	TotalSeq-B 0051 anti-human CD14	63D3
Biolegend	302263	TotalSeq-B 0050 anti-human CD19	HIB19

Table S3: Antibodies and fluorescent reagents

Protein/epitope	Fluorophore	Target species	Manufacturer	Order #
anti-mouse IgG	Cy3	mouse	Invitrogen	A10521
anti-rabbit IgG	AF546	rabbit	Invitrogen	A11037
anti-rabbit IgG	AF649	rabbit	Invitrogen	A21244
anti-rat IgG	AF488	rat	Invitrogen	A21208
anti-rat IgG	AF546	rat	Invitrogen	A11007
CD45	FITC	human	Biolegend	304006
CD45	BV650	human	Biolegend	304044
CD15	APC	human	Biolegend	301908
CD15	PE-Cy7	human	Biolegend	323030
CD16	BV510	human	Biolegend	302048
CD182	PE-Dazzle	human	Biolegend	320722
CD184	CD421	human	Biolegend	306518
CD11b	PE-Cy7	human	Biolegend	101216
CD11b	BV711	human	Biolegend	101242
CD10	BV605	human	Biolegend	312222
CD42c	DyLight-488	mouse	emfret	X488
CD42c	DyLight-649	mouse	emfret	X649
CD45	PerCp-Cy5.5	mouse	Biolegend	103132
CD45	BV650	human	Biolegend	304044

CD62L	BV785	human	Biolegend	304830
F4/80	AF647	Mouse	Invitrogen	MF48021
Hoechst Dye	-	-	ThermoFisher	H3570
Ly6G	PE	mouse	Biolegend	127608
Ly6G	BV711	mouse	Biolegend	127643
Ly6G (UltraLeaf)	-	mouse	Biolegend	127649
TER119	PE	mouse	Biolegend	116208
TER119	AF488	mouse	Biolegend	116215
S100A8/9	APC	human	Milteny Biotec	130-115-255
CD114 (GCSFR)	PerCp-Cy5.5	human	Biolegend	315810
IL1R2	PE	human	BioTechne	FAB663P
IFITM2/3	CoraLite AF488	human	Proteintech	CL488-66081
CD88	AF700	human	Biolegend	344314
2′,7′-Dichlorfluorescein-Diacetat	2′,7′-Dichlorfluorescein	-	Sigma	D6883
pHrodo™ Green E. coli BioParticles™	pHrodo Green	-	ThermoFisher	P35366
Escherichia coli BioParticles,	AF488	-	ThermoFisher,	E13231
Fluo-4, AM	Fluo-4	-	ThermoFisher,	F14201

Table S4: Gene lists used for score calculation

**Azurophilic granule score**	**Gelatinase granule score**	**Secretory vesicle score**	**Specific granuly score**	**ISG**	**RPS**
BPI	CTSD	FCGR1A	CST7	MX1	RPS10
LAMP1	MMP2	ALPL	HPSE	MX2	RPS11

PSEN1	TNFAIP6	CCR1	MMP2	IFIT1	RPS12
VNN1	MMP8	LNPEP	SERPINA1	IFIT2	RPS13
AZU1	TNFRSF1A	TLR8	CST3	IFIT3	RPS14
HEXB	B2M	CD14	SLPI	XAF1	RPS15
ARG1	GPI	TLR4	TNFRSF1A	IFI16	RPS15A
FUCA2	CR1	ITM2B	CLEC5A	IFI27	RPS16
GNS	ARSB	TGFBR2	CEACAM1	IFI27L1	RPS17
GLB1	SIRPB1	AGTRAP	B2M	IFI27L2	RPS18
ORM2	ATP11A	TNFRSF1A	ITGAL	IFI30	RPS19
CEACAM6	FCER1G	IFNGR1	GGH	IFI35	RPS19BP1
ACPP	FGL2	TLR1	C3AR1	IFI44	RPS2
GALNS	VAMP8	IGF2R	PTX3	IFI44L	RPS20
MAN2B1	ARG1	MYD88	VAMP2	IFITM1	RPS21
HEXA	CD58	TLR2	STOM	IFITM2	RPS23
SDCBP	FCN1	SYNGR2	CYBA	IFITM3	RPS24
CD63	FCAR	SORL1	SNAP23	IRF9	RPS25
ELANE	ITGAX	CR1	HP	IRF2BP1	RPS26
MPO	MGAM	FCGR3B	PGLYRP1	ISG15	RPS27
GLA	VAMP2	TNFRSF1B	TSPAN14	ISG20	RPS27A
CTSG	CYBA	IFNAR2	CD59	IRF2BP2	RPS27L
PRTN3	SCAMP2	ANTXR2	FOLR3	IRF1	RPS28
GUSB	TNFSF14	DYSF	PLAU	IRF3	RPS29
LYZ	MMP25	TLR6	PLAUR	IRF2BPL	RPS3
	VNN2	FCAR	CYBB	LY6E	RPS3A
	LILRB2	VAMP2	ITGB2	IFIT1	RPS4X

	SIGLEC5	CYBA	ITGAM	IFIT1B	RPS4Y1
	TIMP2	MMP25	TCN1	IFIT2	RPS4Y2
	SNAP23	LILRB2	CEACAM8	IFIT3	RPS5
	TCIRG1	SNAP23	ORM1	IFIT5	RPS6
	PRSS3	IL6R	OLFM4	RSAD2	RPS7
	ADAM8	FCGRT	LYZ	IRF1	RPS8
	MMP9	PLXNC1	LTF	IRF2	RPS9
	ASAH1	FPR1	CHI3L1	IRF3	RPSA
	PRCP	SIGLEC9	CLCN2	IRF4	RACK1
	CTSB	P2RX1	CRISP3	IRF5	
	PLAUR	PLP2	CAMP	IRF7	
	CD33	FCGR3A	CD177	IRF8	
	SLC11A1	LILRB3		IRF9	
	CYBB	CYBB		MT2A	
	LAMP2	ANPEP			
	ITGB2	CXCR1			
	CTSH	ITGB2			
	ITGAM	ITGAM			
	TCN1	TREM1			
	CEACAM8	CXCR2			
	ORM1	MME			
	CTSZ				
	CTSS				
	LYZ				
	CRISP3				
	CAMP				

(continued)

RPL score	NET formation score	Complement score	Transmigration score	Neutrophil extravasation score
RPL10	Ela2	C1R	ITGAM	ADAM8
RPL10A	Ltf	C1S	ITGAX	CD99
RPL11	Azu1	C9	SELPGL	CD99L2
RPL12	Ctsg	C1QC	SELL	IL1A
RPL13	Mpo	C3	CD177	IL1R1
RPL13A	Pr3	C4	CD99	MDK
RPL14	Lyz	C5	VLA3	PAWR
RPL15	Defa1	C6	VLA6	PTGER3
RPL17	Defa3	C7	CD47	PTGER4
RPL18	H2a	C8	CXCL8	RIPOR2
RPL18A	H2b	C9	PAFR	CD47
RPL19	H3	CD55	CD49	FADD
RPL21	H4	C1QB		ICAM1
RPL22	Mnda	C3AR1		LYVE1
RPL22L1	S100a8	C5AR1		MED23
RPL23	S100a9	CFD		PAWR
RPL23A	S100a12	CFB		PDGFD
RPL24	Actb	CFP		PECAM1
RPL26	Actg1	CR1		PLVAP
RPL26L1	Myh9	C4BPA		PTAFR
RPL27	Actn1	CFH		
RPL27A	Actn4			
RPL28	Lcp1			
RPL29	Krt10			
RPL3	Cat			

RPL30	Eno1			
RPL31	Tkt			
RPL32	PADI4			
RPL34				
RPL35				
RPL35A				
RPL36				
RPL36A				
RPL36AL				
RPL37				
RPL37A				
RPL38				
RPL39				
RPL39L				
RPL3L				
RPL4				
RPL41				
RPL5				
RPL6				
RPL7				
RPL7A				
RPL7L1				
RPL8				
RPL9				
RPLP0				
RPLP1				
RPLP2				

Table S5: FlowSet gene sets (adj. p-value < 0.05)

For visualization, see Fig. 3F-H.

	pwid	pwname	pwFlow	pwGenes	pval	adj_pval
6081	GO:0002181	cytoplasmic translation	4.547339	89	7.67E-32	1.07E-28
5410	GO:0002523	leukocyte migration involved in inflammatory response	0.570682	12	1.33E-20	8.32E-18
5963	GO:0035425	autocrine signaling	0.527943	7	1.07E-19	5.84E-17
75	GO:0006412	translation	4.674773	174	1.6E-16	8.02E-14
5513	GO:0042270	protection from natural killer cell mediated cytotoxicity	0.390157	6	6.7E-15	3E-12
936	GO:0014002	astrocyte development	0.641372	16	9.25E-15	3.86E-12
3432	GO:0002544	chronic inflammatory response	0.542368	9	6.04E-13	2.36E-10
2460	GO:0035456	response to interferon-beta	0.403829	11	1.81E-12	5.98E-10
7070	GO:0035455	response to interferon-alpha	0.398078	11	3.93E-12	1.23E-09
5510	GO:0002486	antigen processing and presentation of endogenous peptide antigen via MHC class I via ER pathway, TAP-independent	0.571516	16	7.79E-12	2.22E-09
7114	GO:0002476	antigen processing and presentation of endogenous peptide antigen via MHC class Ib	0.571516	16	7.79E-12	2.22E-09
5527	GO:0015670	carbon dioxide transport	0.464077	13	8.04E-12	2.24E-09
5813	GO:0042590	antigen processing and presentation of exogenous peptide antigen via MHC class I	0.327531	6	8.16E-11	2.09E-08
5685	GO:0006880	intracellular sequestering of iron ion	0.320097	6	2.23E-10	5.37E-08

63<br>27	GO:0045<br>646	regulation of erythrocyte differentiation	0.3198<br>41	6	2.31E-<br>10	5.45E-<br>08
25<br>70	GO:0050<br>832	defense response to fungus	1.0025<br>63	31	8.11E-<br>10	1.85E-<br>07
29<br>95	GO:0002<br>726	positive regulation of T cell cytokine production	0.3452<br>22	11	2.76E-<br>09	5.78E-<br>07
74	GO:0000<br>028	ribosomal small subunit assembly	0.4993<br>54	16	3.44E-<br>09	7.07E-<br>07
52<br>50	GO:0015<br>671	oxygen transport	0.4672<br>19	15	3.72E-<br>09	7.52E-<br>07
68<br>11	GO:0045<br>576	mast cell activation	0.3320<br>69	7	1.95E-<br>08	3.64E-<br>06
48<br>37	GO:1904<br>667	negative regulation of ubiquitin protein ligase activity	0.3437<br>18	12	7.44E-<br>08	1.3E-05
38<br>75	GO:0098<br>974	postsynaptic actin cytoskeleton organization	0.3140<br>65	11	8.26E-<br>08	1.42E-<br>05
45<br>79	GO:0006<br>596	polyamine biosynthetic process	0.2635<br>48	6	2.15E-<br>07	3.51E-<br>05
56<br>64	GO:0006<br>826	iron ion transport	0.5756<br>16	21	2.95E-<br>07	4.62E-<br>05
58<br>79	GO:0045<br>730	respiratory burst	0.4347<br>57	16	3.81E-<br>07	5.9E-05
22<br>0	GO:0006<br>801	superoxide metabolic process	0.4786<br>65	18	7.08E-<br>07	0.00010<br>3
41<br>62	GO:0016<br>045	detection of bacterium	0.3163<br>35	12	9.03E-<br>07	0.00012<br>9
31<br>83	GO:1900<br>242	regulation of synaptic vesicle endocytosis	0.3390<br>11	13	1.21E-<br>06	0.00016<br>2
64<br>71	GO:0042<br>274	ribosomal small subunit biogenesis	0.5468<br>86	21	1.26E-<br>06	0.00016<br>6
28<br>99	GO:0051<br>493	regulation of cytoskeleton organization	0.5924<br>61	23	1.68E-<br>06	0.00021<br>3
28<br>54	GO:0019<br>885	antigen processing and presentation of endogenous peptide antigen via MHC class I	0.3239<br>59	8	1.88E-<br>06	0.00023<br>3
26<br>87	GO:0001<br>916	positive regulation of T cell mediated cytotoxicity	0.9145<br>18	36	2.41E-<br>06	0.00029<br>6

7983	GO:0002283	neutrophil activation involved in immune response	0.320319	8	2.47E-06	0.000299
2373	GO:0034333	adherens junction assembly	0.299982	12	3.6E-06	0.000415
4206	GO:2001171	positive regulation of ATP biosynthetic process	0.318622	13	5.81E-06	0.000651
5159	GO:0055093	response to hyperoxia	0.364876	15	6.94E-06	0.00077
1336	GO:0006123	mitochondrial electron transport, cytochrome c to oxygen	0.580322	24	7.97E-06	0.000869
5386	GO:0022898	regulation of transmembrane transporter activity	0.265978	7	8.12E-06	0.000877
3414	GO:0032930	positive regulation of superoxide anion generation	0.44986	19	1.28E-05	0.001333
7120	GO:0051764	actin crosslink formation	0.331395	14	1.29E-05	0.001333
5780	GO:0032364	cellular oxygen homeostasis	0.257397	7	1.61E-05	0.001627
1663	GO:0070293	renal absorption	0.32162	9	2.82E-05	0.002656
1375	GO:0042542	response to hydrogen peroxide	0.906176	40	3.24E-05	0.003034
35	GO:0030593	neutrophil chemotaxis	1.602184	81	4.31E-05	0.003889
9644	GO:0140058	neuron projection arborization	0.208864	6	4.59E-05	0.004106
6733	GO:0140507	granzyme-mediated programmed cell death signaling pathway	0.202504	6	7.89E-05	0.00668
2209	GO:0045744	negative regulation of G protein-coupled receptor signaling pathway	0.278896	13	9.13E-05	0.007686
955	GO:0045429	positive regulation of nitric oxide biosynthetic process	0.811658	38	9.88E-05	0.008259
7196	GO:1900122	positive regulation of receptor binding	0.198064	6	0.000114	0.009466
3854	GO:0055072	iron ion homeostasis	0.804676	38	0.000115	0.009492

883	GO:0070527	platelet aggregation	0.963236	46	0.00014	0.011443
4791	GO:0001913	T cell mediated cytotoxicity	0.31095	15	0.000166	0.013221
988	GO:0042776	proton motive force-driven mitochondrial ATP synthesis	1.16591	64	0.000201	0.015722
2025	GO:0043542	endothelial cell migration	0.690022	34	0.000234	0.018132
3679	GO:0051279	regulation of release of sequestered calcium ion into cytosol	0.281762	14	0.000268	0.019964
6241	GO:0031668	cellular response to extracellular stimulus	0.361218	18	0.00028	0.020762
771	GO:0001836	release of cytochrome c from mitochondria	0.440543	22	0.000289	0.021223
5512	GO:0032675	regulation of interleukin-6 production	0.307044	10	0.000341	0.024593
2572	GO:0061844	antimicrobial humoral immune response mediated by antimicrobial peptide	1.160509	66	0.00036	0.025759
3412	GO:0030889	negative regulation of B cell proliferation	0.314706	16	0.000381	0.027114
2487	GO:0034341	response to type II interferon	0.506481	26	0.000439	0.030944
1076	GO:0002237	response to molecule of bacterial origin	0.270693	9	0.000454	0.031778
1275	GO:2001244	positive regulation of intrinsic apoptotic signaling pathway	0.600119	31	0.000481	0.033525
8673	GO:0019941	modification-dependent protein catabolic process	0.268343	9	0.00051	0.03532
5666	GO:0010042	response to manganese ion	0.268042	9	0.000518	0.035652
5016	GO:0042554	superoxide anion generation	0.458928	24	0.000574	0.039119
1337	GO:0045333	cellular respiration	0.757344	40	0.00066	0.04398
6122	GO:0038094	Fc-gamma receptor signaling pathway	0.204487	7	0.000665	0.044133

50 17	GO:0050 665	hydrogen peroxide biosynthetic process	0.2913 7	10	0.000 688	0.04536 7
15 78	GO:1905 461	positive regulation of vascular associated smooth muscle cell apoptotic process	0.2028 26	7	0.000 738	0.04841 3

Fig. S1

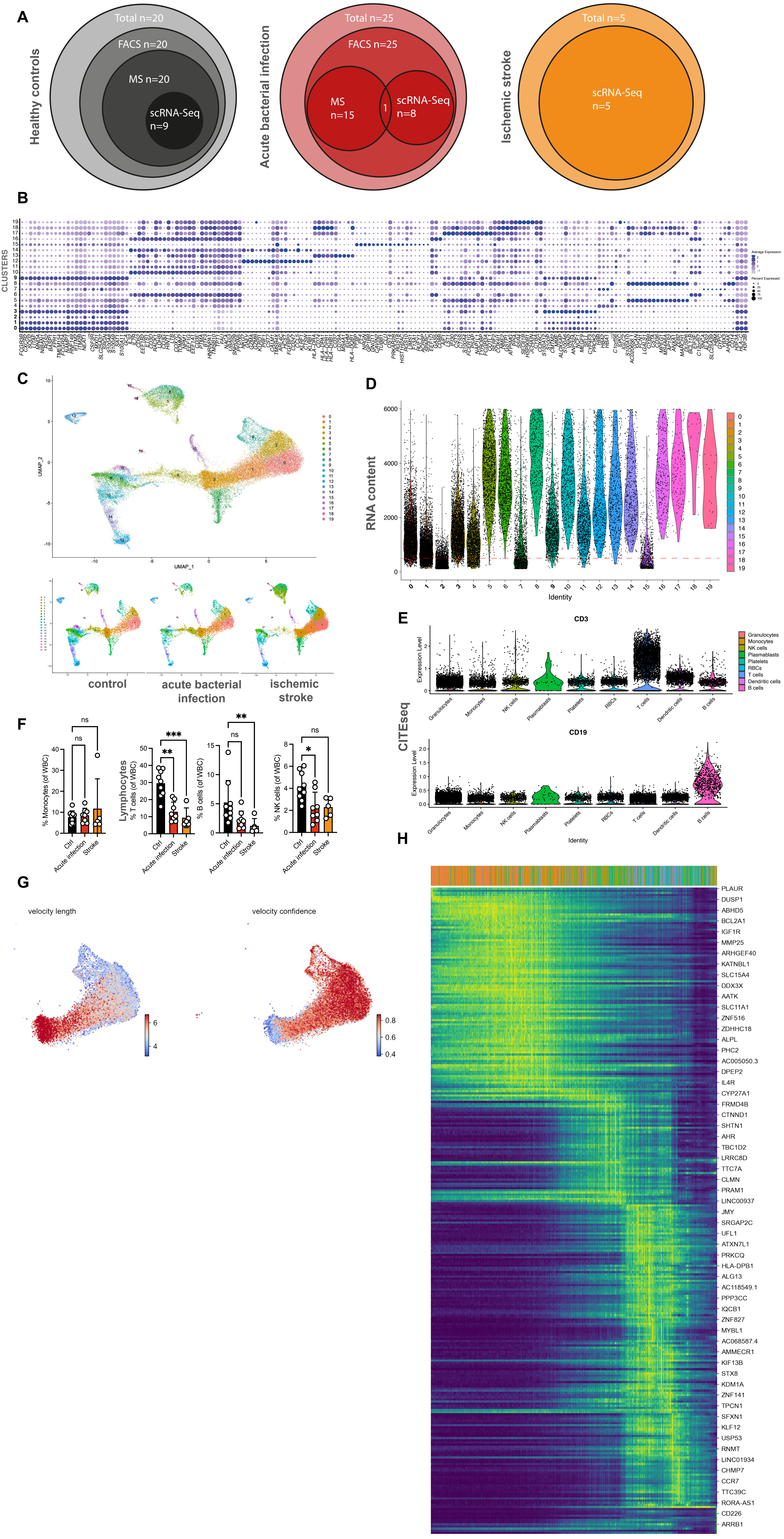
A
Healthy controls
Total n=20
FACS n=20
MS n=20
scRNA-Seq n=9
Acute bacterial infection
Total n=25
FACS n=25
MS n=15
1
scRNA-Seq n=8
Ischemic stroke
Total n=5
scRNA-Seq n=5
B
CLUSTERS
Average Expression
Percent Expressed
C
UMAP_2
UMAP_1
control
acute bacterial infection
ischemic stroke
D
RNA content
Identity
E
CD3
CD19
Expression Level
Granulocytes
Monocytes
NK cells
Plasmablasts
Platelets
RBCs
T cells
Dendritic cells
B cells
CITEseq
Identity
F
% Monocytes (of WBC)
Lymphocytes % T cells (of WBC)
% B cells (of WBC)
% NK cells (of WBC)
ns
**
***
*
Ctrl
Acute infection
Stroke
G
velocity length
velocity confidence
H
PLAUR
DUSP1
ABHD5
BCL2A1
IGF1R
MMP25
ARHGEF40
KATNBL1
SLC15A4
DDX3X
AATK
SLC11A1
ZNF516
ZDHHC18
ALPL
PHC2
AC005050.3
DPEP2
IL4R
CYP27A1
FRMD4B
CTNND1
SHTN1
AHR
TBC1D2
LRRC8D
TTC7A
CLMN
PRAM1
LINC00937
JMY
SRGAP2C
UFL1
ATXN7L1
PRKCQ
HLA-DPB1
ALG13
AC118549.1
PPP3CC
IQCB1
ZNF827
MYBL1
AC068587.4
AMMECR1
KIF13B
STX8
KDM1A
ZNF141
TPCN1
SFXN1
KLF12
USP53
RNMT
LINC01934
CHMP7
CCR7
TTC39C
RORA-AS1
CD226
ARRB1

Fig. S1. | (A) Modified Venn diagrams showing the total number of samples from different conditions with overlapping inclusion into performed experiments. (B) Dot plot depicting cluster-defining genes of clusters 0-19 used to identify cell types. See Figure 1C for gene expression patterns according to cell type. (C) Integrative UMAP of singe-cell RNA sequencing (scRNAseq) data from human leukocytes, separated according to clusters (upper panel) and conditions (lower panels). (D) Violin plot depicting the RNA counts across leukocyte population for individual clusters 0-19. (E) Violin plots depicting the single-cell surface expression of CD3 and CD19 as assess by CITEseq. (F) Relative quantification of monocyte and lymphocyte numbers for the three experimental groups, derived from scRNAseq data. (G) Representation of velocity length (left) and confidence (right) for pseudotime analyses, related to Fig. 1J, K. (H) Heatmap of the genes having the highest likelihood of explaining latent time w.r.t. the analyses shown in Fig. 1J, K.

Fig. S2

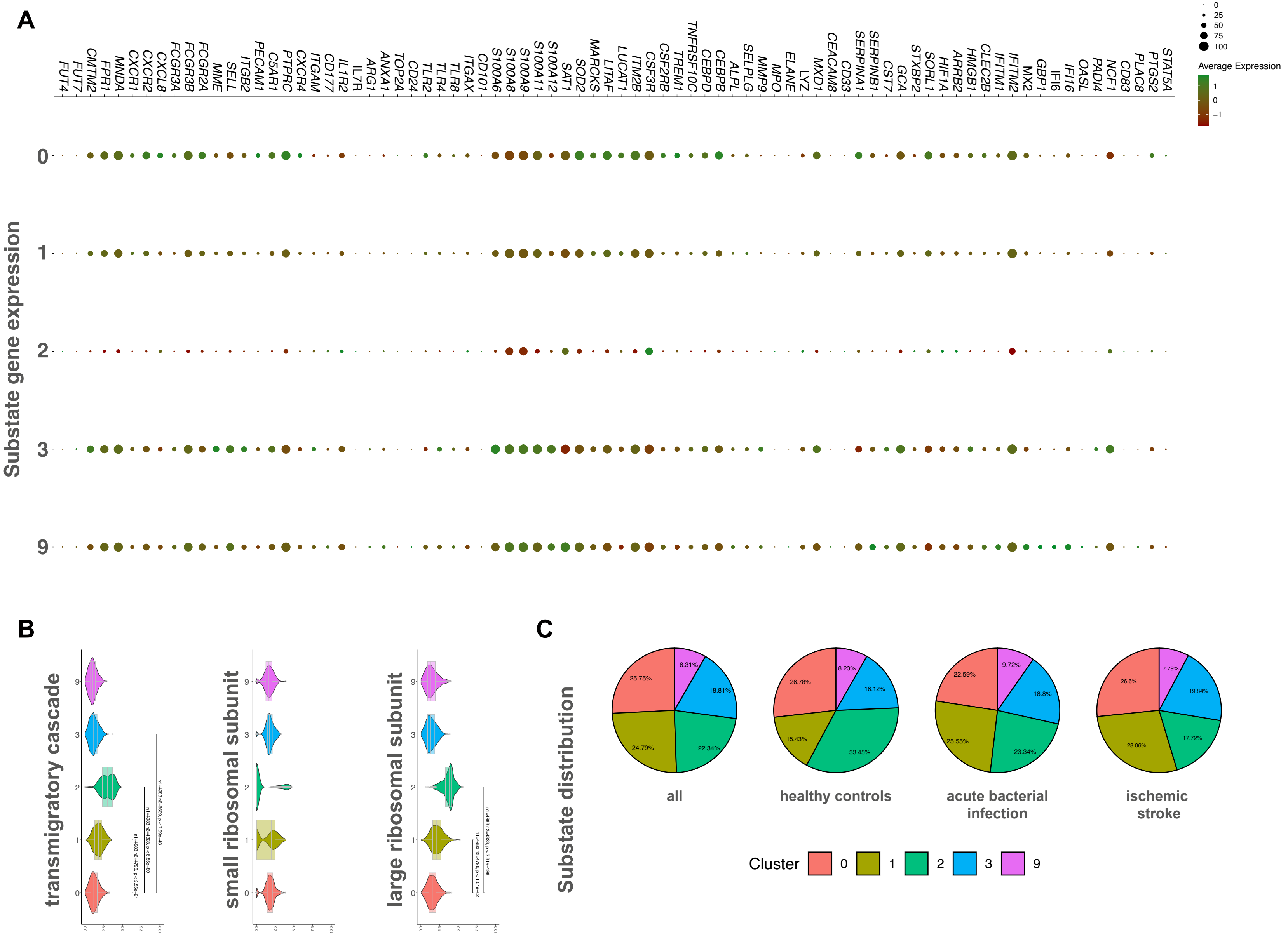
A
Percent Expressed
0
25
50
75
100
Average Expression
1
0
−1
FUT4
FUT7
CMTM2
FPR1
MNDA
CXCR1
CXCR2
CXCL8
FCGR3A
FCGR3B
FCGR2A
MME
SELL
ITGB2
PECAM1
C5AR1
PTPRC
CXCR4
ITGAM
CD177
IL1R2
IL7R
ARG1
ANXA1
TOP2A
CD24
TLR2
TLR4
TLR8
ITGAX
CD101
S100A6
S100A8
S100A9
S100A11
S100A12
SAT1
SOD2
MARCKS
LITAF
LUCAT1
ITM2B
CSF3R
CSF2RB
TREM1
TNFRSF10C
CEBPD
CEBPB
ALPL
SELPLG
MMP9
MPO
ELANE
LYZ
MXD1
CEACAM8
CD33
SERPINA1
SERPINB1
CST7
GCA
STXBP2
SORL1
HIF1A
ARRB2
HMGB1
CLEC2B
IFITM1
IFITM2
MX2
GBP1
IFI6
IFI16
OASL
PADI4
NCF1
CD83
PLAC8
PTGS2
STAT5A
Substate gene expression
0
1
2
3
9
B
transmigratory cascade
small ribosomal subunit
large ribosomal subunit
C
Substate distribution
25.75%
8.31%
18.81%
22.34%
24.79%
all
26.78%
8.23%
16.12%
33.45%
15.43%
healthy controls
22.59%
9.72%
18.8%
23.34%
25.55%
acute bacterial infection
26.6%
7.79%
19.84%
17.72%
28.06%
ischemic stroke
Cluster
0
1
2
3
9

Fig. S2. | (A) Dot plot of genes-of-interest (GOIs) expressed by neutrophil clusters 0, 1, 2, 3, and 9. Color depicts the average expression, dot size represents the percent of the respective cluster expressing the indicated GOI. (B) Violin plots indicating gene sets related to transmigration and ribosomal protein synthesis. (C) Quantification of neutrophil subset distribution across experimental groups as well as for integrated data derived from all groups.

Fig. S3

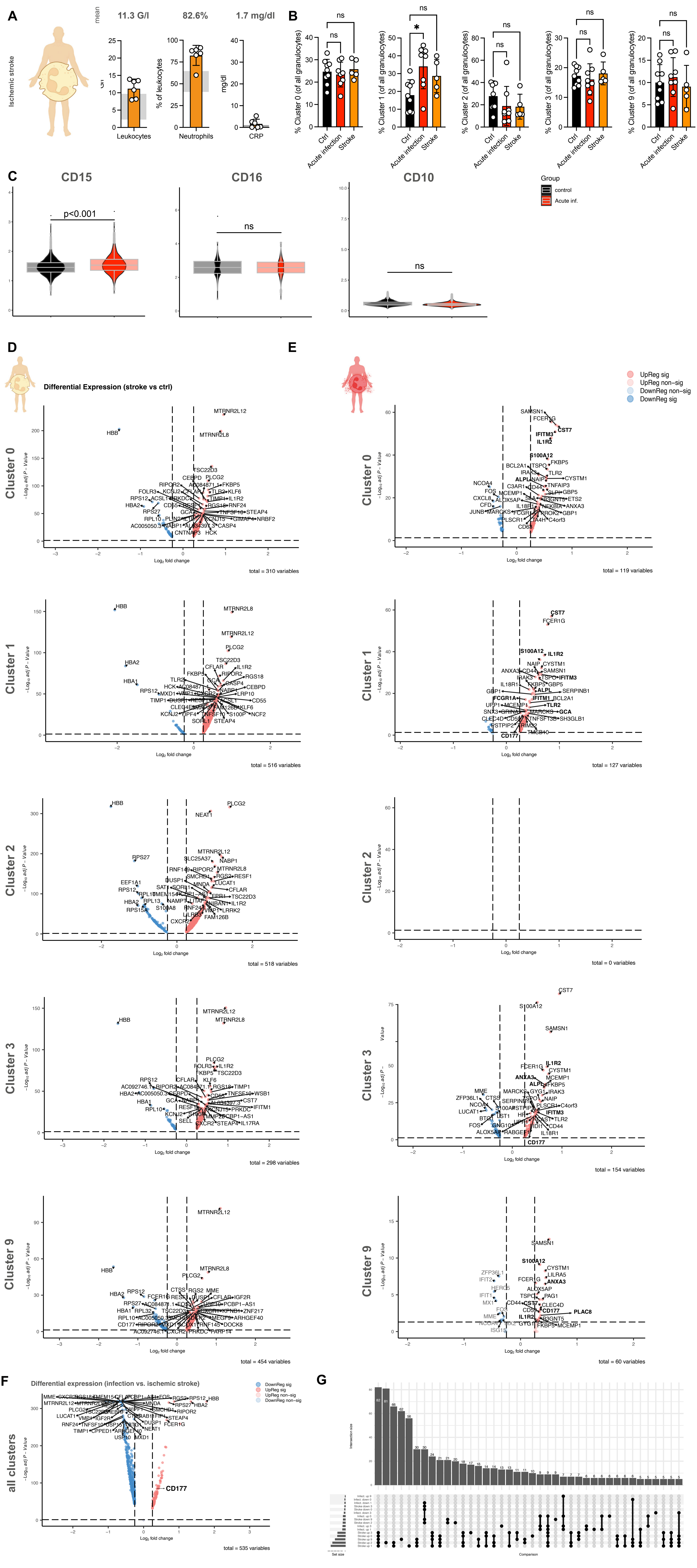

A
Ischemic stroke
mean
11.3 G/l
82.6%
1.7 mg/dl
Leukocytes
Neutrophils
CRP
% of leukocytes
mg/dl
B
ns
*
Ctrl
Acute infection
Stroke
% Cluster 0 (of all granulocytes)
% Cluster 1 (of all granulocytes)
% Cluster 2 (of all granulocytes)
% Cluster 3 (of all granulocytes)
% Cluster 9 (of all granulocytes)
C
CD15
CD16
CD10
p<0.001
Group
control
Acute inf.
D
Differential Expression (stroke vs ctrl)
E
UpReg sig
UpReg non-sig
DownReg non-sig
DownReg sig
Cluster 0
Cluster 1
Cluster 2
Cluster 3
Cluster 9
Log2 fold change
total = 310 variables
total = 119 variables
total = 516 variables
total = 127 variables
total = 518 variables
total = 0 variables
total = 298 variables
total = 154 variables
total = 454 variables
total = 60 variables
F
Differential expression (infection vs. ischemic stroke)
DownReg sig
UpReg sig
UpReg non-sig
DownReg non-sig
all clusters
CD177
total = 535 variables
G
Intersection size
Set size
Comparison

Fig. S3. | (A) Clinical laboratory markers (leukocyte count, relative amount of neutrophils and C-reactive protein levels) from n=5 patients with ischemic stroke from which scRNAseq data were analyzed. Grey boxes indicate the normal range (NR) of the respective parameters, numbers indicate the mean value. (B) Quantification of relative abundancy of neutrophils clusters in control vs. infection vs. ischemic stroke patients. One-way ANOVA with post-hoc Kruskal-Wallis testing. (C) Violin plots depicting the surface expression of CD15, CD16 and CD10/MME as assessed by CITEseq for the indicated patient cohorts. One-way ANOVA with post-hoc Kruskal-Wallis testing. (D, E) Scatter plots depicting substate-specific up- (red) or downregulation (blue) in patients with stroke (yellow, D) or acute infection (red, E). (F) Scatter plot comparing differentially expressed genes of neutrophils from patients with acute bacterial infection compared to patients with ischemic stroke. (G) Upset plot depicting jointly up- or downregulated gene sets across experimental groups.

Fig. S4

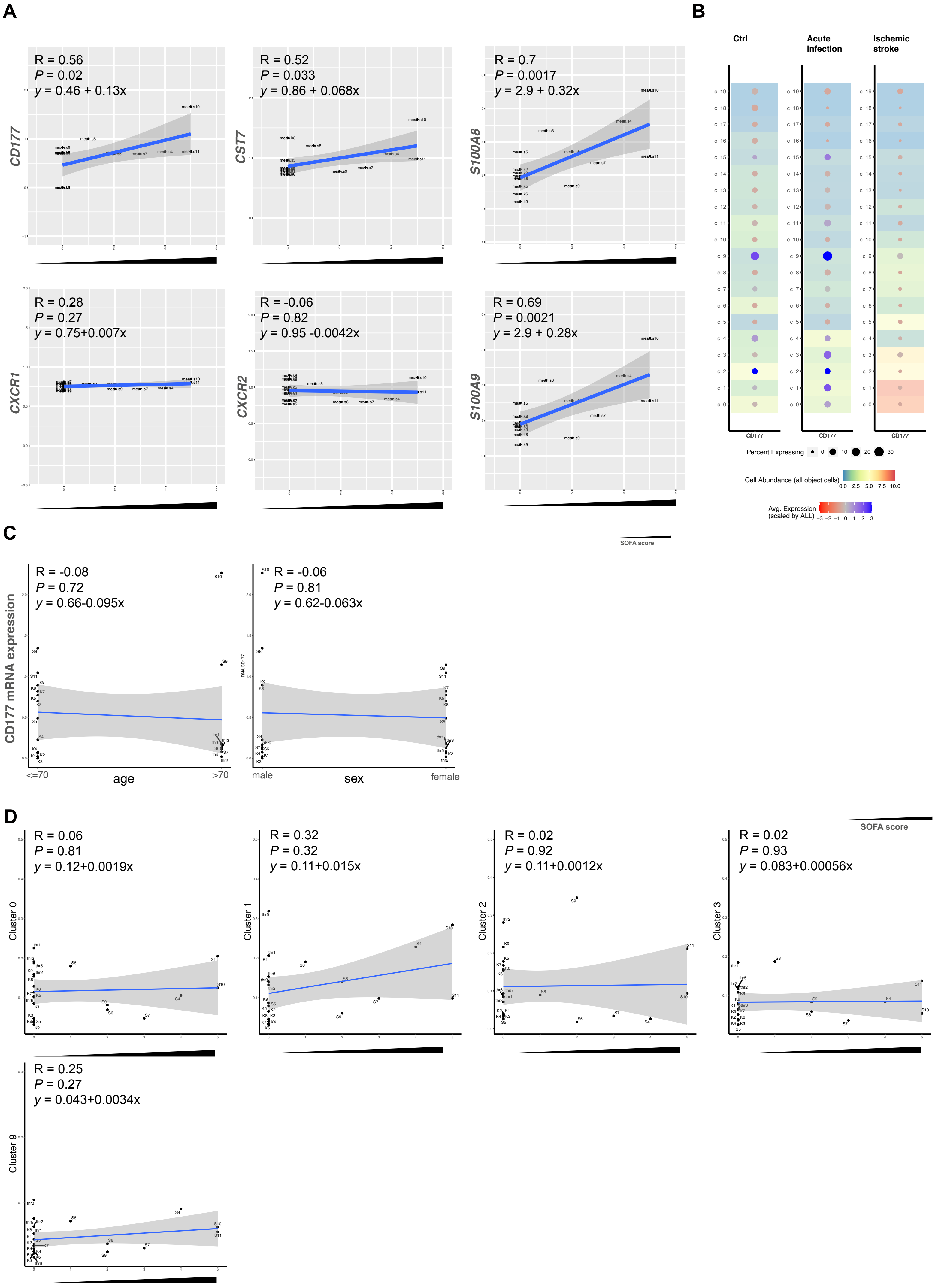
A
R = 0.56
P = 0.02
y = 0.46 + 0.13x
CD177
R = 0.52
P = 0.033
y = 0.86 + 0.068x
CST7
R = 0.7
P = 0.0017
y = 2.9 + 0.32x
S100A8
R = 0.28
P = 0.27
y = 0.75+0.007x
CXCR1
R = -0.06
P = 0.82
y = 0.95 -0.0042x
CXCR2
R = 0.69
P = 0.0021
y = 2.9 + 0.28x
S100A9
SOFA score
B
Ctrl
Acute infection
Ischemic stroke
CD177
Percent Expressing
Cell Abundance (all object cells)
Avg. Expression (scaled by ALL)
C
CD177 mRNA expression
R = -0.08
P = 0.72
y = 0.66-0.095x
<=70
age
>70
R = -0.06
P = 0.81
y = 0.62-0.063x
male
sex
female
D
SOFA score
Cluster 0
R = 0.06
P = 0.81
y = 0.12+0.0019x
Cluster 1
R = 0.32
P = 0.32
y = 0.11+0.015x
Cluster 2
R = 0.02
P = 0.92
y = 0.11+0.0012x
Cluster 3
R = 0.02
P = 0.93
y = 0.083+0.00056x
Cluster 9
R = 0.25
P = 0.27
y = 0.043+0.0034x

Fig. S4. | (A) Linear regression analyses, correlating sequential organ failure assessment (SOFA) score and mean expression levels of the indicated genes. (B) Dot plot depicting cell abundancy and scaled expression of *CD177* transcripts across all leukocyte clusters identified by single-cell RNA sequencing in all indicated experimental groups. (C) Linear regression analyses, correlating *CD177* mRNA expression with sex and age (binarily divided into <70 vs >= 70 years). (D) Linear regression analyses, correlating sequential organ failure assessment (SOFA) score and neutrophil substate abundance for each individual of the substates 0, 1, 2, 3, and 9.

Fig. S5

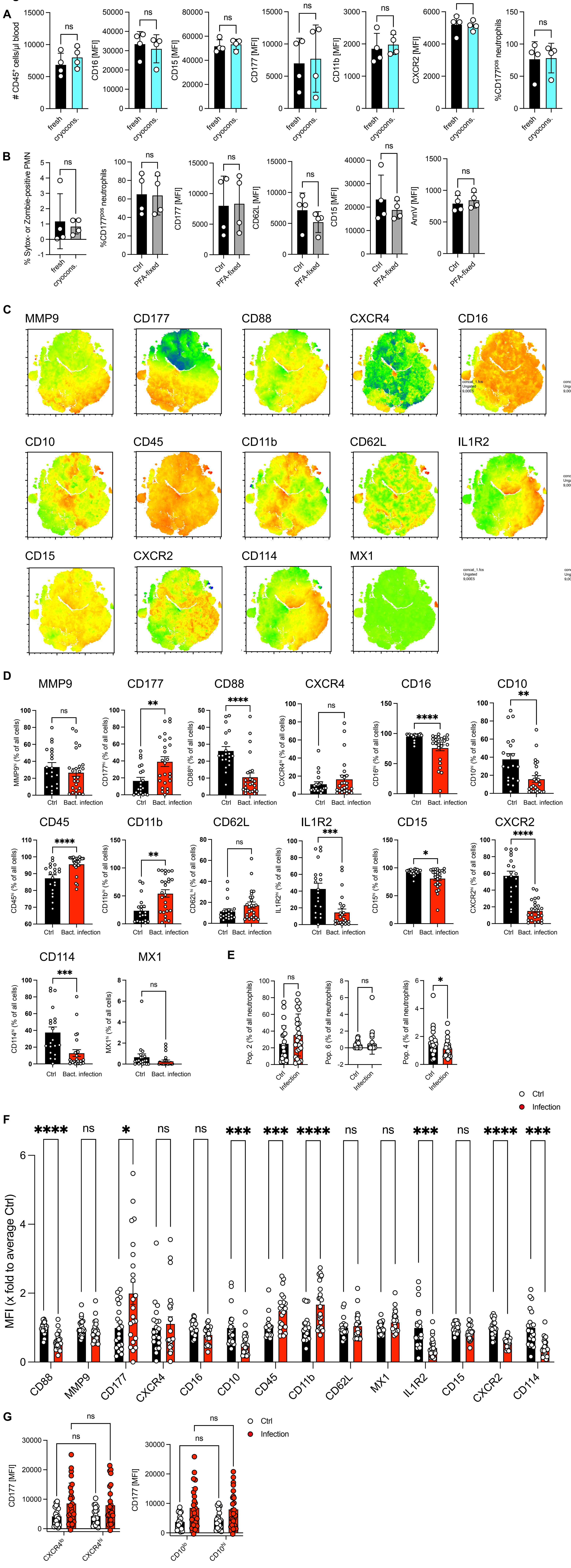

A
CD45+ cells/µl blood
CD16 [MFI]
CD15 [MFI]
CD177 [MFI]
CD11b [MFI]
CXCR2 [MFI]
%CD177pos neutrophils
fresh
cryocons.
ns
B
% Sytox- or Zombie-positive PMN
%CD177pos neutrophils
CD177 [MFI]
CD62L [MFI]
CD15 [MFI]
AnnV [MFI]
Ctrl
PFA-fixed
C
MMP9
CD177
CD88
CXCR4
CD16
CD10
CD45
CD11b
CD62L
IL1R2
CD15
CXCR2
CD114
MX1
D
MMP9
CD177
CD88
CXCR4
CD16
CD10
CD45
CD11b
CD62L
IL1R2
CD15
CXCR2
CD114
MX1
Ctrl
Bact. infection
E
Pop. 2 (% of all neutrophils)
Pop. 6 (% of all neutrophils)
Pop. 4 (% of all neutrophils)
Ctrl
Infection
F
MFI (x fold to average Ctrl)
CD88
MMP9
CD177
CXCR4
CD16
CD10
CD45
CD11b
CD62L
MX1
IL1R2
CD15
CXCR2
CD114
G
CD177 [MFI]
CXCR4lo
CXCR4hi
CD10lo
CD10hi
Ctrl
Infection

Fig. S5. | (A) Quantification of MFIs of the indicated surface markers and of the indicated cell populations in human fixated neutrophils subjected to cryoconservation at -80°C (cyan) or not (black). Student's t-test, two-tailed, unpaired. (B) Quantification of MFIs of the indicated surface markers and cell populations of human live neutrophils (black) vs. PFA-fixed neutrophils (grey). Student's t-test, two-tailed, unpaired. (C) Representative t-Stochastic Neighbour Embedding (t-SNE) plots showing expression of the indicated markers across all measured neutrophils (n=900,000). (D) Quantification of MFIs of the indicated cell surface and intracellular protein markers for healthy controls vs. patients with acute infection. Student's t-test, two-tailed, unpaired. (E) Relative abundancies of populations 2, 4 and 6 in controls vs patients. Student's t-test, unpaired, two-tailed. (F) Normalized MFIs (x fold to average MFI of control group) for the indicated protein markers. Two-way ANOVA with post-hoc Dunnett's testing. (G) Quantification of absolute CD177 MFIs in CD10hi/low and CXCR4hi/low neutrophils. Two-way ANOVA with post-hoc Dunnett's testing.

Fig. S6

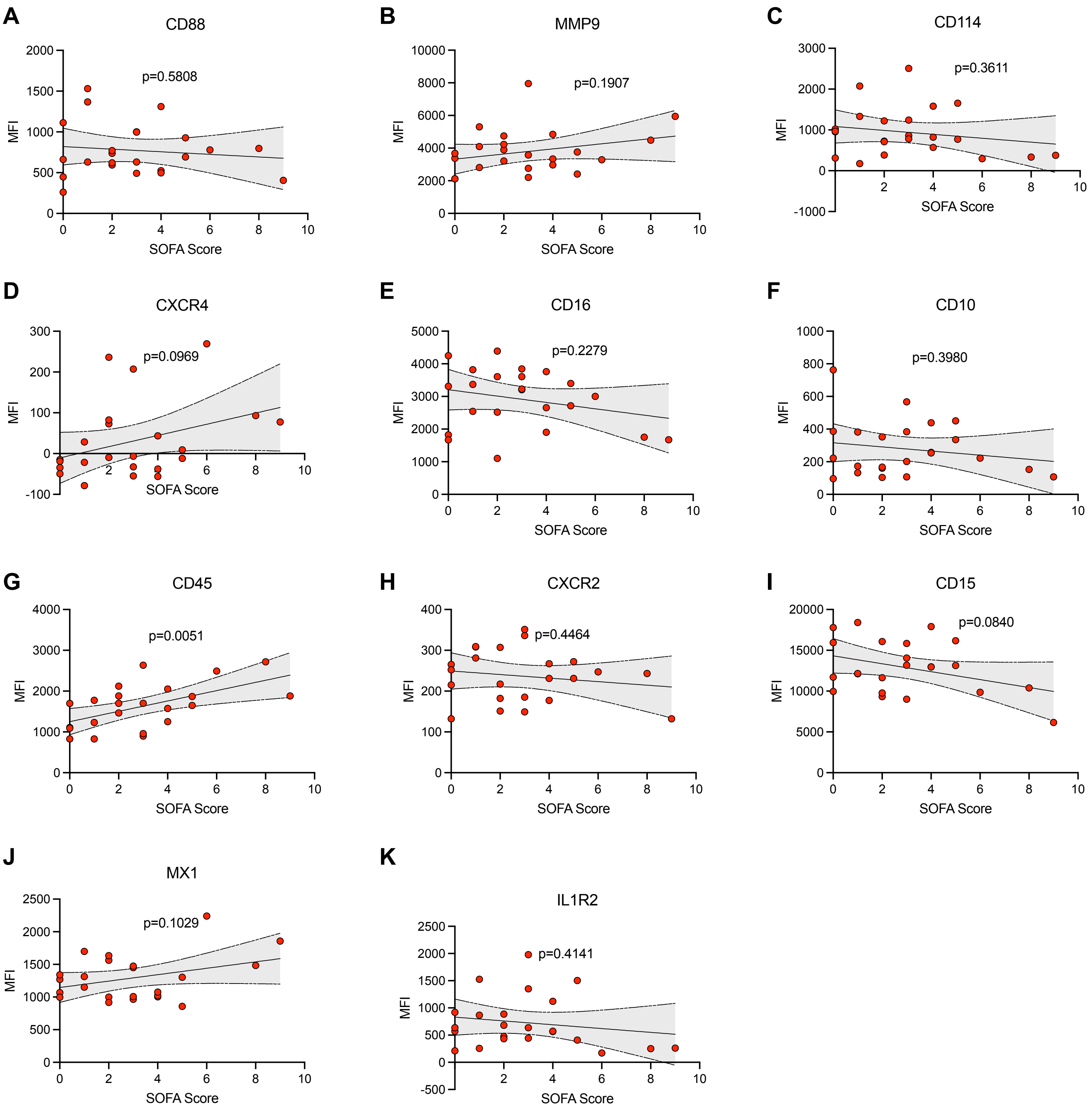
A
CD88
p=0.5808
B
MMP9
p=0.1907
C
CD114
p=0.3611
D
CXCR4
p=0.0969
E
CD16
p=0.2279
F
CD10
p=0.3980
G
CD45
p=0.0051
H
CXCR2
p=0.4464
I
CD15
p=0.0840
J
MX1
p=0.1029
K
IL1R2
p=0.4141
MFI
SOFA Score

Fig. S6. | (A-K) Linear regression analyses, correlating sequential organ failure assessment (SOFA) score and mean fluorescence intensities of the indicated neutrophil surface markers measured in patients with acute bacterial infections. See Fig. 3F for corresponding analysis with CD177, CD11b and L-selectin/CD62L.

Fig. S7

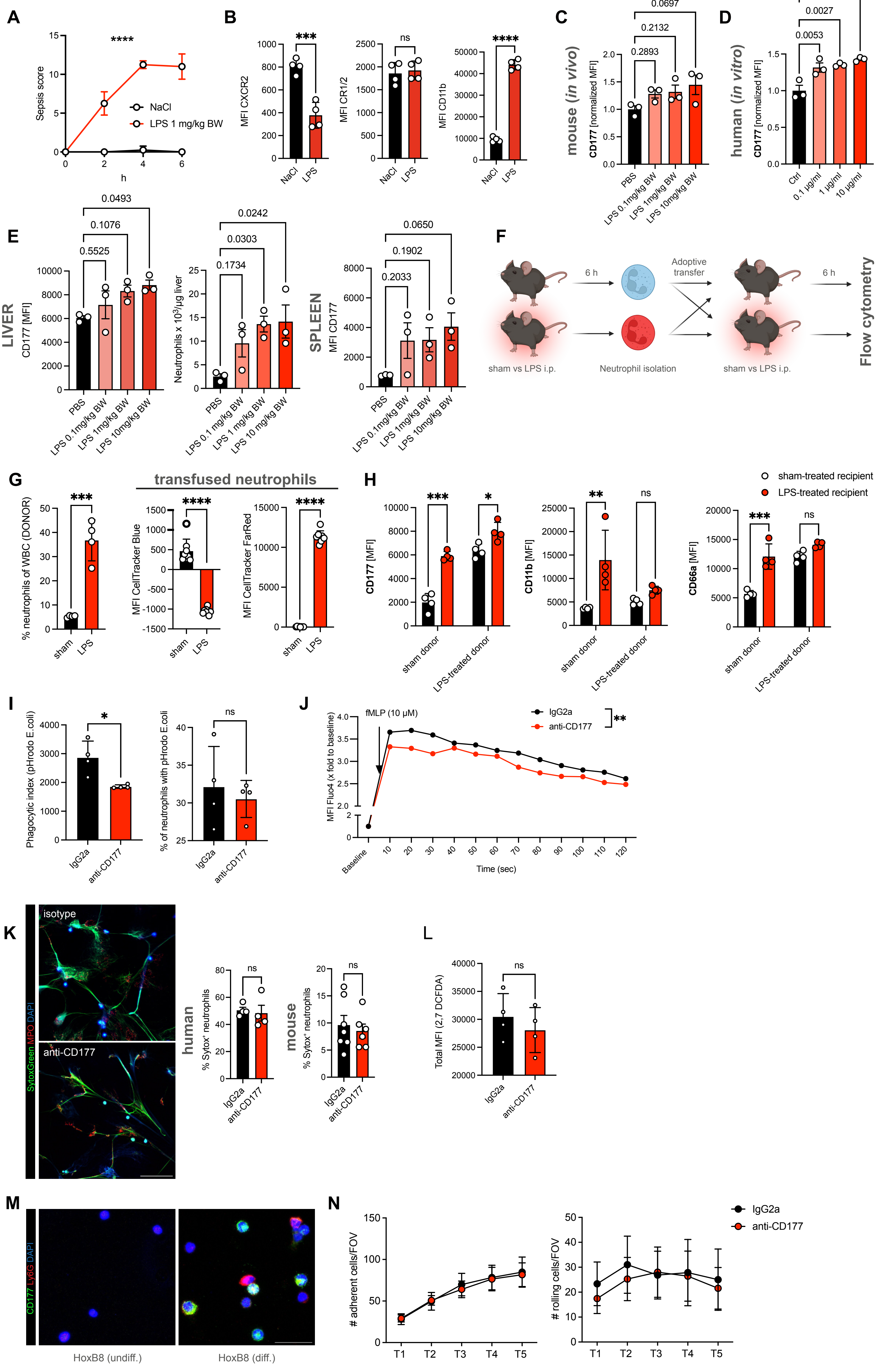
A
Sepsis score
NaCl
LPS 1 mg/kg BW
h
B
MFI CXCR2
MFI CR1/2
MFI CD11b
C
mouse (in vivo)
CD177 [normalized MFI]
D
human (in vitro)
E
LIVER
CD177 [MFI]
Neutrophils x 10³/µg liver
SPLEEN
MFI CD177
F
sham vs LPS i.p.
Neutrophil isolation
Adoptive transfer
Flow cytometry
G
transfused neutrophils
% neutrophils of WBC (DONOR)
MFI CellTracker Blue
MFI CellTracker FarRed
H
sham-treated recipient
LPS-treated recipient
sham donor
LPS-treated donor
I
Phagocytic index (pHrodo E.coli)
% of neutrophils with pHrodo E.coli
J
fMLP (10 µM)
IgG2a
anti-CD177
MFI Fluo4 (x fold to baseline)
Time (sec)
K
isotype
anti-CD177
SytoxGreen MPO DAPI
human
mouse
% Sytox⁺ neutrophils
L
Total MFI (2,7 DCFDA)
M
CD177 Ly6G DAPI
HoxB8 (undiff.)
HoxB8 (diff.)
N
adherent cells/FOV
rolling cells/FOV

Fig. S7. | (A) Longitudinal sepsis scoring of n=4 mice per group after i.p. injection of sodium chloride or 1 mg/kg BW LPS. Two-way ANOVA. (B) Absolute mean fluorescence intensities (MFIs) of the indicated surface receptors, measured on blood neutrophils from mice injected with NaCl or LPS. Student's t-test, two-tailed, unpaired. (C) Quantification of normalized CD177 MFI of murine peripheral blood neutrophil following i.p. injection of PBS or LPS at the indicated concentrations and incubation for 2 h. One-way ANOVA with Dunnett's post-hoc testing. (D) Quantification of normalized CD177 MFI of human peripheral blood neutrophils incubated with the indicated LPS concentrations *in vitro* for 2 h. One-way ANOVA with Dunnett's post-hoc testing. (E) Flow-cytometric quantification of neutrophil CD177 MFIs and infiltrating neutrophils in the liver and spleen following i.p. injection of PBS or the indicated LPS concentrations and subsequent incubation for two hours. One-way ANOVA with Dunnett's post-hoc testing. (F) Experimental scheme of adoptive transfer experiments with isolated murine neutrophils. (G) Quantification of % neutrophils of circulating WBC of donor mice treated with NaCl or LPS and MFI measurements of sham-treated donor cells marked with CellTracker blue and LPS-treated ones marked with CellTracker FarRed, respectively. Student's t-test, two-tailed, unpaired. (H) MFI quantification of CD177, CD11b and CD66a for the indicated groups. Two-way ANOVA with post-hoc Dunnett's testing. (I) Quantification of phagocytic indices (representing the number of phagocytosed E. coli particles) and % E. coli-positive murine neutrophils following co-incubation with pHrodo-labelled E. coli bioparticles. Student's t-test, two-tailed, unpaired. (J) Representative curves of intracellular calcium currents in isolated murine neutrophils as assessed through Fluo-4 fluorescence signal following incubation with fMLP. Two-way ANOVA with post-hoc Dunnett's testing. (K) Representative confocal images of human neutrophils stimulated with 500 nM PMA in the presence of IgG2a or anti-CD177 antibody. Scale bar 50 µm. Right panels: Quantification of % Sytox$^+$ neutrophils for murine peripheral blood and human peripheral blood neutrophils. In some cases, blood samples from two mice were pooled to acquire sufficient numbers of blood neutrophils. Student's t-test, two-tailed, unpaired. (L) Quantification of ROS production as assessed by staining of murine neutrophils with 2,7 DCFDA following incubation with 100nM PMA. Student's t-test, two-tailed, unpaired. (M) Representative immunofluorescence stainings of undifferentiated HoxB8 cells (left panel) and HoxB8-derived differentiated neutrophils (right panel). Scale bar 20 µm. (N) Quantification of

adherent and rolling HoxB8-derived neutrophils on P-selectin coated chambers under flow (see Suppl. Methods section).

Fig. S8

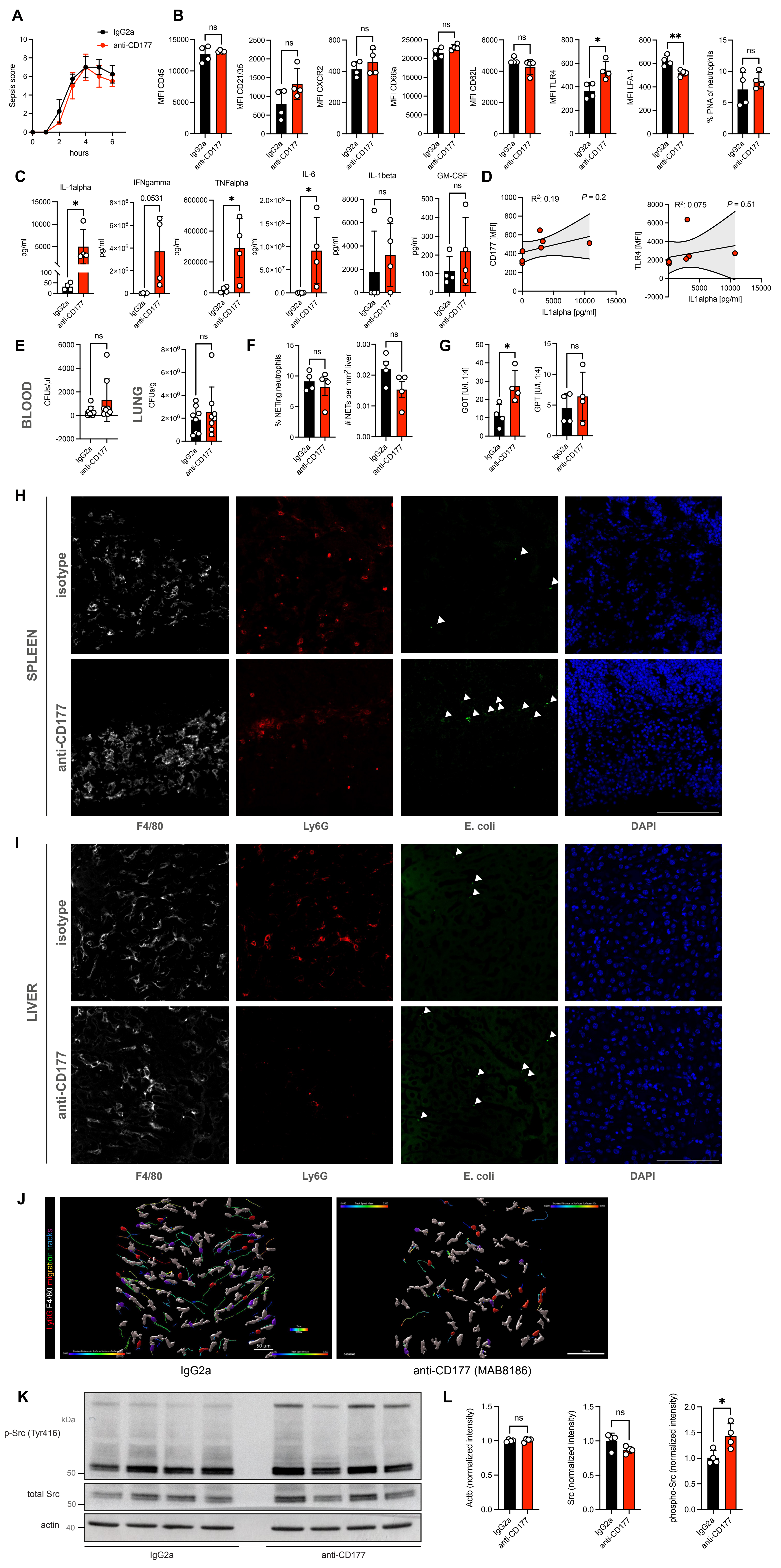

A
IgG2a
anti-CD177
Sepsis score
hours
B
ns
MFI CD45
MFI CD21/35
MFI CXCR2
MFI CD66a
MFI CD62L
MFI TLR4
MFI LFA-1
% PNA of neutrophils
IgG2a
anti-CD177
C
IL-1alpha
IFNgamma
0.0531
TNFalpha
IL-6
IL-1beta
GM-CSF
pg/ml
D
R^2: 0.19
P = 0.2
CD177 [MFI]
IL1alpha [pg/ml]
R^2: 0.075
P = 0.51
TLR4 [MFI]
E
BLOOD
CFUs/µl
LUNG
CFUs/g
F
% NETing neutrophils
# NETs per mm^2 liver
G
GOT [U/l, 1:4]
GPT [U/l, 1:4]
H
SPLEEN
isotype
anti-CD177
F4/80
Ly6G
E. coli
DAPI
I
LIVER
J
Ly6G F4/80 migration tracks
50 µm
IgG2a
anti-CD177 (MAB8186)
K
kDa
p-Src (Tyr416)
total Src
actin
50
40
L
Actb (normalized intensity)
Src (normalized intensity)
phospho-Src (normalized intensity)

Fig. S8. | (A) Visualiziation of sepsis scores in IgG2a- or anti-CD177-pretreated animals i.v. injected with E. coli over time. (B) Quantification of MFIs for the indicated markers and platelet-neutrophil aggregates (PNAs) across experimental groups. Student's t-test, two-tailed, unpaired. (C) Quantification of plasma cytokines from IgG2a- or anti-CD177-antibody treated mice injected with *E. coli* in pg/ml. Student's t-test, two-tailed, unpaired. (D) Linear regression analyses, showing no significant correlation between CD177 and TLR4 MFIs and systemic IL1alpha levels in individual experimental animals. (E) Quantification of NETing neutrophils and NETs per mm^2 of liver tissue after i.v. injection of E. coli and treatment with IgG2a or anti-CD177 antibody. Student's t-test, two-tailed, unpaired. (F) Quantification of colony-forming units (CFUs) of blood or lung tissue harvested from E. coli-injected mice treated with IgG2a or anti-CD177 antibody. Student's t-test, two-tailed, unpaired. (G) Quantification of 1:4-diluted plasma levels of GOT and GPT in *E. coli*-injected mice treated with IgG2a or anti-CD177 antibody. Student's t-test, two-tailed, unpaired. (H) Representative confocal images of spleen sections collected from different experimental groups, corresponding to Fig. 4. Scale bar = 100 µm. (I) Representative confocal images of spleen sections collected from different experimental groups, corresponding to Fig. 4. Scale bar = 100 µm. (J) Representative rendered confocal images from *in vivo* microscopy as well as migration tracks (rainbow colors) for treatment groups, corresponding to Fig. 4. White cells = hepatic Kupffer cells. Purple neutrophils are currently or have been in physical contact with Kupffer cells. Scale bar 50 µm. (K) Western blot of murine neutrophil lysates stained for the indicated antigens after incubating isolated murine neutrophils with IgG2a or anti-CD177 (10 µg/ml each) for 1 h. Lanes 1-4, IgG2a treatment; Lanes 5-8, anti-CD177 treatment (10 µg/ml). (L) Quantification of normalized intensity of ACTB, total Src and phospho-Src (Tyr416) of neutrophil lysates shown in H. Student's t-test, two-tailed, unpaired.

Fig. S9

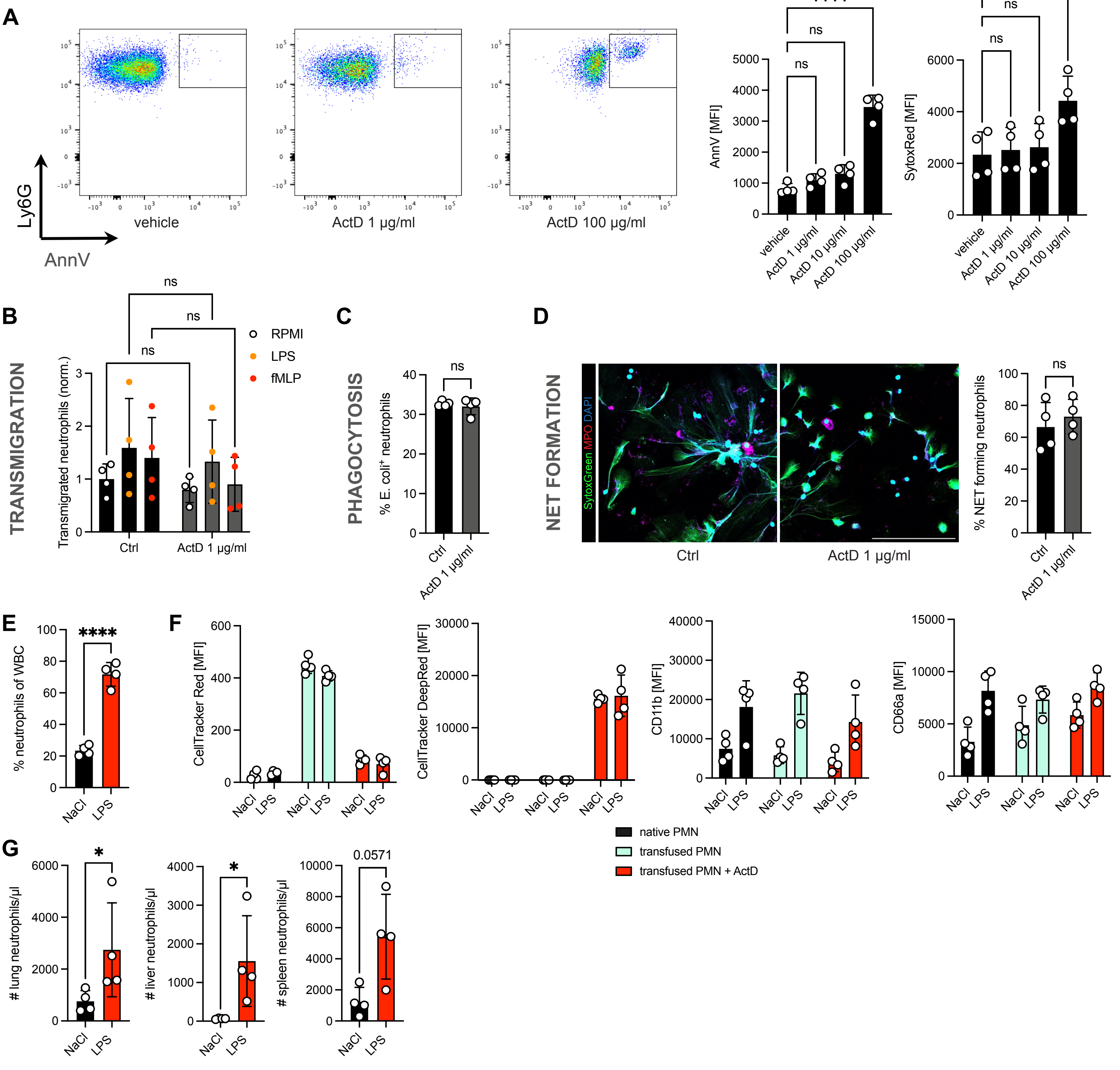
A
Ly6G
AnnV
vehicle
ActD 1 µg/ml
ActD 100 µg/ml
AnnV [MFI]
SytoxRed [MFI]
vehicle
ActD 1 µg/ml
ActD 10 µg/ml
ActD 100 µg/ml
****
*
ns
B
TRANSMIGRATION
Transmigrated neutrophils (norm.)
RPMI
LPS
fMLP
Ctrl
ActD 1 µg/ml
C
PHAGOCYTOSIS
% E. coli+ neutrophils
D
NET FORMATION
SytoxGreen MPO DAPI
% NET forming neutrophils
E
% neutrophils of WBC
NaCl
LPS
F
CellTracker Red [MFI]
CellTracker DeepRed [MFI]
CD11b [MFI]
CD66a [MFI]
native PMN
transfused PMN
transfused PMN + ActD
G
lung neutrophils/µl
liver neutrophils/µl
spleen neutrophils/µl
0.0571

Fig. S9. | (A) Representative scatter plots of murine neutrophils treated with vehicle or the indicated concentrations of actinomycin D and quantification of MFIs of AnnV (indicating apoptosis) and Sytox (indicating cell death) for vehicle or ActD-treated neutrophils with the indicated concentrations. One-way ANOVA with Dunnett's post-hoc testing. (B) Normalized quantification of transmigration in vehicle- or ActD-treated neutrophils across a 3 µm membrane towards the indicated chemoattractants (RPMI vs 10 µg/ml LPS vs 10 µM fMLP). Two-way ANOVA with Dunnett's post-hoc testing. (C) Quantification of neutrophil phagocytosis in the presence of vehicle or ActD 1 µg/ml. Student's t-test, two-tailed, unpaired. (D) Representative micrographs and quantification of NET formation assay in the presence of vehicle or ActD (1 µg/ml). Student's t-test, two-tailed, unpaired. Scale bar 100 µm. (E) Quantification of relative neutrophil amounts of all leukocytes in NaCl- vs. LPS-treated animals. (F) Quantification of MFIs for the indicated CellTrackers and surface receptors shown for either native or differentially labelled transfused neutrophils. (G) Quantification of infiltrating neutrophil numbers for the indicated organs, as assessed by flow cytometry of digested organs, including host and transfused neutrophils for mice following i.p. injection with NaCl or LPS 1 mg/kg BW. Student's t-test, two-tailed, unpaired.

Fig. S10

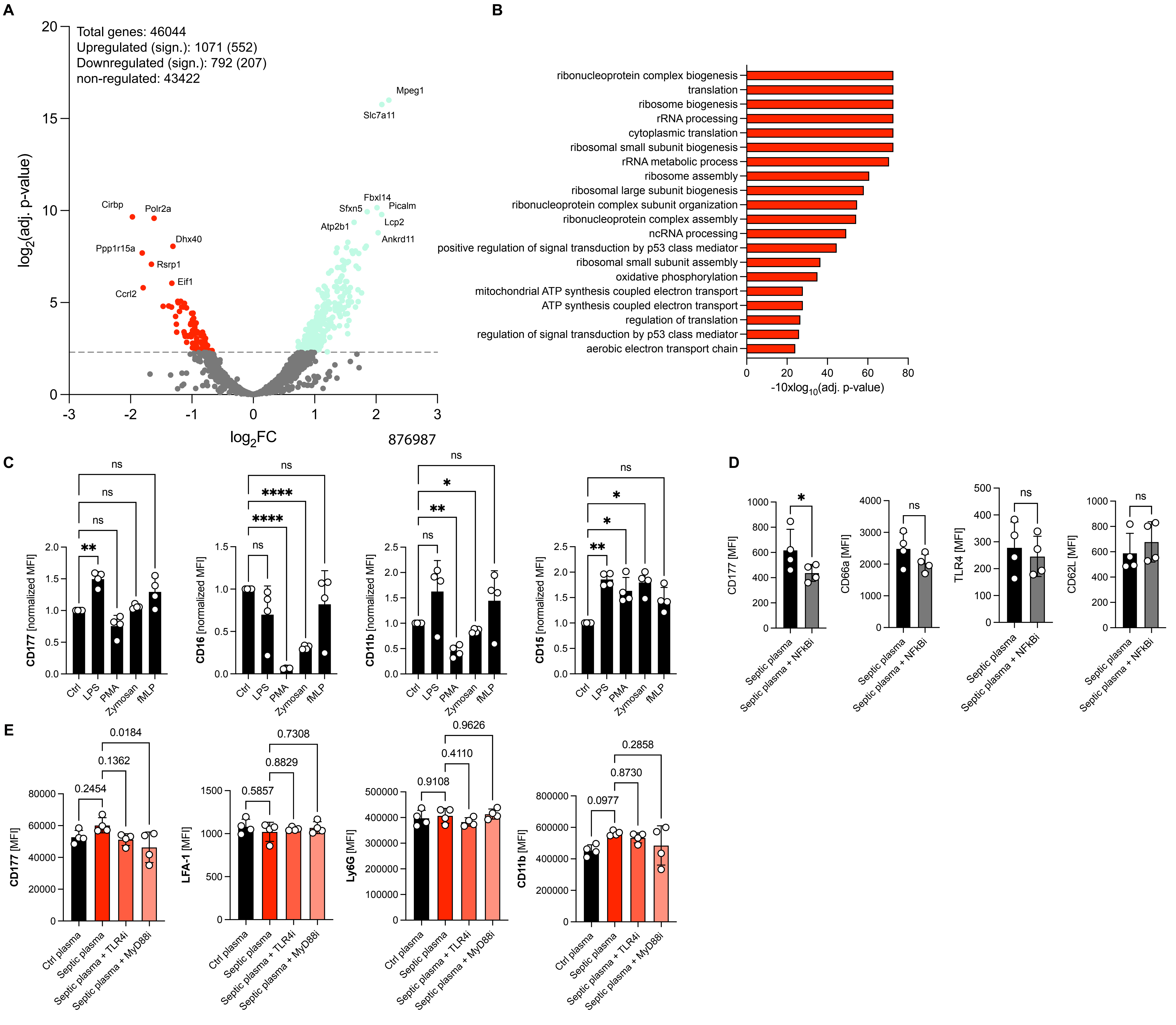
A
Total genes: 46044
Upregulated (sign.): 1071 (552)
Downregulated (sign.): 792 (207)
non-regulated: 43422
log2(adj. p-value)
log2FC
876987
Mpeg1
Slc7a11
Fbxl14
Sfxn5
Picalm
Atp2b1
Lcp2
Ankrd11
Cirbp
Polr2a
Dhx40
Ppp1r15a
Rsrp1
Eif1
Ccrl2
B
ribonucleoprotein complex biogenesis
translation
ribosome biogenesis
rRNA processing
cytoplasmic translation
ribosomal small subunit biogenesis
rRNA metabolic process
ribosome assembly
ribosomal large subunit biogenesis
ribonucleoprotein complex subunit organization
ribonucleoprotein complex assembly
ncRNA processing
positive regulation of signal transduction by p53 class mediator
ribosomal small subunit assembly
oxidative phosphorylation
mitochondrial ATP synthesis coupled electron transport
ATP synthesis coupled electron transport
regulation of translation
regulation of signal transduction by p53 class mediator
aerobic electron transport chain
-10xlog10(adj. p-value)
C
CD177 [normalized MFI]
CD16 [normalized MFI]
CD11b [normalized MFI]
CD15 [normalized MFI]
Ctrl
LPS
PMA
Zymosan
fMLP
ns
**
*
****
D
CD177 [MFI]
CD66a [MFI]
TLR4 [MFI]
CD62L [MFI]
Septic plasma
Septic plasma + NFkBi
E
CD177 [MFI]
LFA-1 [MFI]
Ly6G [MFI]
CD11b [MFI]
Ctrl plasma
Septic plasma
Septic plasma + TLR4i
Septic plasma + MyD88i
0.2454
0.1362
0.0184
0.5857
0.8829
0.7308
0.9108
0.4110
0.9626
0.0977
0.8730
0.2858

Fig. S10. | (A) Volcano plot depicting genes detected in RNA bulk sequencing of isolated murine neutrophils following exposure to septic plasma in the presence or absence of actinomycin D (see Fig. 5J-L). The dotted line indicates the significance threshold (adj. p-value < 0.05), the colored dots represent significantly up- (light green) or downregulated genes (red) comparing neutrophil transcriptomes following sepsis treatment without compared to with ActD. (B) Gene set enrichment analysis (GSEA) showing top20 downregulated biological processes in sham- vs. ActD-exposed neutrophils in response to septic plasma. (C) Mouse neutrophils were incubated at 37°C, 5% CO_2 with the indicated agonists (LPS: 10 µg/ml, fMLP: 10 µM, Zymosan 1 µM, PMA 100 nM) for 6 h followed by flow-cytometric measurement of the indicated surface markers. One-way ANOVA with Dunnett's post-hoc testing. (D, E) Murine neutrophils were incubated with plasma from healthy or septic mice for 4-6 h in the presence of vehicle or NF-kappaB Activation Inhibitor VI, benzoxathiole compound (ab145954, 10 µM), the TLR4-blocking peptide C34 (10 µg/ml) or the MyD88 inhibitor TJ-M2010-5 (50 µM). MFIs of live neutrophils for the indicated surface markers were measured by flow cytometry. D: Student's t-test, two-tailed, unpaired; E: One-way ANOVA with Dunnett's post-hoc testing.

Fig. S11

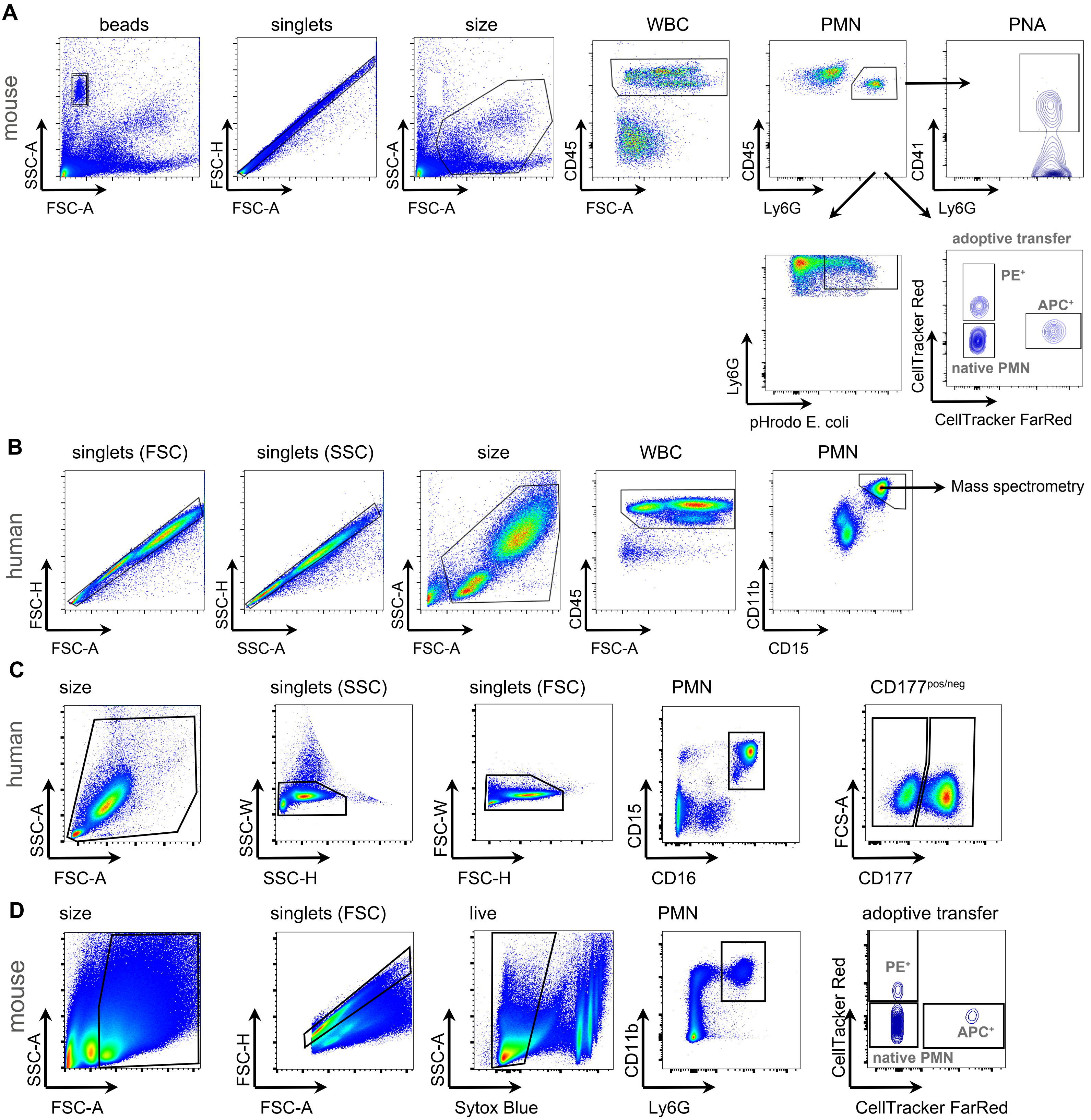
A
beads
singlets
size
WBC
PMN
PNA
mouse
SSC-A
FSC-A
FSC-H
CD45
Ly6G
CD41
adoptive transfer
PE+
APC+
native PMN
CellTracker Red
CellTracker FarRed
pHrodo E. coli
B
singlets (FSC)
singlets (SSC)
human
SSC-H
CD11b
CD15
Mass spectrometry
C
CD177pos/neg
SSC-W
FSC-W
CD16
FCS-A
CD177
D
live
Sytox Blue

Fig. S11. | (A) Gating strategy targeting neutrophils in peripheral mouse blood, with analysis of platelet-neutrophil aggregates (PNAs), pHrodo-positive neutrophils as well as transfused neutrophils labelled with CellTracker Red or CellTracker FarRed (see Suppl. Materials and Methods). (B) Gating strategy of fixated, cryoconserved human neutrophils for fluorescence-activated cell sorting (FACS) prior to mass spectrometry. (C) Gating strategy of fixated, cryoconserved human neutrophils used for neutrophil diversity panel depicted in Fig. 3. (D) Gating strategy for digested, homogenized murine splenic tissue to detect both native and transfused neutrophils labelled with CellTracker Red or CellTracker FarRed.

Suppl. Movie legends

Suppl. Movie S1: Live imaging of HoxB8-derived neutrophil adhesion and rolling assay. Live imaging of HoxB8-derived neutrophil adhesion and rolling assay. Cells were pre-incubated with either isotype (first video) or a CD177-blocking antibody (second video, each 10 µg/ml) for 10 min and subsequently transfused over a P-selectin-coated surface. See corresponding Fig. S7M, N. PH = phase contrast.

Suppl. Movie S2: Live confocal microscopy of neutrophils in the infected liver. Live confocal microscopy of neutrophils (red), *E.coli* (green), and Kupffer cells (KCs, white) in the infected liver after treatment with either isotype (IgG2a) or anti-CD177 antibody (1 mg/kg BW). Scale bar, 100 µm. Time is indicated in h:min:s. The second movie of each treatment shows the corresponding 3D-reconstruction with visualization of cell tracks and analysis of neutrophil-KC-colocalization. Different colors of the tracks represent different mean velocities (µm/s) of the tracks, as indicated by the color bar in the upper left corner. Neutrophils in direct contact to KCs are highlighted in purple, neutrophils at a distance of >0.001 µm from KCs are highlighted in red (represented by color bar in the upper right corner). See corresponding Fig. 5B and Fig. S8J.

Suppl. Movie S3: Live imaging of HoxB8-derived neutrophil detachment assay. Live imaging of HoxB8-derived neutrophil detachment assay. Cells were pre-incubated with either isotype (first video) or a CD177-blocking antibody (second video, each 10 µg/ml) for 10 min, allowed to adhere on a CXCL-1-/P-selectin-/ ICAM-1-coated surface and subsequently perfused with increasing shear stress (as indicated). See corresponding Fig. 5D, E. PH = phase contrast.

REFERENCES AND NOTES

1. K. Ley, H. M. Hoffman, P. Kubes, M. A. Cassatella, A. Zychlinsky, C. C. Hedrick, S. D. Catz, Neutrophils: New insights and open questions. *Sci. Immunol.* **3**, eaat4579 (2018).

2. A. Margraf, C. A. Lowell, A. Zarbock, Neutrophils in acute inflammation: Current concepts and translational implications. *Blood* **139**, 2130–2144 (2022).

3. E. Kolaczkowska, P. Kubes, Neutrophil recruitment and function in health and inflammation. *Nat. Rev. Immunol.* **13**, 159–175 (2013).

4. C. Silvestre-Roig, Z. G. Fridlender, M. Glogauer, P. Scapini, Neutrophil diversity in health and disease. *Trends Immunol.* **40**, 565–583 (2019).

5. J. Skokowa, D. C. Dale, I. P. Touw, C. Zeidler, K. Welte, Severe congenital neutropenias. *Nat. Rev. Dis. Primers.* **3**, 17032 (2017).

6. G. A. Arnadottir, G. L. Norddahl, S. Gudmundsdottir, A. B. Agustsdottir, S. Sigurdsson, B. O. Jensson, K. Bjarnadottir, F. Theodors, S. Benonisdottir, E. V. Ivarsdottir, A. Oddsson, R. P. Kristjansson, G. Sulem, K. F. Alexandersson, T. Juliusdottir, K. R. Gudmundsson, J. Saemundsdottir, A. Jonasdottir, A. Jonasdottir, A. Sigurdsson, P. Manzanillo, S. A. Gudjonsson, G. A. Thorisson, O. T. Magnusson, G. Masson, K. B. Orvar, H. Holm, S. Bjornsson, R. Arngrimsson, D. F. Gudbjartsson, U. Thorsteinsdottir, I. Jonsdottir, A. Haraldsson, P. Sulem, K. Stefansson, A homozygous loss-of-function mutation leading to CYBC1 deficiency causes chronic granulomatous disease. *Nat. Commun.* **9**, 4447 (2018).

7. J. D. Pollock, D. A. Williams, M. A. Gifford, L. L. Li, X. Du, J. Fisherman, S. H. Orkin, C. M. Doerschuk, M. C. Dinauer, Mouse model of X-linked chronic granulomatous disease, an inherited defect in phagocyte superoxide production. *Nat. Genet.* **9**, 202–209 (1995).

8. J. M. Adrover, A. Aroca-Crevillen, G. Crainiciuc, F. Ostos, Y. Rojas-Vega, A. Rubio-Ponce, C. Cilloniz, E. Bonzon-Kulichenko, E. Calvo, D. Rico, M. A. Moro, C. Weber, I. Lizasoain, A. Torres, J. Ruiz-Cabello, J. Vazquez, A. Hidalgo, Programmed 'disarming' of the neutrophil proteome reduces the magnitude of inflammation. *Nat. Immunol.* **21**, 135–144 (2020).

9. C. Silvestre-Roig, A. Hidalgo, O. Soehnlein, Neutrophil heterogeneity: Implications for homeostasis and pathogenesis. *Blood* **127**, 2173–2181 (2016).

10. B. Uhl, Y. Vadlau, G. Zuchtriegel, K. Nekolla, K. Sharaf, F. Gaertner, S. Massberg, F. Krombach, C. A. Reichel, Aged neutrophils contribute to the first line of defense in the acute inflammatory response. *Blood* **128**, 2327–2337 (2016).

11. L. G. Ng, R. Ostuni, A. Hidalgo, Heterogeneity of neutrophils. *Nat. Rev. Immunol.* **19**, 255–265 (2019).

12. G. Crainiciuc, M. Palomino-Segura, M. Molina-Moreno, J. Sicilia, D. G. Aragones, J. L. Y. Li, R. Madurga, J. M. Adrover, A. Aroca-Crevillen, S. Martin-Salamanca, A. S. Del Valle, S. D. Castillo, H. C. E. Welch, O. Soehnlein, M. Graupera, F. Sanchez-Cabo, A. Zarbock, T. E. Smithgall, M. Di Pilato, T. R. Mempel, P. L. Tharaux, S. F. Gonzalez, A. Ayuso-Sacido, L. G. Ng, G. F. Calvo, I. Gonzalez-Diaz, F. Diaz-de-Maria, A. Hidalgo, Behavioural immune landscapes of inflammation. *Nature* **601**, 415–421 (2022).

13. L. Koenderman, K. Tesselaar, N. Vrisekoop, Human neutrophil kinetics: A call to revisit old evidence. *Trends Immunol.* **43**, 868–876 (2022).

14. R. Xue, Q. Zhang, Q. Cao, R. Kong, X. Xiang, H. Liu, M. Feng, F. Wang, J. Cheng, Z. Li, Q. Zhan, M. Deng, J. Zhu, Z. Zhang, N. Zhang, Liver tumour immune microenvironment subtypes and neutrophil heterogeneity. *Nature* **612**, 141–147 (2022).

15. R. Zilionis, C. Engblom, C. Pfirschke, V. Savova, D. Zemmour, H. D. Saatcioglu, I. Krishnan, G. Maroni, C. V. Meyerovitz, C. M. Kerwin, S. Choi, W. G. Richards, A. De Rienzo, D. G. Tenen, R. Bueno, E. Levantini, M. J. Pittet, A. M. Klein, Single-cell transcriptomics of human and mouse lung cancers reveals conserved myeloid populations across individuals and species. *Immunity* **50**, 1317–1334.e10 (2019).

16. D. F. Quail, B. Amulic, M. Aziz, B. J. Barnes, E. Eruslanov, Z. G. Fridlender, H. S. Goodridge, Z. Granot, A. Hidalgo, A. Huttenlocher, M. J. Kaplan, I. Malanchi, T. Merghoub, E. Meylan, V. Mittal, M. J. Pittet, A. Rubio-Ponce, I. A. Udalova, T. K. van den Berg, D. D. Wagner, P. Wang, A. Zychlinsky, K.

E. de Visser, M. Egeblad, P. Kubes, Neutrophil phenotypes and functions in cancer: A consensus statement. *J. Exp. Med.* **219**, e20220011 (2022).

17. R. Kaiser, A. Leunig, K. Pekayvaz, O. Popp, M. Joppich, V. Polewka, R. Escaig, A. Anjum, M. L. Hoffknecht, C. Gold, S. Brambs, A. Engel, S. Stockhausen, V. Knottenberg, A. Titova, M. Haji, C. Scherer, M. Muenchhoff, J. C. Hellmuth, K. Saar, B. Schubert, A. Hilgendorff, C. Schulz, S. Kaab, R. Zimmer, N. Hubner, S. Massberg, P. Mertins, L. Nicolai, K. Stark, Self-sustaining IL-8 loops drive a prothrombotic neutrophil phenotype in severe COVID-19. *JCI Insight* **6**, e150862 (2021).

18. L. Nicolai, A. Leunig, S. Brambs, R. Kaiser, M. Joppich, M. L. Hoffknecht, C. Gold, A. Engel, V. Polewka, M. Muenchhoff, J. C. Hellmuth, A. Ruhle, S. Ledderose, T. Weinberger, H. Schulz, C. Scherer, M. Rudelius, M. Zoller, O. T. Keppler, B. Zwissler, M. von Bergwelt-Baildon, S. Kaab, R. Zimmer, R. D. Bulow, S. von Stillfried, P. Boor, S. Massberg, K. Pekayvaz, K. Stark, Vascular neutrophilic inflammation and immunothrombosis distinguish severe COVID-19 from influenza pneumonia. *J. Thromb. Haemost.* **19**, 574–581 (2021).

19. L. Nicolai, A. Leunig, S. Brambs, R. Kaiser, T. Weinberger, M. Weigand, M. Muenchhoff, J. C. Hellmuth, S. Ledderose, H. Schulz, C. Scherer, M. Rudelius, M. Zoller, D. Hochter, O. Keppler, D. Teupser, B. Zwissler, M. von Bergwelt-Baildon, S. Kaab, S. Massberg, K. Pekayvaz, K. Stark, J. S. Authors, Immunothrombotic dysregulation in COVID-19 pneumonia is associated with respiratory failure and coagulopathy. *Circulation* **142**, 1176–1189 (2020).

20. J. Schulte-Schrepping, N. Reusch, D. Paclik, K. Baßler, S. Schlickeiser, B. Zhang, B. Krämer, T. Krammer, S. Brumhard, L. Bonaguro, E. De Domenico, D. Wendisch, M. Grasshoff, T. S. Kapellos, M. Beckstette, T. Pecht, A. Saglam, O. Dietrich, H. E. Mei, A. R. Schulz, C. Conrad, D. Kunkel, E. Vafadarnejad, C. J. Xu, A. Horne, M. Herbert, A. Drews, C. Thibeault, M. Pfeiffer, S. Hippenstiel, A. Hocke, H. Müller-Redetzky, K. M. Heim, F. Machleidt, A. Uhrig, L. Bosquillon de Jarcy, L. Jürgens, M. Stegemann, C. R. Glösenkamp, H. D. Volk, C. Goffinet, M. Landthaler, E. Wyler, P. Georg, M. Schneider, C. Dang-Heine, N. Neuwinger, K. Kappert, R. Tauber, V. Corman, J. Raabe, K. M. Kaiser, M. T. Vinh, G. Rieke, C. Meisel, T. Ulas, M. Becker, R. Geffers, M. Witzenrath, C. Drosten, N. Suttorp, C. von Kalle, F. Kurth, K. Händler, J. L. Schultze, A. C. Aschenbrenner, Y. Li, J. Nattermann, B.

Sawitzki, A. E. Saliba, L. E. Sander, Severe COVID-19 is marked by a dysregulated myeloid cell compartment. *Cell* **182**, 1419–1440.e23 (2020).

21. X. Xie, Q. Shi, P. Wu, X. Zhang, H. Kambara, J. Su, H. Yu, S. Y. Park, R. Guo, Q. Ren, S. Zhang, Y. Xu, L. E. Silberstein, T. Cheng, F. Ma, C. Li, H. R. Luo, Single-cell transcriptome profiling reveals neutrophil heterogeneity in homeostasis and infection. *Nat. Immunol.* **21**, 1119–1133 (2020).

22. T. E. Khoyratty, Z. Ai, I. Ballesteros, H. L. Eames, S. Mathie, S. Martin-Salamanca, L. Wang, A. Hemmings, N. Willemsen, V. von Werz, A. Zehrer, B. Walzog, E. van Grinsven, A. Hidalgo, I. A. Udalova, Distinct transcription factor networks control neutrophil-driven inflammation. *Nat. Immunol.* **22**, 1093–1106 (2021).

23. H. Alshetaiwi, N. Pervolarakis, L. L. McIntyre, D. Ma, Q. Nguyen, J. A. Rath, K. Nee, G. Hernandez, K. Evans, L. Torosian, A. Silva, C. Walsh, K. Kessenbrock, Defining the emergence of myeloid-derived suppressor cells in breast cancer using single-cell transcriptomics. *Sci. Immunol.* **5**, eaay6017 (2020).

24. A. J. Kwok, A. Allcock, R. C. Ferreira, E. Cano-Gamez, M. Smee, K. L. Burnham, Y. X. Zurke, S. McKechnie, A. J. Mentzer, C. Monaco, I. A. Udalova, C. J. Hinds, J. A. Todd, E. E. Davenport, J. C. Knight, Neutrophils and emergency granulopoiesis drive immune suppression and an extreme response endotype during sepsis. *Nat. Immunol.* **24**, 767–779 (2023).

25. L. Kuri-Cervantes, M. B. Pampena, W. Meng, A. M. Rosenfeld, C. A. G. Ittner, A. R. Weisman, R. S. Agyekum, D. Mathew, A. E. Baxter, L. A. Vella, O. Kuthuru, S. A. Apostolidis, L. Bershaw, J. Dougherty, A. R. Greenplate, A. Pattekar, J. Kim, N. Han, S. Gouma, M. E. Weirick, C. P. Arevalo, M. J. Bolton, E. C. Goodwin, E. M. Anderson, S. E. Hensley, T. K. Jones, N. S. Mangalmurti, E. T. Luning Prak, E. J. Wherry, N. J. Meyer, M. R. Betts, Comprehensive mapping of immune perturbations associated with severe COVID-19. *Sci. Immunol.* **5**, eabd7114 (2020).

26. A. Meghraoui-Kheddar, B. G. Chousterman, N. Guillou, S. M. Barone, S. Granjeaud, H. Vallet, A. Corneau, K. Guessous, C. de Roquetaillade, A. Boissonnas, J. M. Irish, C. Combadiere, Two new neutrophil subsets define a discriminating sepsis signature. *Am. J. Respir. Crit. Care Med.* **205**, 46–59 (2022).

27. R. Spijkerman, S. H. Bongers, B. J. J. Bindels, G. H. Tinnevelt, G. Giustarini, N. K. N. Jorritsma, W. Buitenwerf, D. E. J. van Spengler, E. M. Delemarre, S. Nierkens, H. M. R. van Goor, J. J. Jansen, N. Vrisekoop, F. Hietbrink, L. P. H. Leenen, K. A. H. Kaasjager, L. Koenderman; COVPACH study group, Flow cytometric evaluation of the neutrophil compartment in COVID-19 at hospital presentation: A normal response to an abnormal situation. *J. Leukoc. Biol.* **109**, 99–114 (2021).

28. K. N. Kangelaris, R. Clemens, X. Fang, A. Jauregui, T. Liu, K. Vessel, T. Deiss, P. Sinha, A. Leligdowicz, K. D. Liu, H. Zhuo, M. N. Alder, H. R. Wong, C. S. Calfee, C. Lowell, M. A. Matthay, A neutrophil subset defined by intracellular olfactomedin 4 is associated with mortality in sepsis. *Am. J. Physiol. Lung Cell. Mol. Physiol.* **320**, L892–L902 (2021).

29. R. Grieshaber-Bouyer, T. Exner, N. S. Hackert, F. A. Radtke, S. A. Jelinsky, O. Halyabar, A. Wactor, E. Karimizadeh, J. Brennan, J. Schettini, H. Jonsson, D. A. Rao, L. A. Henderson, C. Muller-Tidow, H. M. Lorenz, G. Wabnitz, J. A. Lederer, A. Hadjipanayis, P. A. Nigrovic, Ageing and interferon gamma response drive the phenotype of neutrophils in the inflamed joint. *Ann. Rheum. Dis.* **81**, 805–814 (2022).

30. S. Sinha, N. L. Rosin, R. Arora, E. Labit, A. Jaffer, L. Cao, R. Farias, A. P. Nguyen, L. G. N. de Almeida, A. Dufour, A. Bromley, B. McDonald, M. R. Gillrie, M. J. Fritzler, B. G. Yipp, J. Biernaskie, Dexamethasone modulates immature neutrophils and interferon programming in severe COVID-19. *Nat. Med.* **28**, 201–211 (2022).

31. Y. Tian, L. N. Carpp, H. E. R. Miller, M. Zager, E. W. Newell, R. Gottardo, Single-cell immunology of SARS-CoV-2 infection. *Nat. Biotechnol.* **40**, 30–41 (2022).

32. A. J. Wilk, A. Rustagi, N. Q. Zhao, J. Roque, G. J. Martinez-Colon, J. L. McKechnie, G. T. Ivison, T. Ranganath, R. Vergara, T. Hollis, L. J. Simpson, P. Grant, A. Subramanian, A. J. Rogers, C. A. Blish, A single-cell atlas of the peripheral immune response in patients with severe COVID-19. *Nat. Med.* **26**, 1070–1076 (2020).

33. H. Lei, C. Wang, Y. Wang, C. Wang, Single-cell RNA-Seq revealed profound immune alteration in the peripheral blood of patients with bacterial infection. *Int. J. Infect. Dis.* **103**, 527–535 (2021).

34. T. Honda, T. Uehara, G. Matsumoto, S. Arai, M. Sugano, Neutrophil left shift and white blood cell count as markers of bacterial infection. *Clin. Chim. Acta* **457**, 46–53 (2016).

35. R. Grieshaber-Bouyer, F. A. Radtke, P. Cunin, G. Stifano, A. Levescot, B. Vijaykumar, N. Nelson-Maney, R. B. Blaustein, P. A. Monach, P. A. Nigrovic; ImmGen Consortium, The neutrotime transcriptional signature defines a single continuum of neutrophils across biological compartments. *Nat. Commun.* **12**, 2856 (2021).

36. I. Ballesteros, A. Rubio-Ponce, M. Genua, E. Lusito, I. Kwok, G. Fernández-Calvo, T. E. Khoyratty, E. van Grinsven, S. González-Hernández, J. Nicolás-Ávila, T. Vicanolo, A. Maccataio, A. Benguría, J. L. Li, J. M. Adrover, A. Aroca-Crevillen, J. A. Quintana, S. Martín-Salamanca, F. Mayo, S. Ascher, G. Barbiera, O. Soehnlein, M. Gunzer, F. Ginhoux, F. Sánchez-Cabo, E. Nistal-Villán, C. Schulz, A. Dopazo, C. Reinhardt, I. A. Udalova, L. G. Ng, R. Ostuni, A. Hidalgo, Co-option of neutrophil fates by tissue environments. *Cell* **183**, 1282–1297.e18 (2020).

37. M. Palomino-Segura, J. Sicilia, I. Ballesteros, A. Hidalgo, Strategies of neutrophil diversification. *Nat. Immunol.* **24**, 575–584 (2023).

38. K. Pekayvaz, M. Joppich, S. Brambs, V. Knottenberg, L. Eivers, A. Martinez-Navarro, R. Kaiser, N. Meissner, B. Kilani, S. Stockhausen, A. J. Janjic, V. Polewka, F. W. Wendler, A. D. zu Senden, A. Leunig, M. Voelkl, B. Engelmann, M. R. H. Petzsche, T. Boeckh-Behrens, T. Liebig, M. Dichgans, W. Enard, R. Zimmer, S. Tiedt, S. Massberg, L. Nicolai, K. Stark, Immunothrombolytic monocyte-neutrophil axes dominate the single-cell landscape of human thrombosis. bioRxiv 2024.01.10.574518 (2024). https://doi.org/10.1101/2024.01.10.574518.

39. M. Stoeckius, C. Hafemeister, W. Stephenson, B. Houck-Loomis, P. K. Chattopadhyay, H. Swerdlow, R. Satija, P. Smibert, Simultaneous epitope and transcriptome measurement in single cells. *Nat. Methods* **14**, 865–868 (2017).

40. A. P. N. Ambagala, J. C. Solheim, S. Srikumaran, Viral interference with MHC class I antigen presentation pathway: The battle continues. *Vet. Immunol. Immunopathol.* **107**, 1–15 (2005).

41. A. Nakano, T. Harada, S. Morikawa, Y. Kato, Expression of leukocyte common antigen (CD45) on various human leukemia/lymphoma cell lines. *Acta Pathol. Jpn.* **40**, 107–115 (1990).

42. F. A. Wolf, F. K. Hamey, M. Plass, J. Solana, J. S. Dahlin, B. Gottgens, N. Rajewsky, L. Simon, F. J. Theis, PAGA: Graph abstraction reconciles clustering with trajectory inference through a topology preserving map of single cells. *Genome Biol.* **20**, 59 (2019).

43. M. Toufiq, J. Roelands, M. Alfaki, B. S. A. Kabeer, M. Saadaoui, A. P. Lakshmanan, D. K. Bangarusamy, S. Murugesan, D. Bedognetti, W. Hendrickx, S. Al Khodor, A. Terranegra, D. Rinchai, D. Chaussabel, M. Garand, Annexin A3 in sepsis: Novel perspectives from an exploration of public transcriptome data. *Immunology* **161**, 291–302 (2020).

44. G. Zhou, L. Yu, L. Fang, W. Yang, T. Yu, Y. Miao, M. Chen, K. Wu, F. Chen, Y. Cong, Z. Liu, $CD177^+$ neutrophils as functionally activated neutrophils negatively regulate IBD. *Gut* **67**, 1052–1063 (2018).

45. P. Langfelder, S. Horvath, WGCNA: An R package for weighted correlation network analysis. *BMC Bioinformatics* **9**, 559 (2008).

46. S. W. Kazer, T. P. Aicher, D. M. Muema, S. L. Carroll, J. Ordovas-Montanes, V. N. Miao, A. A. Tu, C. G. K. Ziegler, S. K. Nyquist, E. B. Wong, N. Ismail, M. Dong, A. Moodley, B. Berger, J. C. Love, K. L. Dong, A. Leslie, Z. M. Ndhlovu, T. Ndung'u, B. D. Walker, A. K. Shalek, Integrated single-cell analysis of multicellular immune dynamics during hyperacute HIV-1 infection. *Nat. Med.* **26**, 511–518 (2020).

47. M. Singer, C. S. Deutschman, C. W. Seymour, M. Shankar-Hari, D. Annane, M. Bauer, R. Bellomo, G. R. Bernard, J. D. Chiche, C. M. Coopersmith, R. S. Hotchkiss, M. M. Levy, J. C. Marshall, G. S. Martin, S. M. Opal, G. D. Rubenfeld, T. van der Poll, J. L. Vincent, D. C. Angus, The third international consensus definitions for sepsis and septic shock (Sepsis-3). *JAMA* **315**, 801–810 (2016).

48. S. Van Gassen, B. Callebaut, M. J. Van Helden, B. N. Lambrecht, P. Demeester, T. Dhaene, Y. Saeys, FlowSOM: Using self-organizing maps for visualization and interpretation of cytometry data. *Cytometry A* **87**, 636–645 (2015).

49. Q. Xie, J. Klesney-Tait, K. Keck, C. Parlet, N. Borcherding, R. Kolb, W. Li, L. Tygrett, T. Waldschmidt, A. Olivier, S. Chen, G. H. Liu, X. Li, W. Zhang, Characterization of a novel mouse model with genetic deletion of CD177. *Protein Cell* **6**, 117–126 (2015).

50. L. Wang, S. Ge, A. Agustian, M. Hiss, H. Haller, S. von Vietinghoff, Surface receptor CD177/NB1 does not confer a recruitment advantage to neutrophilic granulocytes during human peritonitis. *Eur. J. Haematol.* **90**, 436–437 (2013).

51. F. Passamonti, D. Pietra, L. Malabarba, E. Rumi, M. G. Della Porta, L. Malcovati, M. Bonfichi, C. Pascutto, M. Lazzarino, M. Cazzola, Clinical significance of neutrophil CD177 mRNA expression in Ph-negative chronic myeloproliferative disorders. *Br. J. Haematol.* **126**, 650–656 (2004).

52. Y. Levy, A. Wiedemann, B. P. Hejblum, M. Durand, C. Lefebvre, M. Surenaud, C. Lacabaratz, M. Perreau, E. Foucat, M. Dechenaud, P. Tisserand, F. Blengio, B. Hivert, M. Gauthier, M. Cervantes-Gonzalez, D. Bachelet, C. Laouenan, L. Bouadma, J. F. Timsit, Y. Yazdanpanah, G. Pantaleo, H. Hocini, R. Thiebaut; French COVID cohort study group, CD177, a specific marker of neutrophil activation, is associated with coronavirus disease 2019 severity and death. *iScience* **24**, 102711 (2021).

53. K. Kissel, S. Santoso, C. Hofmann, D. Stroncek, J. Bux, Molecular basis of the neutrophil glycoprotein NB1 (CD177) involved in the pathogenesis of immune neutropenias and transfusion reactions. *Eur. J. Immunol.* **31**, 1301–1309 (2001).

54. R. Grieshaber-Bouyer, P. A. Nigrovic, Neutrophil heterogeneity as therapeutic opportunity in immune-mediated disease. *Front. Immunol.* **10**, 346 (2019).

55. K. Gohring, J. Wolff, W. Doppl, K. L. Schmidt, K. Fenchel, H. Pralle, U. Sibelius, J. Bux, Neutrophil CD177 (NB1 gp, HNA-2a) expression is increased in severe bacterial infections and polycythaemia vera. *Br. J. Haematol.* **126**, 252–254 (2004).

56. M. Bai, R. Grieshaber-Bouyer, J. Wang, A. B. Schmider, Z. S. Wilson, L. Zeng, O. Halyabar, M. D. Godin, H. N. Nguyen, A. Levescot, P. Cunin, C. T. Lefort, R. J. Soberman, P. A. Nigrovic, CD177 modulates human neutrophil migration through activation-mediated integrin and chemoreceptor regulation. *Blood* **130**, 2092–2100 (2017).

57. P. Lalezari, G. B. Murphy, F. H. Allen Jr., NB1, a new neutrophil-specific antigen involved in the pathogenesis of neonatal neutropenia. *J. Clin. Invest.* **50**, 1108–1115 (1971).

58. M. D. Filippi, Neutrophil transendothelial migration: Updates and new perspectives. *Blood* **133**, 2149–2158 (2019).

59. E. Vafadarnejad, G. Rizzo, L. Krampert, P. Arampatzi, A. P. Arias-Loza, Y. Nazzal, A. Rizakou, T. Knochenhauer, S. R. Bandi, V. A. Nugroho, D. J. J. Schulz, M. Roesch, P. Alayrac, J. Vilar, J. S. Silvestre, A. Zernecke, A. E. Saliba, C. Cochain, Dynamics of cardiac neutrophil diversity in murine myocardial infarction. *Circ. Res.* **127**, e232–e249 (2020).

60. F. Y. Chang, M. F. Shaio, In vitro effect of actinomycin D on human neutrophil function. *Microbiol. Immunol.* **34**, 311–321 (1990).

61. L. R. Prince, M. K. Whyte, I. Sabroe, L. C. Parker, The role of TLRs in neutrophil activation. *Curr. Opin. Pharmacol.* **11**, 397–403 (2011).

62. A. Zarbock, M. Allegretti, K. Ley, Therapeutic inhibition of CXCR2 by Reparixin attenuates acute lung injury in mice. *Br. J. Pharmacol.* **155**, 357–364 (2008).

63. S. Bugl, S. Wirths, M. P. Radsak, H. Schild, P. Stein, M. C. Andre, M. R. Muller, E. Malenke, T. Wiesner, M. Marklin, J. S. Frick, R. Handgretinger, H. G. Rammensee, L. Kanz, H. G. Kopp, Steady-state neutrophil homeostasis is dependent on TLR4/TRIF signaling. *Blood* **121**, 723–733 (2013).

64. T. D. Tsourouktsoglou, A. Warnatsch, M. Ioannou, D. Hoving, Q. Wang, V. Papayannopoulos, Histones, DNA, and citrullination promote neutrophil extracellular trap inflammation by regulating the localization and activation of TLR4. *Cell Rep.* **31**, 107602 (2020).

65. V. Papayannopoulos, Neutrophil extracellular traps in immunity and disease. *Nat. Rev. Immunol.* **18**, 134–147 (2018).

66. F. C. M. Kirsebom, F. Kausar, R. Nuriev, S. Makris, C. Johansson, Neutrophil recruitment and activation are differentially dependent on MyD88/TRIF and MAVS signaling during RSV infection. *Mucosal Immunol.* **12**, 1244–1255 (2019).

67. F. V. S. Castanheira, P. Kubes, Neutrophils and NETs in modulating acute and chronic inflammation. *Blood* **133**, 2178–2185 (2019).

68. M. A. Giese, L. E. Hind, A. Huttenlocher, Neutrophil plasticity in the tumor microenvironment. *Blood* **133**, 2159–2167 (2019).

69. S. Salcher, G. Sturm, L. Horvath, G. Untergasser, C. Kuempers, G. Fotakis, E. Panizzolo, A. Martowicz, M. Trebo, G. Pall, G. Gamerith, M. Sykora, F. Augustin, K. Schmitz, F. Finotello, D. Rieder, S. Perner, S. Sopper, D. Wolf, A. Pircher, Z. Trajanoski, High-resolution single-cell atlas reveals diversity and plasticity of tissue-resident neutrophils in non-small cell lung cancer. *Cancer Cell* **40**, 1503–1520.e8 (2022).

70. X. Qi, Y. Yu, R. Sun, J. Huang, L. Liu, Y. Yang, T. Rui, B. Sun, Identification and characterization of neutrophil heterogeneity in sepsis. *Crit. Care* **25**, 50 (2021).

71. Y. Tsukahara, Z. Lian, X. Zhang, C. Whitney, Y. Kluger, D. Tuck, S. Yamaga, Y. Nakayama, S. M. Weissman, P. E. Newburger, Gene expression in human neutrophils during activation and priming by bacterial lipopolysaccharide. *J. Cell. Biochem.* **89**, 848–861 (2003).

72. E. Montaldo, E. Lusito, V. Bianchessi, N. Caronni, S. Scala, L. Basso-Ricci, C. Cantaffa, A. Masserdotti, M. Barilaro, S. Barresi, M. Genua, F. M. Vittoria, G. Barbiera, D. Lazarevic, C. Messina, E. Xue, S. Marktel, C. Tresoldi, R. Milani, P. Ronchi, S. Gattillo, L. Santoleri, R. Di Micco, A. Ditadi, G. Belfiori, F. Aleotti, M. M. Naldini, B. Gentner, E. Gardiman, N. Tamassia, M. A. Cassatella, A. Hidalgo, I. Kwok, L. G. Ng, S. Crippa, M. Falconi, F. Pettinella, P. Scapini, L. Naldini, F. Ciceri, A. Aiuti, R. Ostuni, Cellular and transcriptional dynamics of human neutrophils at steady state and upon stress. *Nat. Immunol.* **23**, 1470–1483 (2022).

73. J. Huang, Z. Zhu, D. Ji, R. Sun, Y. Yang, L. Liu, Y. Shao, Y. Chen, L. Li, B. Sun, Single-cell transcriptome profiling reveals neutrophil heterogeneity and functional multiplicity in the early stage of severe burn patients. *Front. Immunol.* **12**, 792122 (2021).

74. S. F. Marino, U. Jerke, S. Rolle, O. Daumke, R. Kettritz, Competitively disrupting the neutrophil-specific receptor-autoantigen CD177:proteinase 3 membrane complex reduces anti-PR3 antibody-induced neutrophil activation. *J. Biol. Chem.* **298**, 101598 (2022).

75. U. Jerke, S. F. Marino, O. Daumke, R. Kettritz, Characterization of the CD177 interaction with the ANCA antigen proteinase 3. *Sci. Rep.* **7**, 43328 (2017).

76. S. von Vietinghoff, G. Tunnemann, C. Eulenberg, M. Wellner, M. Cristina Cardoso, F. C. Luft, R. Kettritz, NB1 mediates surface expression of the ANCA antigen proteinase 3 on human neutrophils. *Blood* **109**, 4487–4493 (2007).

77. C. J. Kuckleburg, S. B. Tilkens, S. Santoso, P. J. Newman, Proteinase 3 contributes to transendothelial migration of NB1-positive neutrophils. *J. Immunol.* **188**, 2419–2426 (2012).

78. J. Lahoz-Beneytez, M. Elemans, Y. Zhang, R. Ahmed, A. Salam, M. Block, C. Niederalt, B. Asquith, D. Macallan, Human neutrophil kinetics: Modeling of stable isotope labeling data supports short blood neutrophil half-lives. *Blood* **127**, 3431–3438 (2016).

79. C. Eulenberg-Gustavus, S. Bahring, P. G. Maass, F. C. Luft, R. Kettritz, Gene silencing and a novel monoallelic expression pattern in distinct CD177 neutrophil subsets. *J. Exp. Med.* **214**, 2089–2101 (2017).

80. K. Kissel, S. Scheffler, M. Kerowgan, J. Bux, Molecular basis of NB1 (HNA-2a, CD177) deficiency. *Blood* **99**, 4231–4233 (2002).

81. L. Grassi, F. Pourfarzad, S. Ullrich, A. Merkel, F. Were, E. Carrillo-de-Santa-Pau, G. Yi, I. H. Hiemstra, A. T. J. Tool, E. Mul, J. Perner, E. Janssen-Megens, K. Berentsen, H. Kerstens, E. Habibi, M. Gut, M. L. Yaspo, M. Linser, E. Lowy, A. Datta, L. Clarke, P. Flicek, M. Vingron, D. Roos, T. K. van den Berg, S. Heath, D. Rico, M. Frontini, M. Kostadima, I. Gut, A. Valencia, W. H. Ouwehand, H. G. Stunnenberg, J. H. A. Martens, T. W. Kuijpers, Dynamics of transcription regulation in human bone marrow myeloid differentiation to mature blood neutrophils. *cell rep.* **24**, 2784–2794 (2018).

82. Z. Wu, R. Liang, T. Ohnesorg, V. Cho, W. Lam, W. P. Abhayaratna, P. A. Gatenby, C. Perera, Y. Zhang, B. Whittle, A. Sinclair, C. C. Goodnow, M. Field, T. D. Andrews, M. C. Cook, Heterogeneity of

human neutrophil CD177 expression results from CD177P1 pseudogene conversion. *PLOS Genet.* **12**, e1006067 (2016).

83. A. Drewniak, B. J. van Raam, J. Geissler, A. T. Tool, O. R. Mook, T. K. van den Berg, F. Baas, T. W. Kuijpers, Changes in gene expression of granulocytes during in vivo granulocyte colony-stimulating factor/dexamethasone mobilization for transfusion purposes. *Blood* **113**, 5979–5998 (2009).

84. L. Evans, A. Rhodes, W. Alhazzani, M. Antonelli, C. M. Coopersmith, C. French, F. R. Machado, L. McIntyre, M. Ostermann, H. C. Prescott, C. Schorr, S. Simpson, W. J. Wiersinga, F. Alshamsi, D. C. Angus, Y. Arabi, L. Azevedo, R. Beale, G. Beilman, E. Belley-Cote, L. Burry, M. Cecconi, J. Centofanti, A. Coz Yataco, J. De Waele, R. P. Dellinger, K. Doi, B. Du, E. Estenssoro, R. Ferrer, C. Gomersall, C. Hodgson, M. Hylander Moller, T. Iwashyna, S. Jacob, R. Kleinpell, M. Klompas, Y. Koh, A. Kumar, A. Kwizera, S. Lobo, H. Masur, S. McGloughlin, S. Mehta, Y. Mehta, M. Mer, M. Nunnally, S. Oczkowski, T. Osborn, E. Papathanassoglou, A. Perner, M. Puskarich, J. Roberts, W. Schweickert, M. Seckel, J. Sevransky, C. L. Sprung, T. Welte, J. Zimmerman, M. Levy, Surviving sepsis campaign: International guidelines for management of sepsis and septic shock. *Crit. Care Med.* **49**, e1063–e1143 (2021).

85. C. Martin, P. C. Burdon, G. Bridger, J. C. Gutierrez-Ramos, T. J. Williams, S. M. Rankin, Chemokines acting via CXCR2 and CXCR4 control the release of neutrophils from the bone marrow and their return following senescence. *Immunity* **19**, 583–593 (2003).

86. Y. Hao, S. Hao, E. Andersen-Nissen, W. M. Mauck, 3rd, S. Zheng, A. Butler, M. J. Lee, A. J. Wilk, C. Darby, M. Zager, P. Hoffman, M. Stoeckius, E. Papalexi, E. P. Mimitou, J. Jain, A. Srivastava, T. Stuart, L. M. Fleming, B. Yeung, A. J. Rogers, J. M. McElrath, C. A. Blish, R. Gottardo, P. Smibert, R. Satija, Integrated analysis of multimodal single-cell data. *Cell* **184**, 3573–3587.e29 (2021).

87. G. Yu, L. G. Wang, Y. Han, Q. Y. He, clusterProfiler: An R package for comparing biological themes among gene clusters. *OMICS* **16**, 284–287 (2012).

88. M. Gillespie, B. Jassal, R. Stephan, M. Milacic, K. Rothfels, A. Senff-Ribeiro, J. Griss, C. Sevilla, L. Matthews, C. Gong, C. Deng, T. Varusai, E. Ragueneau, Y. Haider, B. May, V. Shamovsky, J. Weiser, T. Brunson, N. Sanati, L. Beckman, X. Shao, A. Fabregat, K. Sidiropoulos, J. Murillo, G. Viteri, J.

Cook, S. Shorser, G. Bader, E. Demir, C. Sander, R. Haw, G. Wu, L. Stein, H. Hermjakob, P. D'Eustachio, The reactome pathway knowledgebase 2022. *Nucleic Acids Res.* **50**, D687–D692 (2022).

89. G. La Manno, R. Soldatov, A. Zeisel, E. Braun, H. Hochgerner, V. Petukhov, K. Lidschreiber, M. E. Kastriti, P. Lonnerberg, A. Furlan, J. Fan, L. E. Borm, Z. Liu, D. van Bruggen, J. Guo, X. He, R. Barker, E. Sundstrom, G. Castelo-Branco, P. Cramer, I. Adameyko, S. Linnarsson, P. V. Kharchenko, RNA velocity of single cells. *Nature* **560**, 494–498 (2018).

90. V. Bergen, M. Lange, S. Peidli, F. A. Wolf, F. J. Theis, Generalizing RNA velocity to transient cell states through dynamical modeling. *Nat. Biotechnol.* **38**, 1408–1414 (2020).

91. F. A. Wolf, P. Angerer, F. J. Theis, SCANPY: Large-scale single-cell gene expression data analysis. *Genome Biol.* **19**, 15 (2018).

92. C. Gene Ontology, The gene ontology resource: Enriching a GOld mine. *Nucleic Acids Res.* **49**, D325–D334 (2021).

93. J. Shilts, Y. Severin, F. Galaway, N. Muller-Sienerth, Z. S. Chong, S. Pritchard, S. Teichmann, R. Vento-Tormo, B. Snijder, G. J. Wright, A physical wiring diagram for the human immune system. *Nature* **608**, 397–404 (2022).

94. A. Janjic, L. E. Wange, J. W. Bagnoli, J. Geuder, P. Nguyen, D. Richter, B. Vieth, B. Vick, I. Jeremias, C. Ziegenhain, I. Hellmann, W. Enard, Prime-seq, efficient and powerful bulk RNA sequencing. *Genome Biol.* **23**, 88 (2022).

95. S. Andrews, FastQC. A quality control tool for high throughput sequence data (Babraham Bioinformatics, 2010).

96. M. Martin, Cutadapt removes adapter sequences from high-throughput sequencing reads. *EMBnet.J.*, (2013).

97. A. Dobin, C. A. Davis, F. Schlesinger, J. Drenkow, C. Zaleski, S. Jha, P. Batut, M. Chaisson, T. R. Gingeras, STAR: Ultrafast universal RNA-seq aligner. *Bioinformatics* **29**, 15–21 (2013).

98. M. I. Love, W. Huber, S. Anders, Moderated estimation of fold change and dispersion for RNA-seq data with DESeq2. *Genome Biol.* **15**, 550 (2014).

99. T. Wu, E. Hu, S. Xu, M. Chen, P. Guo, Z. Dai, T. Feng, L. Zhou, W. Tang, L. Zhan, X. Fu, S. Liu, X. Bo, G. Yu, ClusterProfiler 4.0: A universal enrichment tool for interpreting omics data. *Innovation (Camb)* **2**, 100141 (2021).

100. K. Pekayvaz, A. Leunig, R. Kaiser, M. Joppich, S. Brambs, A. Janjic, O. Popp, D. Nixdorf, V. Fumagalli, N. Schmidt, V. Polewka, A. Anjum, V. Knottenberg, L. Eivers, L. E. Wange, C. Gold, M. Kirchner, M. Muenchhoff, J. C. Hellmuth, C. Scherer, R. Rubio-Acero, T. Eser, F. Deak, K. Puchinger, N. Kuhl, A. Linder, K. Saar, L. Tomas, C. Schulz, A. Wieser, W. Enard, I. Kroidl, C. Geldmacher, M. von Bergwelt-Baildon, O. T. Keppler, M. Munschauer, M. Iannacone, R. Zimmer, P. Mertins, N. Hubner, M. Hoelscher, S. Massberg, K. Stark, L. Nicolai, Protective immune trajectories in early viral containment of non-pneumonic SARS-CoV-2 infection. *Nat. Commun.* **13**, 1018 (2022).

101. C. S. Hughes, S. Foehr, D. A. Garfield, E. E. Furlong, L. M. Steinmetz, J. Krijgsveld, Ultrasensitive proteome analysis using paramagnetic bead technology. *Mol. Syst. Biol.* **10**, 757 (2014).

102. V. Demichev, C. B. Messner, S. I. Vernardis, K. S. Lilley, M. Ralser, DIA-NN: Neural networks and interference correction enable deep proteome coverage in high throughput. *Nat. Methods* **17**, 41–44 (2020).

103. J. Cox, M. Y. Hein, C. A. Luber, I. Paron, N. Nagaraj, M. Mann, Accurate proteome-wide label-free quantification by delayed normalization and maximal peptide ratio extraction, termed MaxLFQ. *Mol. Cell. Proteomics* **13**, 2513–2526 (2014).

104. M. E. Ritchie, B. Phipson, D. Wu, Y. Hu, C. W. Law, W. Shi, G. K. Smyth, limma powers differential expression analyses for RNA-sequencing and microarray studies. *Nucleic Acids Res.* **43**, e47 (2015).

105. L. T. Weckbach, U. Grabmaier, A. Uhl, S. Gess, F. Boehm, A. Zehrer, R. Pick, M. Salvermoser, T. Czermak, J. Pircher, N. Sorrelle, M. Migliorini, D. K. Strickland, K. Klingel, V. Brinkmann, U. Abu Abed, U. Eriksson, S. Massberg, S. Brunner, B. Walzog, Midkine drives cardiac inflammation by promoting neutrophil trafficking and NETosis in myocarditis. *J. Exp. Med.* **216**, 350–368 (2019).

106. D. J. Langford, A. L. Bailey, M. L. Chanda, S. E. Clarke, T. E. Drummond, S. Echols, S. Glick, J. Ingrao, T. Klassen-Ross, M. L. Lacroix-Fralish, L. Matsumiya, R. E. Sorge, S. G. Sotocinal, J. M. Tabaka, D. Wong, A. M. van den Maagdenberg, M. D. Ferrari, K. D. Craig, J. S. Mogil, Coding of facial expressions of pain in the laboratory mouse. *Nat. Methods* **7**, 447–449 (2010).

107. R. Kaiser, R. Escaig, J. Kranich, M. L. Hoffknecht, A. Anjum, V. Polewka, M. Mader, W. Hu, L. Belz, C. Gold, A. Titova, M. Lorenz, K. Pekayvaz, S. Kaab, F. Gaertner, K. Stark, T. Brocker, S. Massberg, L. Nicolai, Procoagulant platelet sentinels prevent inflammatory bleeding through GPIIBIIIA and GPVI. *Blood*, **140**, 121–139 (2022).

108. L. Nicolai, K. Schiefelbein, S. Lipsky, A. Leunig, M. Hoffknecht, K. Pekayvaz, B. Raude, C. Marx, A. Ehrlich, J. Pircher, Z. Zhang, I. Saleh, A. K. Marel, A. Lof, T. Petzold, M. Lorenz, K. Stark, R. Pick, G. Rosenberger, L. Weckbach, B. Uhl, S. Xia, C. A. Reichel, B. Walzog, C. Schulz, V. Zheden, M. Bender, R. Li, S. Massberg, F. Gaertner, Vascular surveillance by haptotactic blood platelets in inflammation and infection. *Nat. Commun.* **11**, 5778 (2020).

109. F. Gaertner, Z. Ahmad, G. Rosenberger, S. Fan, L. Nicolai, B. Busch, G. Yavuz, M. Luckner, H. Ishikawa-Ankerhold, R. Hennel, A. Benechet, M. Lorenz, S. Chandraratne, I. Schubert, S. Helmer, B. Striednig, K. Stark, M. Janko, R. T. Bottcher, A. Verschoor, C. Leon, C. Gachet, T. Gudermann, Y. S. M. Mederos, Z. Pincus, M. Iannacone, R. Haas, G. Wanner, K. Lauber, M. Sixt, S. Massberg, Migrating platelets are mechano-scavengers that collect and bundle bacteria. *Cell* **171**, 1368–1382.e23 (2017).

110. R. Kaiser, A. Anjum, L. M. Kammerer, Q. Loew, A. Akhalkatsi, D. Rossaro, R. Escaig, A. D. zu Senden, B. Raude, M. Lorenz, C. Gold, K. Pekayvaz, T. Brocker, J. Kranich, J. W. Holch, K. Spiekermann, S. Massberg, F. Gaertner, L. Nicolai, Mechanosensing via a GpIIb/Src/14-3-3ζ axis critically regulates platelet migration in vascular inflammation. *Blood* **141**, 2973–2992. (2023).
